# Generation of microsatellite repeat families by RTE retrotransposons in lepidopteran genomes

**DOI:** 10.1186/1471-2148-10-144

**Published:** 2010-05-17

**Authors:** Wee Tek Tay, Gajanan T Behere, Philip Batterham, David G Heckel

**Affiliations:** 1 Centre for Environmental Stress and Adaptation Research, Department of Genetics, Bio21 Molecular Science and Biotechnology Institute, The University of Melbourne, Parkville 3010, Australia; 2 Department of Entomology, Max-Planck Institute for Chemical Ecology, Beutenberg Campus, Hans-Knöll-Straße 8, Jena D-07745, Germany; 3 CSIRO Entomology, Black Mountain Laboratories, Canberra, ACT 2601, Australia

## Abstract

**Background:**

Developing lepidopteran microsatellite DNA markers can be problematical, as markers often exhibit multiple banding patterns and high frequencies of non-amplifying "null" alleles. Previous studies identified sequences flanking simple sequence repeat (SSR) units that are shared among many lepidopteran species and can be grouped into microsatellite-associated DNA families. These families are thought to be associated with unequal crossing-over during DNA recombination or with transposable elements (TEs).

**Results:**

We identified full-length lepidopteran non-LTR retrotransposable elements of the RTE clade in *Heliconius melpomene *and *Bombyx mori*. These retroelements possess a single open reading frame encoding the Exonuclease/Endonuclease/Phosphatase and the Reverse Transcriptase/nLTR domains, a 5' UTR (untranslated region), and an extremely short 3' UTR that regularly consists of SSR units. Phylogenetic analysis supported previous suggestions of horizontal transfer among unrelated groups of organisms, but the diversity of lepidopteran RTE elements appears due to ancient divergence of ancestral elements rather than introgression by horizontal transfer. Similarity searches of lepidopteran genomic sequences in GenBank identified partial RTE elements, usually consisting of the 3' terminal region, in 29 species. Furthermore, we identified the C-terminal end of the Reverse Transcriptase/nLTR domain and the associated 3' UTR in over 190 microsatellite markers from 22 lepidopteran species, accounting for 10% of the lepidopteran microsatellites in GenBank. Occasional retrotransposition of autonomous elements, frequent retrotransposition of 3' partial elements, and DNA replication slippage during retrotransposition offers a mechanistic explanation for the association of SSRs with RTE elements in lepidopteran genomes.

**Conclusions:**

Non-LTR retrotransposable elements of the RTE clade therefore join a diverse group of TEs as progenitors of SSR units in various organisms. When microsatellites are isolated using standard SSR enrichment protocols and primers designed at complementary repeated regions, amplification from multiple genomic sites can cause scoring difficulties that compromise their utility as markers. Screening against RTE elements in the isolation procedure provides one strategy for minimizing this problem.

## Background

Microsatellite genetic markers are based on the properties of SSRs (simple sequence repeats) which are numerous and ubiquitous in the DNA of eukaryotes [1,2]. The basic repeat unit of an SSR is generally considered to be one to six bases long, and an array of two or more basic units repeated in tandem constitutes the SSR. Such SSRs are often miscopied by DNA polymerase, and the resulting high mutation rate, leading to a change in the number of basic units in the array, has proven to be very useful for population genetic studies. The high degree of polymorphism in the size of the tandem array can easily be visualized by gel-separation of PCR products generated by primers placed in the flanking regions, on either side of the SSR.

Another pattern of repetition, however, has proven to be very troublesome in the practical application of microsatellite markers in some organisms, including the insect order Lepidoptera (butterflies and moths). The regions closely flanking one or both sides of the SSR may themselves be highly repeated, dispersed throughout the genome rather than occurring tandemly [3,4]. Primers designed to match these regions, therefore, may generate multiple bands, or even fail to produce discrete visible products because too many different sites are being amplified. Unlike the internally-repeated patterns of the SSRs, there is nothing obvious about the structure of these repeated flanking regions. The fact that they are repeated can be deduced only by comparisons among many flanking sequences, not by any intrinsic pattern of the sequence itself.

Meglécz et al. [5] first described families of similar sequences flanking SSR repeats from two species of Lepidoptera, *Parnassius apollo *and *Euphydryas aurinia*. These similarity patterns fall into two main types, if the location of the SSR is taken into account but its sequence and orientation is ignored. Unilateral repeat families occur on one side only of the SSR, the other side is not repeated (e.g. L1-SSR-R, L2-SSR-S, L3-SSR-T, etc.). In bilateral repeat families, both flanking sides are repeated (e.g. L1-SSR-R1, L2-SSR-R2, L3-SSR-R3, etc.). van't Hof et al. [6] denoted these as asymmetric and symmetric families respectively, however these terms are misleading as there is not necessarily any symmetry between L1 and R1, L2 and R2, etc. These authors examined patterns of flanking SSR families in a third lepidopteran, *Bicyclus anynana*. Using blastn comparisons of these to sequences in GenBank, they defined four "Lepidoptera Specific Core Sequences" (LSCSs) that represented the common elements of four repeated flanking sequences.

The widespread occurrence of these LSCS regions can complicate the use of microsatellite markers in population studies. For example, markers have been developed for the polyphagous noctuid moth *Helicoverpa armigera *by several groups [7-10]. Markers developed for populations of one region (e.g. China) typically have a low success rate in other regions (e.g. Australia). Studies of Australian populations, even using microsatellites developed from those populations, have produced a highly dynamic picture of temporal variation in migration patterns [11-14]. However, a careful re-examination of ten of these markers by Endersby et al. [15] showed that a high proportion of these loci showed allele drop-outs or were not in Hardy-Weinberg equilibrium, undermining their reliability as accurate indicators of population structure.

Based on analysis of whole-genome sequences of several insects, including the lepidopteran *Bombyx mori*, Meglécz et al. [16] showed there were large interspecific differences in frequencies of microsatellites in insect genomes. They confirmed and extended the observations of shared flanking regions among independently isolated sets of microsatellite markers from several different Lepidoptera. They demonstrated a statistical association between SSRs and previously compiled datasets of repeated DNA sequences in six *Drosophila *species and *Anopheles gambiae *(a similar compilation for *B. mori *was not provided). van't Hof et al. [6] also suggested a general association between transposable elements (TEs) and some of the LSCS regions. However, no specific class of TEs was identified as contributing to the observed patterns.

Here we show that a class of non-LTR retrotransposons, RTE elements, are associated with SSR repeats in Lepidoptera and other species. Full-length autonomous elements have low copy numbers in the genome of *Bombyx mori*, however partial elements consisting of the coding sequence of the C-terminal end of the reverse transcriptase protein immediately flanking an SSR are highly abundant. Slippage during reverse transcription and chromosomal integration can increase the number of repeating units within the SSR. These numerous chromosomal integration events of partial elements have peppered the genome with ready-made SSRs flanking an element-specific sequence. These events can account for a substantial fraction of unilateral repeat families recovered in independent efforts from several lepidopteran species.

## Results

### Identification of full-length RTE elements from Lepidoptera

**Figure 1:**
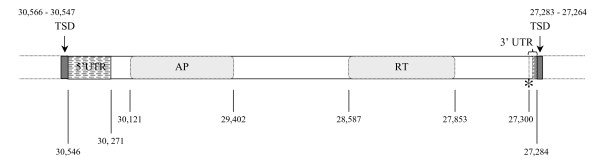
***Heliconius melpomene *HmRTE-e01 non-LTR retrotransposable element identified from a BAC clone **(GenBank:CU462842). Characteristics of the full-length HmRTE-e01 element identified from the *Heliconius melpomene *BAC clone AEHM-22C5 (GenBank:CU462842). The element is inserted in the minus strand of the BAC clone from nucleotide position 30,546 to 27,284. It has a single open reading frame that encodes a 990 amino acid protein sequence. AP marks the Exonuclease/Endonuclease/Phosphatase domain and RT indicates the RT_nLTR_like domain. The element has a 276 bp 5' untranslated region (UTR) and a 14 bp short 3' UTR that includes the (GAA)_2_GA simple repeat units represented by diagonal stripes. The stop codon (TAA) located at 27,300 - 27,298 and is indicated by '*'. Immediately flanking the HmRTE-e01 element are two 20 bp target-site duplication (TSD, represented by filled dark boxes) sequences of 'AGATATACTTCGTTTAAACT'.

The founding member of the RTE clade is the RTE-1 element from *Caenorhabditis elegans *[17,18]. We used its deduced protein sequence (GenBank:AF054983) in tblastn searches of Lepidopteran sequences in GenBank (nr and wgs subsets). A full-length element that we have named HmRTE-e01 sharing all the major features of RTE elements was identified in BAC clone AEHM-22C5 from *Heliconius melpomene *(GenBank:CU462842) (Figure 1). The single open reading frame (ORF) of 990 amino acids contains two conserved domains, an AP (apurinic) endonuclease domain and an RT (reverse transcriptase) domain. Unlike some RTE elements, no skipped stop codons or frameshifts are evident at the 5' end of the coding sequence. The AP endonuclease domain belongs to a family of proteins (pfam03372, Exo_endo_phos, endonuclease/exonuclease/phosphatase family) that includes magnesium dependent endonucleases as well as phosphatases involved in intracellular signalling. The RT domain belongs to a family of proteins found in non-LTR retrotransposons and retroviruses (cd01650: RT_nLTR_like) with activities that include RNA-directed DNA polymerase, DNA-directed DNA polymerase and a ribonuclease that degrades the RNA in a RNA:DNA duplex (RNase H), although no RNase H domain was found in this element. These RTs catalyze the conversion of single-stranded RNA into double-stranded DNA for integration into the host chromosomes. The 3' stop codon (TAA) is immediately followed by a short 14 bp 3'UTR containing a (CTT)_2_CT SSR repeat. A 20 bp target-site duplication (TSD) sequence (AGTTTAAACGAAGTATATCT) immediately follows the (CTT)_2_CT repeat units and also occurs at the 5' end of the element prior to the 5'UTR. The TSD sequences are produced when a staggered cut is made in the double-stranded DNA prior to element insertion, and the two single-stranded regions flanking the new insert are filled in by DNA polymerase [19-21]. The 20 bp TSD sequence can be more easily identified through pairwise sequence alignment between the AEHM-22C5 BAC clone (GenBank:CU462842) and the homologous BAC clone AEHM-7G5 (GenBank:CU462858) that lacks the RTE insertion. The comparison of these two BAC clones from the same population of *H. melpomene *offers a rare opportunity to observe the pre- and post-insertion status of the same genomic region. In the BAC clone AEHM-7G5 the pre-insertion position of the HmRTE-e01 element is between nucleotide positions 22,485 and 22,486, and the 20 bp sequence at 22,466 to 22,485 has become the TSD on both sides of the element in AEHM-22C5. The insertion appears to be recent, as frameshifts and internal stop codons commonly found in other RTE elements have not accumulated, and the population is polymorphic for presence/absence of the element in this genomic locus.

The *C. elegans *RTE-1 sequence was also used to identify 25 full-length elements from the unannotated whole-genome shotgun contigs of *B. mori *(BmRTE-d01 through -d25), the largest number of distinct RTE elements identified from a single species to date. These have the same two conserved protein domains AP and RT, possess various SSR repeats in the short 3' UTR immediately following the stop codon at the end of the RT domain, and are flanked by TSD sequences of differing lengths. Some of the *Bombyx *elements possess an in-frame stop codon or a frameshift within the first 200 bases of the coding sequence, like other RTE elements. The partial RTE sequence identified by Malik and Eickbush [17] next to the cecropin B gene of *B. mori *is represented by the full-length element BmRTE-d05, with some protein sequence differences because of attempts of those authors to correct frameshifts that had been identified in the partial sequence. Three partial RTE sequences identified by Zupunski et al. [22] correspond to the full-length elements BmRTE-d07, -d24, and -d25. Using the full-length *Bombyx *RTE elements as query sequences in blast searches of the *Bombyx *genome contigs yields far more hits of various lengths at the 3' end than at the 5' end, suggesting that the copy number of full-length functional autonomous elements is generally low (around 2 to 4 copies per genome, but up to 16 and 22 copies for BmRTE-d01 and BmRTE-d02 respectively, see Additional File 1), in contrast to frequent insertions (up to 300 in the case of BmRTE-d04) of partial non-autonomous elements with intact 3' ends and flanking SSRs.

### Relation of lepidopteran RTE elements to other non-LTR retrotransposons

**Figure 2:**
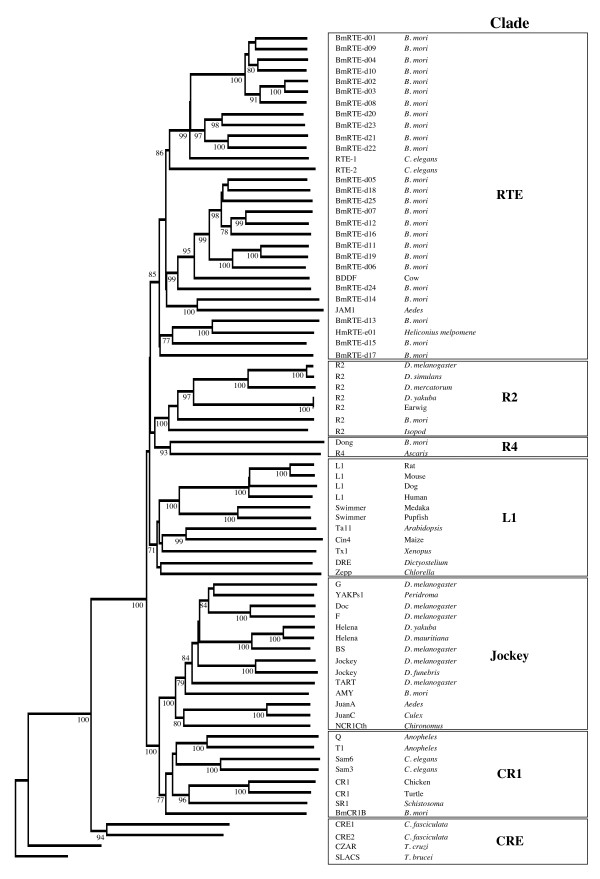
**Phylogenetic analysis of non-LTR retrotransposable elements including novel *H. melpomene *and *B. mori *RTEs**. Phylogenetic analysis of non-LTR retrotransposable elements RT conserved domain from different clades as reported in Figure 3 of Malik et al. [23], and included also the recently described *B. mori *non-LTR CR1B element of the CR1 clade [24]. The tree was constructed using the Neighbour Joining (NJ) method as described in Malik et al. [23] with the CRE element RT conserved domain as outgroups. The NJ tree is a 50% consensus tree, with bootstrap values of >70 from 2,000 bootstrap replications indicated at respective nodes. The RTE clade includes previously described RTE-1, RTE-2, JAM1 and BDDF [23] as well as 25 newly identified elements from *B. mori *and one from *H. melpomene*. With the exception of BmCR1B which was obtained from [24], all amino acid sequences from the RTE, R2, R4, L1, Jockey, CR1 and CRE clades of Malik et al. [23] were from their sequence alignment (EMBL:DS36752).

A phylogenetic analysis of the lepidopteran RTE elements based on alignment against 220 amino acid residues of the RT domain (domain CD01650 in the GenBank Conserved Domain Database) was conducted in comparison with other RTE elements and more generally, other clades of non-LTR retrotransposons (Figure 2). We follow the definition of "clade" used by Malik et al. [23] to represent those retroelements that (1) share the same structural features, (2) are grouped together with ample phylogenetic support, and (3) date back to the Precambrian era. Previously analyzed non-LTR retrotransposons [23] and the *B. mori *CR1 non-LTR element within the CR1 clade [24] were included, with members within the CRE clade (i.e., CRE1, CRE2, CZAR and SLACS) as outgroups. All of the lepidopteran RTE elements cluster together with previously identified RTE elements (RTE-1, RTE-2, BDDF, JAM1) into a single group with a bootstrap value of 85%, placing these newly described lepidopteran elements within the RTE clade [23]. Two major sister groups are evident, consistent with previous analysis [23], one containing RTE-1 and RTE-2 from *C. elegans *and the other JAM1 from the mosquito *Aedes aegypti *and BDDF from the cow *Bos taurus*. A third minor sister group with a more basal position contained three *B. mori *elements (BmRTE-d13, -d15, -d17) and the *H. melpomene *element HmRTE-e01.

**Figure 3:**
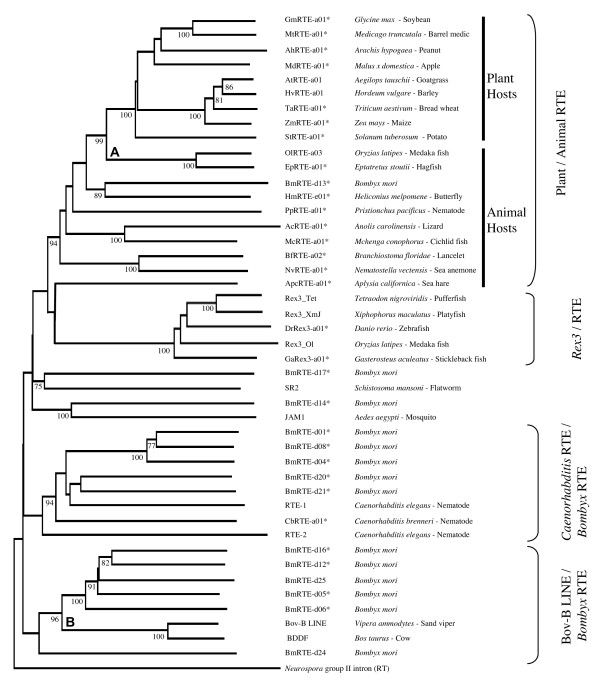
**Neighbour-joining RTE clade phylogenetic tree**. The NJ tree is a 50% consensus tree, with bootstrap values of >70 from 2,000 bootstrap replications indicated at respective nodes. Alignment of complete RT conserved domain used the Kalign sequence alignment program [51,52] in EMBL-EBI. The *Neurospora *Group II intron (GenBank: S07649) was used as the outgroup. 14 representative BmRTE sequences used in Figure 2 have been included, along with newly described RTE elements as listed in the Methods, indicated with an asterisk. The RTE elements were broadly clustered as reported in Figure 5 of [22] although the higher number of BmRTEs identified and included in this analysis, and together with the lower numbers of Bov-B LINEs included have altered the tree topology. Overall, the elements were grouped into four sister groups of animal/plant RTE, *Rex3*/RTE, Bov-B LINE/RTE, and *Caenorhabditis*/*Bombyx *RTE. Although basal to the Plant/Animal RTE and *Rex3*/RTE subgroups, the positions of JAM1 and SR2 remained uncertain [22] due to the lower (<70%) confidence values at the respective nodes. Two nodes representing horizontal transfer events proposed by Zupunski et al. [22] are indicated. (A) from plants to some fishes, (B) from arthropods to reptiles and then to ruminant mammals. Note that the medaka fish *Oryzias *has both a *Rex3 *RTE element similar to other fishes, and a plant-like RTE element.

We extended previous phylogenetic analyses of the RTE subgroups using newly identified fish, molluscan, cnidarian, lizard, amphioxus and plant RTE elements (Additional File 2) as well as previously described nematode RTE (RTE-1, RTE-2, [23]), *Aedes *JAM1 [23], *Schistosoma *SR2 [32], *Oryzias *RTE [22], partial RTEs from *Bombyx *[22], and plant RTE (*Aegilops, Hordeum *[22]). This indicates the BmRTE elements are highly divergent, being distributed among the *Caenorhabditis *RTE, Bov-B LINE and Animal RTE subgroups (Figure 3). From the phylogenetic analysis, elements within the RTE clade are grouped into four subgroups of Plant/Animal RTE, *Rex3*/RTE, *Caenorhabditis *RTE/*Bombyx *RTE, and Bov-B LINE/*Bombyx *RTE (Figure 3). HmRTE-e01 and BmRTE-d13 are clustered within the animal RTEs, while BmRTE-d17 and *Schistosoma *SR2, and BmRTE-d14 and *Aedes *JAM1 are placed basal to the *Rex3*/RTE and Plant/Animal RTE subgroups. The pattern of wide RTE diversity within Lepidoptera, and low similarity to other groups, suggests that the main lineages of *Bombyx *RTE elements identified here are ancient and not due to recent horizontal transfer of elements from non-arthropod groups.

In contrast, the high similarity among Bov-B LINEs of reptiles and mammals (*V. ammodytes *Bov-B LINE, *Bos taurus *BDDF) could be explained by horizontal transfer, as previously suggested by Zupunski et al. [22]. These authors also inferred another instance of horizontal transfer from arthropods to reptiles based on similarity between Bov-B from *V. ammodytes *and other reptiles, and partial sequences from *B. mori *they named *Bombyx *Bov-B/RTE (equivalent to our full-length BmRTE-d24 and -d25). We found the reptile/mammal group to cluster additionally with *B. mori *BmRTE-d05, -d06, -d12, and -d16, but outside the other *Bombyx *elements, pointing to this subgroup as the likely source of the horizontal transfer. As also pointed out by Zupunski et al. [22], horizontal transfer between plant and fish RTE elements was also evident (Figure 3), and the distribution of plant-like elements within fishes is wider than previously suspected, as shown by the element we have identified from the hagfish *Eptatretus*.

### Lepidopteran RTE elements in public DNA databases

A few examples of partial RTE elements in non-coding flanking sequences or introns of genes from Lepidoptera have been deposited in public databases. Chen and Li [25] looked for TEs neighbouring cytochrome P450 genes in *Helicoverpa zea*, and recognized a partial RTE element they named HzRTE-1 within the third intron of CYP9A12v3 (GenBank:DQ788839). This partial element was 1,754 bp long, flanked by 10 bp TSDs, encoded an RT domain with 40% amino acid sequence identity to the *C. elegans *RTE-1 (and 78% identity to the last 400 residues of BmRTE-d01, Additional File 4), and was terminated by a region of TGA trinucleotide repeats in the short 3' UTR. Xu et al. [26] discovered genes with similarity to delta-11 desaturases (GenBank:EF113398) implicated in pheromone biosynthesis in *Ostrinia nubilalis *and *O. furnacalis*; each of which was adjacent to a partial RTE element with 78% amino acid identity to the C-terminal 473 residues of BmRTE-d08 (Additional Files 3 and 4). These authors recognized the sequence similarity to the *C. elegans *RTE-1 element, but considered the partial elements from *Ostrinia *to represent a new family which they named *ezi*, although this *Ostrinia *family was clearly clustered within the RTE-1 clade in their phylogenetic analysis [26]. No SSRs were found in the 3' UTR of these elements.

Other instances of lepidopteran sequences in GenBank that we found have not been previously recognized as RTE elements. These include two partial HzRTE-1-like sequences (named HaRTE-t01; see Additional File 3) within the first intron of two different alleles of a cadherin gene from *H*. *armigera *(GenBank:AY714875 and AY714876) (Additional File 4, Figure 4). A 224 bp partial HzRTE-1-like sequence was also found in the second intron of the preproattacin A gene (GenBank:U46130) of the noctuid moth *Trichoplusia ni*. In *B. mori*, RTE elements are common features of genes deposited in GenBank. For example, in the gDNA sequence for cuticle protein genes BMWCP5 - BMWCP2 (GenBank:AB262389), two partial elements (a 442 bp with 97% identity to BmRTE-d09 over the last 137 amino acids, and a 521 bp with 88% identity to BmRTE-d02 over the last 144 amino acids) were identified, with both partial elements flanked by TSDs and (TGA)n SSR units at the 3' UTR (Additional File 4, Figure 4). Other lepidopteran species with significant gDNA homology to RTE elements (but not as microsatellite DNA loci) included the prophenoloxidase-activating proteinase-1 gene from *Manduca sexta *(GenBank:AY789465), the hemolymph storage protein 2 gene from *Samia cynthia *(GenBank:AB288052), the farnesyl diphosphate synthase gene from *Choristoneura fumiferana *(GenBank:AY962308), the arylphorin gene of *Galleria mellonella *(GenBank:M73793), a BAC clone from *Heliconius numata *(GenBank:CU655868) and a larval serum protein gene from *B*. *mandarina *(GenBank:AY172028) (Additional File 4).

**Figure 4:**
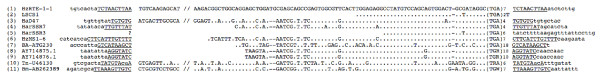
**Sequence alignment between HzRTE-1-1, the LSCS 1, selected lepidopteran microsatellite DNA loci and gDNA sequences**. Alignment between (1) the partial *Helicoverpa zea *HzRTE-1 element ([25], minus bases 996 to 2,632), (2) the Lepidoptera Core Specific Sequence 1 (LSCS1) of van't Hof et al. [6], and selected examples of lepidopteran GenBank entries (3 - 11). HaD47 (GenBank:AY497338, [9]), HarSSR3 (GenBank:AJ504787, [8]), HarSSR7 (GenBank:AJ627416, [7]) are *H. armigera *microsatellite DNA markers (3 - 5), (6) HzMS1-6 (GenBank:EF152206, [28]) and (7) BA-ATG230 (GenBank:DQ225294, [6]) are markers from *H. zea *and *Bicyclus anynana *respectively. (8) and (9) are identical HaRTE elements (HaRTE-t01, Additional File 3) from introns in a cadherin gene of *H. armigera *(GenBank:AY714875 and GenBank:AY714876). (10) belongs to a 224 bp long partial RTE element in *Trichoplusia ni *(GenBank:U46130), and is located within the 2^nd ^intron of the Preproattacin A gene from positions 908 to 1,131 (nucleotides 920 to 1,028 not shown). (11) is a partial 442 bp long RTE element in *B. mori *(GenBank:AB262389) and is located within positions 89,594 to 90,035 (nucleotides 89,606 to 89,930 not shown). TSD sequences are underlined, unique flanking sequence is shown in lower case. Bases identical to the HzRTE-1 sequence are denoted by dots, small gaps inserted for alignment purposes are indicated by dashes, and large gaps in the sequence are represented by '//'.

### RTE elements in Lepidopteran microsatellite clones

The largest collection of microsatellite markers for any single Lepidopteran species was assembled by Miao et al. [27] in *B. mori*. More than 13,600 positive clones were identified by hybridization, yielding 2,690 confirmed by sequencing and 555 polymorphic markers that were used to construct a linkage map. Of these, 518 were deposited in GenBank, and we identified 64 containing fragments of one or more of the BmRTE elements by BLAST. Thus about 12% of this collection of microsatellite markers contained one or more RTE elements.

In their survey of repeated flanking sequences in Lepidopteran microsatellite markers, van't Hof et al. [6] defined four different Lepidopteran Specific Core Sequences (LSCS) that were represented in a large number of clones. Translation of the reverse complement of their 44 bp LCSC1 in the second reading frame produces a protein sequence identical to the last 12 residues of BmRTE-d02. Moreover, the nucleotide sequence is identical to the corresponding region of HzRTE-1. Thus the class of unilateral repeated microsatellites similar to LCSC1 has an RTE element flanking one side. Examples (Figure 4) include *H. armigera *microsatellite clones HaD47 (GenBank:AY497338) [9], HarSSR3 (GenBank:AJ504787) [8] and HarSSR7 (GenBank:AJ627416) [7], the *H. zea *HzMS1-6 microsatellite marker (GenBank:EF152206) [28], and the *B*. *anynana *microsatellite marker BA-ATG230 (GenBank:DQ225294) [6]. TSD sequences were identified for HzMS1-6, HaD47, HarSSR7, and BA-ATG230.

**Figure 5:**
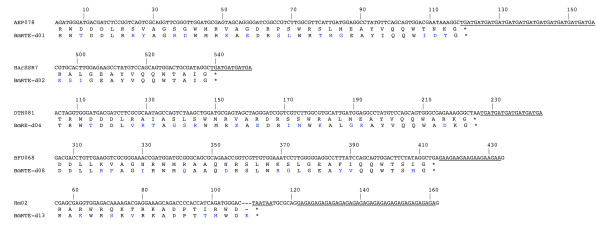
**Protein sequence alignments of translations of selected lepidopteran microsatellite loci and various BmRTEs 3' termini**. Five examples of lepidopteran microsatellite loci with significant amino acid similarity to the translated C-terminal amino acid sequences of various *Bombyx mori *RTE elements are shown. Stop codons adjacent to microsatellite repeat units and gaps inserted for alignment purpose are indicated by '*' and '-' respectively. Nucleotide positions are numbered according to GenBank entries of microsatellite DNA loci. Amino acid residue mismatches are indicated in blue, microsatellite DNA SSR units are underlined. Identity and E-values obtained by stand-alone blastx search against the 25 BmRTEs. Microsatellite DNA loci are: AEP078 (DQ380851, *Arhopala epimuta*, 66% identity to BmRTE-d01, E-value = 7e-13), HarSSR7 (AJ627416, *Helicoverpa armigera*, 80% identity to BmRTE-d02, E-value = 1e-04), DTH081 (DQ380790 reverse complemented, *Drupadia theda*, 62% identity to BmRTE-d04, E-value = 2e-11), BFU068 (DQ393655, *Busseola fusca*, 72% identify to BmRTE-d08, E-value = 6e-14), and Hm02 (DQ020073, *Heliconius melpomene*, 72% identity to BmRTE-d13, E-value = 2e-05).

The 3' ends of RTE elements are widespread in microsatellites isolated from many lepidopteran species. Figure 5 shows representative samples from 5 different species, translated and aligned with the most similar 3' end of a *B. mori *RTE element. The high similarity at the protein level and the SSRs immediately following the stop codon are evident. Different RTE elements can account for the subgroups 2A and 2B of microsatellites of *Bicyclus anynana *previously described by van't Hof et al. [6] (Additional File 3).

Additionally, 165 related non-redundant lepidopteran microsatellite DNA loci were identified by tblastn searches based on protein sequences of the full-length HmRTE-e01 element, 13 full-length elements BmRTE-d01 to -d13, one partial HaRTE-t01 and six partial BaRTE elements (BaRTE-d01 to -d06; Additional File 3). A further 29 microsatellite loci with significant threshold values were identified from blastn searches of LSCS1, HzRTE-1-1 and McRTE-t01 partial RTE elements. Lepidopteran RTE protein and DNA sequence homology searches therefore identified association between microsatellite DNA loci and the non-LTR RTE elements from a total of 22 species from superfamilies Papilionidea (*B*. *anynana*, *H*. *melpomene*, *Papilio zelicaon*, *Arhopala epimuta*, *P*. *apollo*, *Drupadia theda*, *M*. *cinxia*, and *E*. *aurinia*), Hesperiidae (*Erynnis properties*), Geometroidea (*Chiasmia assimilis*, *Biston betularia*), Noctuoidea (*H*. *armigera*, *H*. *zea*, *Arctia caja*, *Busseola fusca*), Bombycoidea (*B*. *mori*), Pyraloidea (*O*. *nubilalis*), Tortricoidae (*Cydia pomonella*, *Lobesia botrana, Tortrix viridana*) and Yponomeutoidea (*P*. *xylostella*, *Yponomeuta padellus*). A wide diversity of SSR units for the reported microsatellite loci was identified (i.e., di-, tri-, tetra- and pentameric nucleotide SSR units of (TGATG)_n_, (TAGA)_n_, (TTTA)_n_, (TGA)_n_, (TGG)n, (ATT)_n_, (GAA)_n_, (TAT)_n_, (TG)_n_, (AT)_n_, (GA)_n_; Additional File 5). The numbers of SSR units varied greatly from two to > 35, and included perfect, compound and imperfect SSR units.

In various reported microsatellite DNA loci (e.g., in *B*. *mori*, (GenBank:DQ242755), (GenBank:DQ242911); *A*. *caja*, (GenBank:AJ809357); *L*. *botrana*, (GenBank:AY150998); *B*. *betularia*, (GenBank:AY190970, AY226154); and *C*. *pomonella*, (GenBank:DQ393953)), RTE-like sequences were present but they did not correspond to the 3' end of the element and were not immediately adjacent to the SSR units. These likely correspond to ancient RTE insertions that have been interrupted by later insertions of other elements (Table 1). Multiple insertion events in close proximity by different RTE elements were also detected. In *B*. *anynana *BA-ATG244 (GenBank:DQ225299), opposite orientations of BaRTE-d06 and BaRTE-d03 elements were identified and included the repeat motifs (KAT)_4 _and (TGA)_26 _respectively (Table 1). In the partial *B*. *anynana *gDNA clone BA-ATG1 (GenBank:AY785062), double insertions by BaRTE elements were detected at both 5' and 3' termini. The 5' terminal partial BaRTE-d06 element was located at nucleotide positions 3 to 41 and included the (TGA)TA(TGA)_11 _SSR units. At the 3' terminus of (GenBank:AY785062) we identified the BaRTE-d03 element at nucleotide positions 339 to 383, however no SSR units was found (Table 1).

**Table 1:** Examples of RTE insertions in lepidopteran microsatellite loci lacking SSR units at the 3' UTR.

Accession Number	RTE	% identity	E-value	Length (bp)	Nucleotide position	SSR units	Associated microsatellite locus	Host
DQ242755	BmRTE-d08	76%	6e-22	63	79 .. 141	none	S1818-F	B. mori
		59%	6e-22	183	152..334			
AJ809357	BmRTE-d01	73%	3e-27	189	191 .. 3	none	Acaja14-(GA03G02)	*A. caja*
AY150998	BmRTE-d05	62%	8e-25	237	230 .. 466	none	F-10 T7	*L. botrana*
AY190970	BmRTE-d09	87%	4e-15	117	364 .. 480	none	BBCAA8G1- C3	*B. betularia*
AY226154	BmRTE-d09	87%	4e-15	117	364 .. 480	none	*B. betularia *microsatellite	*B. betularia*
DQ393953	BmRTE-d10	48%	4e-11	144	146 .. 3	none	CP6.89	*C. pomonella*
DQ225299	BaRTE-d06	100%	1e-07	48	3 .. 50	(KAT)_4_	BA-ATG244	*B. anynana*
	BaRTE-d03	60%	2e-05	60	387 .. 328	(TGA)_26_		
AY785062	BaRTE-d06	100%	2e-05	39	3 .. 41	(KAT)_12_	BA-ATG1	*B. anynana*
	BaRTE-d03	80%	4e-05	45	383..339	none		

Nucleotide positions within the microsatellite loci DNA sequences where respective RTEs were located, and the approximate lengths of the partial RTEs are also provided. Amino acid sequence identity (% Identity) and E-values were ascertained by stand-alone blastx searches against BmRTEs and partial RTEs amino acid sequences (Additional File 3). Multiple insertions of RTEs in close proximity within individual microsatellite DNA loci (DQ225299, AY785062) are also shown.

Note: IUB genetic code 'K' used for (G/T).

Overall, of 2,183 lepidopteran microsatellite sequences deposited in GenBank, 218 or almost 10% were found to contain an RTE element (Additional File 6). The top 16 lepidopteran species accounted for 1,723 microsatellites, 202 or almost 12% of which contain RTE elements. There is some heterogeneity within this group, with RTE content ranging from 0% to 44%. The remaining species with 27 or fewer microsatellites each account for 460 loci, only 16 of which (3.5%) contain RTEs. These differences could reflect actual species differences in microsatellite frequencies or methodological differences among different researchers; only some of which used enrichment techniques.

### Molecular characterization of HaD47 non-allelic size variants in *H. armigera*

**Figure 6:**
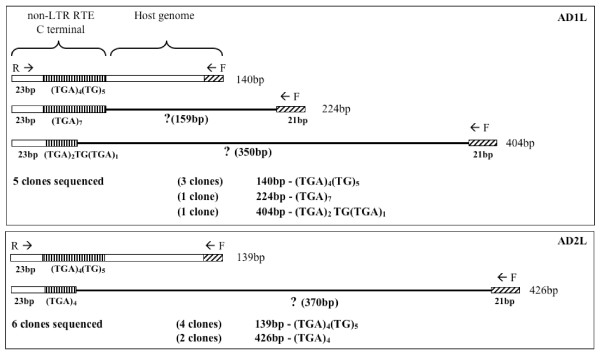
**Non-allelic size variants of *Helicoverpa armigera *microsatellite DNA locus HaD47**. HaD47 microsatellite locus [9] non-allelic size variants in two *Helicoverpa armigera *(AD1, AD2). Size variants of 140 bp (AD1) and 139 bp (AD2) are most similar to the HaD47 published allele (142 bp, nucleotides 131 to 272; AY497338). The partial non-LTR RTE includes the HaD47 reverse primer (R →) and the SSR units are indicated, and the forward primer (← F) is on the host genome. Unknown host genomic sequences of the non-allelic size variants including length (in bp) are indicated by '?'.

The microsatellite marker HaD47 was developed by Scott et al. [9] and used by them in population studies of *H. armigera *in Australia. Sequence comparisons of a flanking region showed high similarity to LSCS1 and microsatellite loci from other Lepidoptera harbouring the 3' end of an RTE element (Figure 4), and so to examine the reproducibility and reliability of this marker, we cloned and sequenced size variants from two *H. armigera *individuals collected from Dalmore, Victoria. Among five clones from individual AD1, three sequence variants of different sizes were identified (140 bp, (GenBank:EU293082); 224 bp, (GenBank:EU293080); 404 bp, (GenBank:EU293081)). The 140 bp band (three clones) possessed an SSR motif of (TGA)_4_(TG)_5_, the 224 bp band (one clone) had (TGA)_7_, and the 404 bp band (one clone) had (TGA)_4_. In the AD2 individual, a 139 bp variant (4 clones, (GenBank:EU293084), with (TGA)_4_(TG)_5_,) and a 426 bp variant (two clones, (GenBank:EU293083)), with (TGA)_4_) were identified. The 140 bp band from AD1 and the 129 bp band from AD2 were very similar to the published sequence of HaD47 and are probably allelic. The other bands, however, show no similarity to these two or to one another in the region to the right of the SSR, except for the 21 bp corresponding to the forward primer (Figure 6). Thus these bands are not likely to be alleles of HaD47 but instead represent amplicons generated by the reverse primer's annealing to independent, unlinked sites of insertion of the 3' end of the RTE element, and the forward primer's annealing to nearby regions with enough sequence similarity to generate a PCR product.

## Discussion

We have identified several RTE non-LTR retrotransposable elements in the Lepidoptera, and provided evidence for their association with unilateral microsatellite DNA families. These lepidopteran RTE elements therefore join a small group of TEs in other organisms (e.g. *Drosophila *[29], humans [30], barley [31]) as being associated with the genesis of microsatellite DNA repeat units. They account for a significant fraction of microsatellite markers isolated by many independent groups working with Lepidoptera (almost 10% overall, ranging from 0% to 44% for different species), and their wide occurrence may be partly responsible for problematical aspects of these markers.

Non-LTR RTE elements have a wide but disjunct phylogenetic distribution in eukaryotes [17,22,23] and have been found in sea urchins (*Strongylocentrotus purpuratus*), nematodes (*C*. *elegans*), blood flukes (*Schistosoma mansoni *[32]), mosquitoes (*Ae*. *aegypti*), amphioxus (*Branchiostoma floridae *[33]), fishes (*Xiphophorus maculatus *[34]), snakes (*Vipera ammodytes *[22]) and mammals (*Bos taurus*) as well as plants [22]. We have identified additional full-length elements from cnidaria, mollusca, and hagfish, as well as 25 from the genome of *B. mori *and one from *H. melpomene*. Lepidopteran RTE elements discovered so far are very diverse but are all grouped into the RTE clade of Malik et al. [23].

Genomic insertion sites of RTEs do not appear to be sequence-specific, and can be discerned only by target-site duplications of 6 - 12 bp after insertion. The typically short 3' UTR often contains di- or tri-nucleotide simple sequence repeats. Truncated RTE elements consisting only of the 3' end of the RT coding sequence followed by the 3' UTR and flanked by target-site duplications are often found in genomes. This configuration suggests that a staggered double-stranded break in the target sequence was made, followed by reverse transcription of the full-length RNA element from the 3' end, which was interrupted before completion so that only the 3' end of the element was finally inserted between the target-site duplications. A large number of truncated elements may accumulate due to the replication efforts of just a few full-length elements, similar to that reported for L1 retrotransposons (reviewed in [20]). For example, in the mosquito *Anopheles gambiae*, the Ag-JAMMIN-2 element is represented by five copies with intact ORFs and about 1,940 truncated elements [35]. In the absence of a full-length RTE sequence as evidence, such truncated RTE elements may be misidentified as short interspersed nucleotide elements (SINEs; [17]) which by contrast are usually derived from abundant cellular RNAs, lack coding potential, possess poly(A) tails, are independently transcribed from their own internal Pol III promoters, and utilize proteins encoded by other retro-elements for their insertion. Similarity of the predicted protein sequences at the 3' ends enabled Malik and Eickbush [17] to recognize that some families previously classified as SINEs were actually truncated RTE elements, just as it has enabled us to recognize RTE elements within isolated microsatellite markers. This is because it is easier to recognize homology of divergent protein sequences than nucleotide sequences.

Some insect TEs show a preference for inserting into specific sequences, and sometimes these sequences are simple sequence repeats. For example, DONG targets the (TAA)_n _of the ribosomal DNA non-transcribed spacer region [36], and the telomere-specific TRAS families target pentameric (TTAGG/CCTAA)_n _SSR units [37]. Since these SSR-target insertion sequences are pre-existing, TE insertion would not increase the abundance of SSRs in the genome, but could increase the abundance of a particular unilateral family of SSR repeats by juxtaposing more copies of the TE next to SSRs existing in different genomic locations. We were not able to find evidence of DONG or TRAS elements in microsatellite markers isolated from Lepidoptera; however it may be that some of the still-uncharacterized LSCS sequence families are formed in this way. By contrast, each new insertion of an RTE element with SSRs in the 3' UTR does increase the abundance of SSRs in the genome. The SSR may have been present in the 3' UTR in the RNA molecule participating in the insertion, or may have been incorporated into the cDNA by slippage of the RT enzyme during reverse transcription. But since the SSR is positioned between the two TSDs, it clearly did not exist in that location prior to the insertion event.

Some fingerprinting methods benefit from the random multiple insertion of transposable elements into genomes [38-40], however, these do not yield single-copy markers. The difficulty of developing lepidopteran microsatellites that function as reliable single-copy codominant markers is widely acknowledged (e.g., [3-5,41,42]). Our sequence comparison of size variants of the microsatellite marker HaD47 showed that at least three different loci were being amplified in different individuals, violating the single-copy assumption. With respect to codominance, a deficiency in observed levels of heterozygosity relative to Hardy-Weinberg expectations has been reported for many published lepidopteran microsatellite DNA markers, and has often been attributed to the presence of null alleles (e.g., [41]). Null alleles can significantly alter the estimation of population substructure patterns by decreasing the within population allelic diversity (e.g., [43]). However, many microsatellite loci reported to exhibit significantly lower than expected levels of heterozygosity are associated with RTE elements, such as in *H*. *armigera *(HarSSR3, [8]; HarSSR7, [7]), *H*. *zea *(HzMS1-6, [28]), *Y*. *padellus *(YP35, [44]), *A*. *epimuta *(AeG5, [45]) and *B. betularia *(Biston 12, [46]). Although it is still unknown whether a systematic RTE-associated effect on heterozygosity exists, the possibility deserves to be taken into consideration along with other explanations such as the Wahlund effect or selection pressure (e.g., insecticide applications on pest species).

One approach to improving the reliability of microsatellite markers is to target single-copy regions in the genome. Widdel et al. [47] used Cot analysis to isolate the slowly-reannealing, low-copy-number fraction of the genome of *Ae. japonicus *from which SSR clones were subsequently isolated. Another approach is to sort through clones already obtained to identify those sharing similarities in their flanking regions and discarding these; Meglécz [48] has described a computer program MICROFAMILY for that purpose. Another alternative for developing codominant markers is to avoid SSRs entirely and to design PCR primers to conserved exons in protein-coding genes to screen for polymorphisms in the intervening intron (EPIC markers, for example as developed as an alternative to microsatellites for *H. armigera*, [49]). A completely opposite strategy is to exploit the high copy number and dispersed distribution of the repeated sequences flanking microsatellites to develop fingerprinting-type, dominant markers [38-40]. Anderson et al. [50] developed two repetitive flanking sequences (ReFS1 and ReFS2) based on microsatellites isolated from *A*. *caja*, where both ReFS markers are part of the LSCS1 sequence with minor variations. They used PCR primers designed to these sequences to discriminate between different species of the moth genus *Schrankia *and to detect interspecific hybrids; dominant markers are sufficient for both purposes. In this application, the sequence conservation of the RTE element during evolution is useful for extending marker utility outside of the species of discovery, and insertions that occur after species divergence are useful for discriminating species; presuming that these insertions have fixed.

New lepidopteran TEs are continuously being identified and characterized (e.g., [24,25]). TEs identified to date in the Lepidoptera are likely to represent only a fraction of the total numbers and types, and some of these others may also be responsible for the genesis of unilateral or bilateral microsatellite DNA families in various lepidopteran species.

## Conclusions

The presence of microsatellite DNA families in the Lepidoptera has mystified many evolutionary and population geneticists over the last decade. Our findings that non-LTR retrotransposable elements of the RTE clade have rendered large proportions of lepidopteran microsatellite DNA markers developed to-date ineffective is indicative of the depth of problems challenging many researchers who have invested resources to developing such molecular genetics tools. Our study will enable TE-affected DNA markers to be recognised across a wide range of organisms, thus allowing informed decisions to be made regarding the utilisation of such DNA markers in future population and evolutionary genetic studies. The presence of RTEs across diverse plant and animal evolutionary lineages implies that the RTE-associated microsatellite DNA families phenomenon may be widespread in many biological systems, and likely to represent one of the many yet unrecognised classes of TEs capable of generating microsatellite DNA families.

## Methods

### Identification of lepidopteran RTE elements

#### In *Bombyx mori*

The *C. elegans *RTE-1 amino acid sequences (GenBank:U58775, U0063, g2253129) as reported in Youngman et al. [18] and Malik and Eickbush [17] were used to identify related RTE elements through tblastn searches in NCBI and KaikoBlast. There are currently three different unannotated genome assemblies available in the wgs section at NCBI. We searched the second assembly with 6-fold coverage (GenBank AADK01000001:AADK01066482) in preference to the first assembly with 3-fold coverage (GenBank BAAB01000001:BAAB01213289) because of the larger contig size of the second assembly; and in preference to the third assembly with 9-fold coverage (GenBank BABH01000001:BABH01088672) because most repeated sequences including RTEs had been masked out and not restored in the third version. All positive gDNA sequences identified in *B*. *mori *were assembled using Sequencher 4.5 (Gene Codes Corporation, Ann Arbor MI, USA) to obtain consensus copies of full-length *B. mori *RTE elements. When an RTE element spanned two or more contigs, only those with >95% sequence identity in the region of overlap were used.

### In *Heliconius melpomene*

We used the *C. elegans *RTE-1 protein sequence (GenBank:AAC72298) in a tblastn search within the NCBI, specifying 'Lepidoptera' within the Entrez query search parameter. Significant positive matches were recorded and the *H. melpomene *BAC clone AEHM-22C5 (GenBank:CU462842) was assessed for putative coding sequences using the NCBI ORF Finder search tool and implementing the default genetic codes option. Protein sequences identified by the ORF search were subjected to blastp searches against the non-redundant (nr) database to ascertain the presence of RTE-like conserved domains. SSR units flanking the 3' UTR of putative RTE-like conserved domains were visually identified. DNA sequences flanking the 3' end of the SSR units and the 5' UTR were manually examined for evidence of TSD sequences. Annotation was added to the GenBank record CU462842 by the submitter, Dr. Simon W. Baxter, Cambridge University.

### Identification of RTE elements from other organisms

The nr, wgs, and dbEST sections of GenBank were searched using tblastn with protein sequences derived from the following: XM_001196835 (*Strongylocentrotus purpuratus*), AJ621035 (Rex3 from *Tetraodon nigroviridis*), and AF332697 (*Vipera ammodytes*) as well as the *B. mori *elements BmRTE-d01 to -d23 and *C. elegans *RTE-1. All new elements identified and their sequences are listed in Additional File 2.

Sequences identified (with GenBank source and base numbers) are for nematodes (*Pristionchus pacificus *PpRTE-a01 ABKE01000427; 33,932-36,769); (*Caenorhabditis brenneri *CbRTE-a01 ABEG01003514; 3,331-6,195), lizard (*Anolis carolinensis *AcRTE-a01 AAWZ01014759; 66,966-69,213), sea slug (*Aplysia californica *ApcRTE-a01 AASC02045436; 3,412-450), lancelet (*Branchiostoma floridae *BfRTE-a02 ABEP02012082; 20,876-17,670), zebrafish (*Danio rerio *DrRex3-a01 BX511127; 5,323-8,013), stickleback fish (*Gasterosteus aculeatus *GaRex3-a01 AANH01001355; 40,384-43,528) cichlid fish (*Mchenga conophorus *McRTE-a01 ABPJ01001712; 1,023-3,566), medaka fish (*Oryzias latipes *Rex3_Ol AB111925; 55,168-57,602, OlRTE-a03 AB207138; 17,235-20,258), pufferfish (*Tetraodon nigroviridis *Rex3_Tet AJ621035; 168-3,337), platyfish (*Xiphophorus maculatus *Rex3-XmJ AF125982; 1-2,625), hagfish (*Eptatretus stoutii *EpRTE-a01 AY965681; 70,600-73,645), sea anemone (*Nematostella vectensis *NvRTE-a01 ABAV01019150; 20,589-23,804), and plants, including peanut (*Arachis hypogaea *AhRTE-a01 FJ654705; 20,923-16,248), soybean (*Glycine max *GmRTE-a01 AC235444; 69,163-66,083), barrel medic (*Medicago truncutala *MtRTE-a01 AC233569; 64,560-61,525), goatgrass (*Aegilops tauschii *AtRTE-a01 AF091802 6,510-9,456), barley (*Hordeum vulgare *HvRTE-a01 AF064563 1,441-2,212), potato (*Solanum. tuberosum *StRTE-a01 AC233388; 58,011-55,175), rice (*Oryza sativa *OsRTE-a01 AAAA02042936), maize (*Zea mays *ZmRTE-a01 AJ850302; 4,233-7,553), bread wheat (*Triticum aestivum *TaRTE-a01 EU835981; 122,283-119,112), apple (*Malus × domestica *MdRTE-a01 AM167520; 22,051-24,641). Additional full-length *B. mori *RTE elements previously identified by Zupunski et al. [22] on the basis of partial sequences (Prof. Dusan Kordis, Jozef Stefan Institute, Ljubljana, Slovenia, pers. comm.) are BmRTE-d12 (partial sequence AV406121), BmRTE-d24 (GU815089, partial sequence AV4052480), and BmRTE-d25 (GU815090, partial sequence AV406078).

### Phylogenetic analysis of putative lepidopteran RTE elements

To determine the evolutionary relationships between the full-length RTE elements identified in this study and previously identified full-length non-LTR retrotransposable elements, complete alignment of RT domain amino acids (EMBL:DS36752) [23] was obtained. The RT conserved domain of non-LTR retroelements from (EMBL:DS36752) [23], all newly assembled *B. mori *RTE elements (BmRTE-d01 to BmRTE-d25; (Genbank:FJ265542 - FJ265564, GU815089- GU815090), and the *H. melpomene *HmRTE-e01 element (GenBank CU462842) were identified using NCBI CD search program against the RT conserved domain CD01650 within the database CDD-31608 PSSMs, prior to a global RT conserved domain alignment using the Kalign program [51,52] in EMBL-EBI. The RT conserved domain of CRE clade elements (CRE1, CRE2, CZAR and SLACS, see Malik et al. [23]) were included as outgroups. For cluster analysis, we implemented the Neighbour-Joining method and conditions (tree bisection-reconnection branch swapping, 2,000 bootstrap resampling, and maximum trees saved at each step limited to five) specified by Malik et al. [23] using PAUP* v4.0b10 [53]. Due to the large data set of Malik et al. [23], we included only elements from the R2, RTE, R4, L1, Jockey, and CR1 clades with our BmRTE and HmRTE elements. The new amino acid alignment file used in the phylogenetic analysis is provided in Additional File 7.

Phylogenetic analysis of RTE subgroups used the RT conserved domain of RTE and *Rex3 *elements that were newly identified in this study (Additional File 2) and also included various elements previously reported by Zupunski et al. [22]. The RT conserved domain were identified using the CD Search program in NCBI http://www.ncbi.nlm.nih.gov/Structure/cdd/cdd.shtml against conserved domain CD01650. Outgroup for the RTE clade phylogenetic analysis used the *Neurospora *group II intron (GenBank:S07649), with the RT conserved domain identified against conserved domain CD01651 using the NCBI CD Search program. Sequence alignment was carried out by including amino acid sequences representative of the conserved domains CD01650 and CD01651 with the identified RT conserved domain amino acid sequences of all RTE, *Rex3 *and Bov-B LINE elements (Additional File 8). Both CD01650 and CD01651 representative sequences were removed prior to phylogenetic analysis using the Neighbour Joining method as described above.

### RTE-like elements in public DNA databases

Using the Lepidoptera Specific Core Sequences 1 to 4 (LSCS 1-4, [6]) we searched the NCBI GenBank non-redundant (nr) genomic DNA database using the Basic Local Alignment Tool blastn [54], and limiting the Entrez query with search term 'Lepidoptera'. Significant matches with expected threshold (E-value) of < E-04 were manually search for evidence of TSD and the presence of 3' SSR units.

### RTE-related lepidopteran microsatellite loci in GenBank

*B. anynana *microsatellite DNA loci described in van't Hof et al. [6] were aligned and grouped using Sequencher 4.5. Consensus 5' flanking sequences prior to the SSR units from grouped microsatellite loci were translated into consensus protein sequences for use in tblastn analyses along with the 'Lepidopteran Microsatellite' Entrez query search term. In *Melitaea cinxia*, a preliminary blastn search using the LSCS1 DNA sequence ('Lepidoptera' as Entrez query search term) identified a significant (E-value = 4e-07) hit (GenBank:DQ389528, MCclone113). SSR units at the 3' UTR, and TSD sequences at 5' and 3' flanking regions were visually identified.

Complete amino acid sequences of RTE elements from *B*. *mori *(BmRTE-d01 to -d13), *H*. *melpomene *HmRTE-e01, and partial RTE-like element protein sequences from *B*. *anynana *and *M*. *cinxia *(Additional File 3), and *H*. *zea *(HzRTE-1-1, [25]) were used as input queries in tblastn searches in NCBI. Accession numbers DQ242653:DQ243686 representing the *B. mori *microsatellites of Miao et al. [27] were searched separately with the BmRTE elements as queries. Partial RTE-like element DNA sequences from *H. armigera *(identified based on sequence homology to HzRTE-1-1), *H. zea*, *M. cinxia *and *B. anynana *were also searched using blastn for homology to lepidopteran microsatellite loci within the GenBank. The search term 'Lepidoptera microsatellite' was used in the Entrez query option in all tblastn and blanstn searches. Due to the general short RTE protein sequences identified from *H. armigera*, *B*. *anynana *and *M*. *cinxia*, tblastn expected threshold of 100 was specified within the 'General Parameters' window. Matrix options implemented were either the default (BLOSUM62), PAM30 (≤ 40 amino acids) or PAM70 (≤ 70 amino acids) settings. All remaining algorithm parameters were as default settings. Default tblastn settings were implemented for full-length *B. mori *(BmRTE-d01 to BmRTE-d13) and *H. melpomene *HmRTE-e01 RTE elements. All significant lepidopteran microsatellite loci matching the complete and partial RTE elements were sorted for non-redundancy based on GenBank Accession numbers. Lepidopteran microsatellite loci from all tblastn and blastn searches that exhibited threshold values of greater than 1E-04 were considered non-significant and thus excluded in all subsequent analyses. All microsatellite loci with significant sequence homologies to partial RTE-like elements of *H. zea*, *H. armigera, B*. *anynana *and *M*. *cinxia*, and to full-length *B*. *mori *and *H*. *melpomene *RTE elements (limiting to within 200 amino acid residues to the 3' terminus) were further characterized with respect to SSR units (Additional File 5, Figure 5) and TSD sequences where possible.

### Molecular characterization of HaD47 alleles

The microsatellite DNA locus HaD47 (GenBank:AY497338) [9] with its reverse primer designed within the LSCS1 is problematic when used in population genetic studies [15] (but see [50]). The molecular dynamics of RTE integration sites within the host genome was investigated by PCR amplification using the HaD47 microsatellite forward and reverse markers following published PCR conditions [9]. DNA from two field-collected *H. armigera *(AD1, AD2, from Dalmore, Victoria, Australia; [55]) were used in this analysis. Post PCR purification, cloning, sequencing and post sequencing DNA analyses of HaD47 microsatellite PCR amplicons followed the protocol of Scott et al. [49]. We randomly selected five and six positively transformed colonies from AD1 and AD2 respectively to confirm the presence of partial RTE-like elements, to ascertain insertion site specificity and to characterize the patterns of associated SSR units.

## List of abbreviations used

**TEs**: Transposable elements; **non-LTR**: non-Long Terminal Repeat; **SSR**: Simple sequence repeat; **UTR**: Untranslated region; **TSD**: Target site duplication; **SNPs**: Single nucleotide polymorphisms; **LSCS**: Lepidoptera specific core sequences; **ORF**: Open reading frame.

## Authors' contributions

DGH and PB identified the lepidopteran microsatellite DNA families problems in published literature. GTB carried out PCR, cloning, sequencing and DNA sequence analysis of *H. armigera *AD1 and AD2 samples. Bioinformatic analyses were carried out by WTT and DGH. The manuscript was prepared by WTT and DGH and critically read by GTB and PB. All authors have read and approved the manuscript.

## Supplementary Material

Additional file 1 **Estimates of full length BmRTE elements in the genome of *Bombyx mori***. NCBI GenBank tblastn searches using *Bombyx mori *RTE elements BmRTE-d01 to -d25, first 50 (5' end) and last 50 (3' end) amino acid residues against the Chinese *B. mori *contigs (GenBank AADK01000001:AADK01066482) to estimate full-length BmRTE copy numbers. Results indicated overall low copy numbers of between 2 to 4 in most of these BmRTE elements within the host genome, although BmRTE-d01 and BmRTE-d02 were higher each with 16 and 22 copies respectively. Click here for file

Additional file 2 **Complete plant and animal non-LTR RTE and *Rex3 *amino acid sequences used for constructing RTE clade phylogeny**. GenBank Accession Numbers, genus and species names, and element names are listed in the Methods. Click here for file

Additional file 3 Partial 3' terminal region including 3'UTR amino acid sequences of non-LTR RTE retrotransposable elements identified in *Helicoverpa armigera*, *Melitaea cinxia *and *Bicyclus anynana*. Click here for file

Additional file 4 **Examples of partial RTE elements within lepidopteran protein encoding DNA sequences deposited in GenBank**. Target site duplication (TSD) sequences are identified and provided where possible, as were associated simple sequence repeat (SSR) units that flanked the 3' UTR of these RTEs. Protein sequence identity (% identity) and E-value to BmRTEs and partial lepidopteran RTEs (Additional File 3) were determined by stand-alone blastx search. Missing SSR units or unidentified TSD are indicated by '?'. Nucleotide position indicates the location where the RTE is inserted in the host genome, and the length (in bp) of the partial RTE identified are also provided. Click here for file

Additional file 5 **A list of 194 lepidopteran microsatellite DNA loci identified from GenBank that showed significant association with lepidopteran non-LTR RTE retroelements**. Lepidoptera microsatellite DNA loci identified by tblastn and blastn searches using RTE elements from *Bombyx mori *(BmRTE-d01 to -d13), *Heliconius melpomene *(HmRTE-e01), *Helicoverpa armigera *(HaRTE-t01), *H*. *zea *(HzRTE-1-1), *Bicyclus anynana *(BaRTE-d01 to -d06, LSCS1) and *Melitaea cinxia *(McRTE-t01) as query sequences. Significance of sequence homology (E-values as obtained from tblastn and/or blastn searches) between RTE elements and lepidopteran microsatellite DNA loci are provided. SSR units associated with individual RTE elements are provided where possible. Note that not all SSR units shown are those reported for the microsatellite loci. Missing SSR units are indicated by (?). Potential 5' and 3' TSD sequences within microsatellite DNA loci are also provided where identified, missing TSD sequences are indicated by '?'. Lower cases within the TSD sequences indicate possible single nucleotide polymorphisms. Click here for file

Additional file 6 **Table showing the top 16 lepidopteran species with the most abundant microsatellite DNA sequences deposited in GenBank (30 June, 2009)**. Using as input queries lepidopteran RTE amino acid sequences in Additional Files 3, 3, and 8 (for tblastn), and the DNA sequences of partial RTEs in Additional File 3 (for blastn), BLAST searches within GenBank against the entrez query search term "Lepidoptera Microsatellite" were carried out. Percentages in parentheses indicate proportion of microsatellite DNA sequences that contained RTEs. The overall proportion of lepidopteran microsatellite DNA sequences containing RTEs remained similar at 9.26% when *Bombyx mori *microsatellite DNA sequences were excluded (due to the majority of these microsatellite DNA sequences being reported for primer regions instead of the actual microsatellite simple sequence repeat unit regions). The 68 *B. mori *microsatellite DNA loci identified to be associated with RTE elements included 64 from 518 loci deposited in GenBank by Miao et al. [27]. Most of these loci are represented by separate forward and reverse sequence reads, so there are about twice as many sequences deposited as numbers of loci. Among the 16 Lepidoptera species with the most abundant microsatellite DNA sequences in GenBank, RTE content ranged from 0% in *Comonympha hero *to 28.75% in *Helicoverpa armigera*, 30.0% in *Busseola fusca *and 43.55% in *Bicyclus anynana*. Click here for file

Additional file 7 **Amino acid alignment of non-LTR retrotransposable element reverse transcriptase (RT) conserved domains by Kalign in EMBL-EBI used to infer phylogenetic relationships between different clades of non-LTR TEs**. Alignment of the Reverse Transcriptase (RT) conserved domain from selected non-LTR retroelements of Malik et al. [23] and Novikova et al. [24], as well as the RT conserved domain of full-length RTE elements from *Bombyx mori *and *Heliconius melpomene *identified from this study. BmRTE-d24 and BmRTE-d25 are new full-length *B. mori *RTE elements that include the partial sequences previously identified by Zupunski et al. [22]. Sequence alignment used default settings of the Kalign program [51,52] in EMBL-EBI. Gaps inserted for alignment purposes are indicated by '-'. Click here for file

Additional file 8 **Amino acid sequence alignment of the Reverse Transcriptase (RT) conserved domain from plant and animal non-LTR RTE clade elements used in RTE clade phylogenetic analysis**. The *Neurospora *Group II intron (GenBank:S07649) RT conserved domain was used as an outgroup. Amino acid sequence alignment was performed using the Kalign program [51,52] in EMBL-EBI using default parameters. The snake (*Vipera ammodytes*) Bov-B LINE, fluke (*Schistosoma*) SR2, plants (*Aegilops*, *Hordeum*), and *Bombyx mori *BmRTE-d24 and -d25 were from [22] with slight modifications. BCCD from cow, JAM1 from *Aedes*, RTE-1 and RTE-2 from *Caenorhabditis elegans *were previously used in the phylogenetic analysis by Malik et al. [23]. All remaining RTE/*Rex3 *elements were as provided in Additional File 2. Click here for file
