# Supplementary material for: Generation of microsatellite repeat families by RTE retrotransposons in lepidopteran genomes
Source: BMC Evol Biol. 2010 May 17;10:144. doi: 10.1186/1471-2148-10-144 (PMC2887409; doi:10.1186/1471-2148-10-144)
Supplement: Additional file 1 — Estimates of full length BmRTE elements in the genome of Bombyx mori. NCBI GenBank tblastn searches using Bombyx mori RTE elements BmRTE-d01 to -d25, first 50 (5' end) and last 50 (3' end) amino acid residues against the Chinese B. mori contigs (GenBank AADK01000001:AADK01066482) to estimate full-length BmRTE copy numbers. Results indicated overall low copy numbers of between 2 to 4 in most of these BmRTE elements within the host genome, although BmRTE-d01 and BmRTE-d02 were higher each with 16 and 22 copies respectively. [file 1471-2148-10-144-S1.PDF]

Additional File 1 (.pdf): Estimates of full length BmRTE elements in the genome of *Bombyx mori*. NCBI GenBank tblastn searches using *Bombyx mori* RTE elements BmRTE-d01 to -d25, first 50 (5' end) and last 50 (3' end) amino acid residues against the Chinese *B. mori* contigs (GenBank AADK01000001:AADK01066482) to estimate full-length BmRTE copy numbers. Results indicated overall low copy numbers of between 2 to 4 in most of these BmRTE elements within the host genome, although BmRTE-d01 and BmRTE-d02 were higher each with 16 and 22 copies respectively.

Bombyx mori RTE element	Number of copies matched at 5' end	Number of copies matched at 3' end
BmRTE-d01	16	191
BmRTE-d02	22	243
BmRTE-d03	5	115
BmRTE-d04	3	300
BmRTE-d05	5	217
BmRTE-d06	3	207
BmRTE-d07	1	184
BmRTE-d08	4	244
BmRTE-d09	2	276
BmRTE-d10	5	152
BmRTE-d11	1	97
BmRTE-d12	1	63
BmRTE-d13	3	114
BmRTE-d14	3	162
BmRTE-d15	7	62
BmRTE-d16	4	81
BmRTE-d17	3	143
BmRTE-d18	5	230
BmRTE-d19	1	191
BmRTE-d20	3	40
BmRTE-d21	5	20
BmRTE-d22	2	41
BmRTE-d23	2	33
BmRTE-d24	4	49
BmRTE-d25	4	173
