# Supplementary material for: Generation of microsatellite repeat families by RTE retrotransposons in lepidopteran genomes
Source: BMC Evol Biol. 2010 May 17;10:144. doi: 10.1186/1471-2148-10-144 (PMC2887409; doi:10.1186/1471-2148-10-144)
Supplement: Additional file 2 — Complete plant and animal non-LTR RTE and Rex3 amino acid sequences used for constructing RTE clade phylogeny. GenBank Accession Numbers, genus and species names, and element names are listed in the Methods. [file 1471-2148-10-144-S2.PDF]

Additional File 2 (.pdf): Complete plant and animal non-LTR RTE and *Rex3* amino acid sequences used for constructing RTE clade phylogeny.

GenBank Accession Numbers, genus and species names, and element names are listed in the Methods.

>PpRTE-a01
MRLRCGECTRFATLNVGTLSGRLLEMVDMIIRRRIEALCVQEIHWKGNESRWANGYQLVCSEGDGLKNGVA
VVLSPRLSSCLVEVTRVDSRLMKVRLDIEGEMICIISAYAPQSGLDKRMKEDFYAKVGEMMEEVSKDDIVI
LGGDLNGHVGRSAEIFGDGGVHGGKGYGRQNVDGLRILEFAQRHDLAVLNTMYEKRKSHLVTFYSGNAQTQ
IDYVMMRKEDRWRVKDVKVIPSECVAPQHKPVICDVRLSGRKSEKRNKLKRMSSKIKWWKLKDPVIRNEFE
RVMIMTGALGIDPLIEEVDVNEVWLETKNAILEGARNVLGVTKGRIKANQESWFWKDESVRVATKEKRIAF
KKWYRMKNNLTKAEYDDKKMECRRVIHEAKMAAYNDLYEMLDGPEGEQALYRLSKSRDREKKDMKEVKSIY
DRNGSVIRKEKEVKERWREYFDDLLNVEKPQALLDDGDVIEQAVLDWSMQEVRWALAKCPWRKAYGPDMIP
VDAWKSMNEAGIWWLTRMFNRIMKEMRMPEDWRRSEIVTLYKGNGDIRECGNYRGIKLISHTMKIYEKCID
RRLREIMTLNPSQYGFVSGSSTAEASFILSQLVDRHIEYRQGICAAFLDLEKAYDRTPRRQIWRSLREKGV
PEIYVRLIKEMYEGASAQVRTPFGPTDDITIKVGVHQGSALSPLLFITVLDSVMGDTMEKAPNCLAYADDL
CLIDTDVGSLERKVQEVQRRLQAGGLTLNTGKTEFMMIGGGQGMMRDMKGEEIKKVEVFRYLGSLIHEDGV
PERDLNNRIKCGCAKFSACSGVLHDKMIRWRLKGKVYRTIIRPAMMYGSEHWALTESMEERLNVAEMKMVR
WIVGIRLRDKVRNDKIRGKIGIAPIVEKIREKRVVWPCKEILQYQPLGRRPAGRPKMSWLKVVKKDLATRR
LTERHALNRELWKEKTQHPDPV*
>CbRTE-a01
EGESVPRTLARRERSRKVMQEGLLRVMNMNVQSIRSVRRLTAFEEAIRKVAFDIIGLSETWRDGKGREILK
NSGHTLLYSGGQFGRKGVGFIVSRQIAPHITNFCAVSQRTCHLDLKINKTLIRVIQAYAPTESSDDDEYEE
FLEELKEQMKEKRGEKRMIIVCGDFNAATGTRQHQNEPGIGPFGFGKRNKRGQMLIDFCSTNGVVVANSFV
KQRKGRKWTWRAPNGRCRKEYDLCIVKSPKIVKTAYAVNRFEFSSDHRMMKIVIKINDFKTRKFYRPQKFE
TDWRKYAEIVEMNKEQIQNEGVTRKYEKMCEILKDARQAATTTQEKEPRFSKETTELFKTRSSLLNKTDPK
SQIELVETNKLLRKYIGRDIEARKFTKYGKAVAARKIHEPTTVSTFYEVLKGNGIATSNPKEIKEAVKEFY
EKLYSSTTRVPRKTPQVTEQAPDILPSEIEYNVSRLKRRKAPGHDGINNNMLRNSLSTTMGNLKEVFSFIL
EQQTIPPTLGDSITTLIPKKGDLRDISNYRPISVLPSTFKLLTRVILGRIQTTLEEQQPPEQAGFRRGYGT
NEHIFNVRMLIQKAREYKINLYIVFVDYQKAYDSVEWNSVFNTLEAHGVDQTYVNTLEAIYKEATSKIKIH
EETTAVSIKRGVRQGCVLSPLLFNAVLEEVFRNLNWEENSEYGLKVNGERITNLRYADDIALIATNKNTMQ
KMMDELVERSREVGLRMNKKKTVALTNISSGPNEDLSIQLDGQKVEMVKSFTYLGCDVSFDVTEKEVQRRI
SCAWGAFTKHRQFLIDRRASPRLKKRVFVACVMPCFLYGCESWSLKLEEKRKLDVAQRRMERAMLGISRID
RIRNDVVAERTRLPRVTEVAWKRKVEWAWKVANTDTRKWSRKIAEWCPLEYKRERGRPVQRWEDEFRERLR
EYGVTGWNWMQMARNKKKEFYHYMTPARNRD*
>AcRTE-a01
NDYIKEDTQFHKRNSHLITYCSGGLETQIDFWMLHWKDHNIWMDSKVIPTDHIAVQHHLHVXGLRINCPRK
NKPVTDIESIKWWKLKELGLTIITPLISCLITCIDLTLEEDWSCTLKAVKMSAASILGNTTPSKTKIDQAT
WCWSEEVQMKIWEKKLKHEHLMKTHWAQDHLAYLSTKWDTTKPVSMAKANYYKKLYENLDTREDKKSVFWL
AKAQNQSTQXSANGTILQRPTNVLERWQQHFNIISNEEFPHPPIHSAAMIEGPVPQIIWDNIKEAITEMKS
SKSTGPGDLPADICKPLKDQSCEWLAELFNSIITENKMPDIWHTSVTVPIWKGIGDVANCSLYRPIRLLCH
TMKIFKHILDNRLREIIMMTENPCGFIKECGAIHATRLLIEKHPEKTRNIHTPFLDVEKAFDWIPPDLIWM
LLRSQRVLEEYIQWKNMLYVNLTSVIRCAGGISDPFPVQAGVLQGLALSPYLFILCMDAITGDIEKPQHWM
LLMRDAMLATESWVELQTQVQVWKNHLQYFGLWLNISKSEYMEYGPTVEDGSININDTDLNKVTSFKYLSS
KVTSTGDVLLDAHGHINGVWMKWRATTDVMCNKKIPIYLKKKIYKTVVRPVAQYATECWSATKAVKQILHI
MEMWILRWSMGISLKDRIPNDTVRSIFGVAPIIEKMKESRLRWFGHVQRSDEGSVAKTMLNKEVAGARPSN
RPKMHWLDNLKHDIPEVRLTMIDATDRNKWKTRSKQADP
>ApcRTE-a01
KIGTWNICSLTNKEPEIMEFMTQNKISIMGITDIRKKGIGTKEIHSNFVLIWSGVRKEQRAEHGVGFIIHP
DKAKEILNTDFISERLIKTSIREGKKTTNYIQVYAPCNDSYSDEEKDSFFEQLSDLISNIPDVEDLYVVGD
FNGRVGERRTPWTKHLGPHSDHRTPCNYNGNHVLELCAEHDLFITNTFFQHRASQIYTWYRWNNIMVWSQI

DFILTRTRMLITITDSRAIPNAGLDTDHRPIITTLTTKKERKPKKHKRQQERLNMHKLGNDQVQAQIRSTL
NEKLGSINSTILTVEEAWDTFKTTLLDTMKEICGTKKTRGTHRKATACWNEEVKDAIKEKKRLYNVWVK*K
NEEDYIKYRLARRHSKKVVITSKEKSWTQYGEKLSETCKTSPREFYEKVKTMRTRDEPYVPTTVINDTNGE
ALHEEKEIIKRWEEYFQELLNPSGVQAQNTQCPFTANYPEHSEPTILESEVKEALKTSPKNKAAGDDGITT
EAILACGETGIHWLTIFFQKAWKERKVPADWQNAIVVPIWKKKGSKKDCNTYRGIPLLSHVGKMWAKILER
RTRAKTEHLLSDAQFGFRKGKGCTDAIFALRQLCERALEYDKDLHLVFVDREKAFDRVNRNKLWKILEQYD
IKGQLLDNIRAIYANSRSAVRTTSGTSDWFPVTSGVRQGCNLSPLLFVIYMDQILKEANPDPEALNELMFA
DDLAMINNKTQLQEHINQLNASCEKHDMKISISKTEVMTISRRPGKVDININGSQLKQSREFKYLGSIFTE
NGKLDREIETRCQRANAVSYQLGPLLKHPNIPMSTKAKLINAIFLPTLTYQCQTWTLTKALERKLVTCEMK
CLRRAVNKTRRDKIKNEVIRDTVGATPVLPHIEKQRVKWFGHLTRMPSNHPALRAYNIKYSGWKSRGRPRR
QWSESVADTLKDHGMSLLQATRLAADRHLCLPATPQGTSGRKK*SKVKYDPSKLPPPPPPFFFF
>BfRTE-a02
MDDVVRALWPGHRLATGKTSGHKTGACPVLFEMESLHHLKFRVGSLNVGTLKGRSSEVVETLTRRRVDLCC
LQETRWSGGLDANQARFVKGKDSRYKFYWCGNKQGQGSVGIMLAEKWVENVFEVQRISDRILLLRLVIGKS
VFTFLSVYAPQIGLSDAEKERFYDQMQSTIAKIPASETVFPIGDWNGHVGEDAQGFEEVHGGHGFGERNTE
GERILEFAVANDLLIGNTLFVKRESHLVTYTSGNHRTQIDYILFRQSFRKAVSNIKVIPGEECARQHQLLV
CDFVVRTPAVRKRKFTPRLRTWKLRDTAVAREFRETFVSKVESVTTDATGGEVEDLWSRLKTPLLEAAASV
CGYSKNHLWKPETWWWDDHVEEAVSKKRARFKVFNSLRKQGKTAVAMVAKTAYNEAKRLAKHAVWLAKSAA
EKETFAVIDPQGADVYRVAKQMERSNQDVVGEMCVRNDAGELSLNDEDKMKAWVEHYNRLLNVEFDWPMDE
LPEAAPVVGPAPPVTTEMISKALSKMRFGKAVGPSGINAEMLKAAGEEGIELTRQLTEAVFRNGTVPVEWE
KSIILSLYKGKGEALDRGNYRGLKLTDHVMKLLERVLDSAIRKMVNIDDLQFAFVPGRGTTDAIFIVRQLQ
EKFIAANKPLYFAFVDLEKAFDRVPRRVLWWALRSLGVEEWAVRVIQAMYANARSRVRVNGQYSEEFGVGV
GVHQGSVLSPLLFILVLEALSREFRTGVPWELLYADDLVIIADTLEECIARLKAWKSGMERKGLRVNMGKT
KIMISGQGLNKLKETGSFPCAVCRSGVGANSIQCTVCNFWVHKRCCGIRGSLLAVQNYTCPRCRGEARPLD
GRPTTQVTVDEVVLDVEASFCYLGDMLCAGGGCELAVTTRSCVAWGKFKKLLPILTSRHLPFKTRGKVFDC
CVRAAMLHGSETWAPTTSDLQRLRRNDRAMIRWICGVKPRDETPSSSLLDKLGILDIISVLQARRLRWFGH
VERSSDCINKITKLKVSGNRGRGRPRKTWRDCVSRDLKECGLTGVDPLDRAKWKRSVKTGRLLLTPASGNP
AAV*
>DrRTE-a01
LGLLIAPQLSRHVLEFSPVNERVASLRLRVGDRSLTVVCAYRPNTSAEYPAFLEYLGGVLEGAQTEDSIVL
LGDFNGHMGNDSDTWRGVIGRNGFPDLNPSGVLLLDFCANHSLSITNTMFEHKDVHQCTWHQDTLGRWSMI
DFVVVSSDLWPYVLDTRVKRGAELSTDHHLVVSWIRWQGGKLDRPGRPKRIVRVLWKCLALPQVRGIFNSH
LWKSFDQIPREAGDMETEWSMFSDSIVNAAVRSCGCKVCVACRVSNPQTRWWTSEVRDAVKLKKESYRAWL
ACRTPKTADAYRRAKRAAARAVAEAKTRAWEEFGETIEEDYRSAPKRFWQTFRRLRGGKHLHIDAVYSGSG
ELLTSTGDVVGRWKEYFEDLLNPTDMSSIEEAETGDAGVNSPITQAEVTEVVCKLRSGKAAGVDEIHPVYL
RSLDVEGLSWLTRLYNIAWRSGTVPLDWATGVVVPIFKKGDRRVCSNYRGITLLNLPGKVYARVLERRIRP
MVEPRIQEEQCGFRPGRGTLDQLYTLTRVLEGSWEYAQPVHMCFVDLKKAFDCVPRGILWRVLGEYGVRGN
LLRAVSSLYEQSRSLVCIAGNKAIRLFSVHVGLWQGCPLSPILFIIFMDRISRCSLGLEGVRFGEHRISSL
LFADDVVLLASSDMDLHHALGRFAAECDAAGMRISTSKSEAMVLHRKKVVCHLQVGGKSLPQVEFKYLWVL
FTSEGRMELEIDRWIGAAAAVMRSMYRSVVVKKELSRKAKLSIYRSIYVPTLTYGHELWIMTERTRSRVQA
AEMSFLRRVAGRTLMDRVRSSDTREDLGVESLLLHIERSQLRWLGHLFRMPPGRLLREVFQACPTGRRPRG
RPRTRWRDYVSRLAWKRLGISPEELEEVSGDREVWGSLLRLLPP*
>GaRex3-a01
ITALAQRASGGRVYRGARPGTARRSDAAAQHSTPHGLTTCGGNRWGRVRCYKGGSDSGGSRRNRPGRQMLA
LGTWNVTSLGGKEPELVREVERYQLDLVGLTSTHSLGSGTKLLDRGWTLFYSGVAHGVRRQAGVGILISPR
LSAATLEFTPVDKRVASLRLRVVGGKTLTVVCAYAPNSSSEYSAFLETLNGVLHGAPVGDSIVLLGDFNAH
VGDDGDNWRGVIGRNGLPDLNPSGCLLLDFCASHGLSITNTMFEHKDVHKCTWYQSTLGQRSMIDFVIVSS
DLRPHVLDTRVKRGAELSTDHHLVVSWVRGWGKTRDRPGKPKRVVRVNWERLEEAPVQKIFNSHLRRSFSH
IPVEVGDIEPEWSMFKTSIAEAAAVSCGLKVLGASRGGNPRTPWWTPVVREAVRLKKEAFRVMISGGTPEA
VAVYRQARRAAASAVMEAKQRVWEEFGVTMEKDFRSAPKCFWKTIRHLRRGKRGTIQAVYSKDGTLLTATE
EVIGRWKEHFEELLNPTTTPSLVEAELEAEEGSSSISLVEVTEVVKQLRSGKAPGIDEIRPEMLKAMGVGG
LSWMTRLFNIAWKSGTVPKEWQIGVVVPLFKKGDQRVCANYRGITLLSLPGKVYSKVLERRVRPIVEPRIE
EEQCGFRPGRGTTDQLFTLSRIIEGAWEYAQPVYMCFVDLEKAYDRVPREILWEVLREYGVRGSLLGAIQS
LYAQSESCVRVLGSKSKAFPVGVGLRQGCALSPILFVVFMDRISRRSRGEEGLQFGGLRISSLLFADDVVL
MASSVCDLQLSLERFAVECEAVGMRISTSKSEAMVLSRKPMDCLLQVGNVSLPQVKEFKYLGVLFTSEGKM
ECEFGRRIGAAGAVLHSLYRTVVTKRELSRKAKLSIYRSIFVPTLTYGHEGWVMTERTRSRVQAAEMGFLR

RVAGVSLRDRVRSSAIREELGVEPLLLCVERSQLRWFGHLVRMPPGRLPREVFQARPAGKRPRGRPRTRWR
DYISALAWERLGIPQSELVDVAREREVWGPLLELLPPRPDPG*AVEDGWMDGWM
>McRTE-a01
LISCYAPWTNCPDEEKTEFWRALDDHLQTISPEEHVAVGGDLNGHVGKGRDGYHRFHGGQGFGTRNDEGCR
VLDYAEAHDLAVTNTFFKKKPAHLITYSSGGRDTQIDYWLVRRPHLSLVTNVKVIPSTNIGPQHRLLVMDL
RLNLGQRRRRTPTTTVEKIKWWRLPECKQQLMDALEHLDVDSNEPVDKAWENIASQIRDAAGXXPLGATKP
GKHFIDKQVWWWNDSVQQTIKEKKSALKVWLQSRSDGDYQQYRALRSAAKKAVAAAKASHYDQLYEELDTP
GGANKIYRLASSRHRATQDIGQVKHIKDANHQTLRDPPAILNRWSEYFSDICNVEFPHQPIHSAPPVPGPV
PPITIKEVETAVKNMKNGKATGPDDIPAEVWKLLGRQGALVLASLFNSIIRDGTVPAAWSTSITVPIWKGK
GDVAECSNYRPIRLLSHTMKVFERIIDARMREIVNLTPNQCGFIRGRGTTDAIHAARLLLEKHREKSKTIH
MAFLDLEKAFDRVPHDLIWHSLRSHGVPEAYVNWTKLLYRQVTSVVRSPVGTSPPFNINVGVHQGSALSPL
LFILCMDTATADLQSPHPWTLLYADDVFLADESRIELQNQTQQWKTRLADFGLRLNTNKTEYLEAGPQTDG
TISVDGEDLAKVPHFKYLGSMISNDGDILLDVRARINAAWMKWRQVTGVLCDRRMPNRLKSKIYKSVVRPV
ALYGSECWPATAKHEQALHTMEMRMLRWCLGLTRWDHVMNTDVRKRMGIAPITDKMQEARLRWYGHVVRSD
ENSVVRTALRLSPQGRRPRGRPKRRWMDRIKDDVKKIEADLTDALDRPKWRRLCRKADPATEREKR*
>NvRTE-a01
MVVVSNSLSEHGTPSNRGVVNGRVGVRRGHRQIDIKRPRLMFRLATLNVGTMKGRSGEVVETITRRKVDLC
CLQETRWRGGSARMISGKDSKFKFFWMGNEKGTGGVGFLLAEKWVESVVEVNRVSDRICVIKINVGASILT
VLSVYAPQSGLDDRSKDAFYDLLQCTISKIADSELLFVCGDFNGHIGSKASAYDGVHGGFGYGERNIEGER
ILEFSIANNFVICNSFFQKRKSHLVTYESGGASTQVDYILSQKCHFKSVHNVKVIPNEECATQHKLLVCDL
AIIPRRQHKHSFTPKLRVWKLRDPEVKSVFLSEVRSEVEGHHHLPSVDDQWLLLKDSLSKVSDKICGYTKK
GHWRKQTWWWDESVSSAINEKRRTWKAWKSGRGSKEEYHKAKRVAKKAVYAAKKQAEVDTFANVEKGEEDI
FRIAKQMKKENKDVVGDCCIRDDHGNLALSTEAKAKAWKDHYERLLNIEFPWSVDDLPLAEPVYGPPILIT
TGMVAEAVSKMKQCKAPGPSGIVAEMLKASAEISCPLLCDIANAIIAEKSIPDDWNLSYIINLYKGKGDAL
DRGSYRGLKLTEHCLKVIERVLEKIIRSLVEIDEMQFGFVHGKGTNDAIFILRQLQEKHLEKGKHLYLGFV
DLEKAFDCVPREVLWWAMRRLSVPEWLVSTVKAMYSNASSRVRVDNSYSDNFNVQVGVHQGSVLSPLLFII
VLEALSCDLRRGCPWELLYADDLVIASDSLENLQQKLMLWKTGMESKGLRVNMKKTKVMVSGPNLETLKDS
GDYPCGVCRKGVGANSILCTCCSHWIHKKCSGIRGRLTPNPSFKCSRCLGVARPIDGRPLDTVQVDDQQLA
VVDSFCYLGDNISAGGGCEAATITRVRVAWGKFRELLPILCSKSLSLHTRGRIYSSCVRGALLYASECWPL
KQTDLARLRRNERAMIRWMCAIKPSNDISTSELRLRLNIEDLDAALRRRRLRWYGHVQRSTSWIRWIRDFI
IEGKRTRGRPRKNWHEVLREDLKLAGLTPDDAQDRVHWKLKLNHAPSDPRPVDN*
>Rex3_Ol
SFVLLGDFNAHVGNDGGTWRGVIGRNGPPDLNPSDVLLLDFCDCHSLSITNTMFEHKGVHQYTWHQDTLER
RSMIDFVVVLGDVWPCVLDNWVKRGAELSTDHYLVVSWIHWRGRRPERLIKPKRTVNVCWERLASPPLGKV
CNSHIRENFKQVLRDVGEIESEWTMFSTSIVSAAAMSCLCPRKVSGACCGGNPRTRWWTPEVRDAVKLKAW
LAYGTPEAADMYRQSKRNWRTRRNSGKPFCASGGGKQFSTGTVFSAGGGLLTLTGDIVGRWKEYFEDLLNP
TDTTSFEEAENEDFGVRLSITRAEVTKVVNELLSGKAPGVDEVRPEYLKFLDAVGVSWLTRLCSIALQSGT
VPLDWQTGVVVPLFKKGGRRVCSNYRGITLLSLPGKVYARVLERRVRPIVKPQIQEQQCSFQPGRGTVDQL
YIISRVLQGLWEFAYTVHMCFVDLEKALDSVPLGILWRLLWENGVRGALLSAVRSLYDWSKSLVRKAGSKS
DLFPVHVGLRQGCPLPPVLFIVFMDRICRRSQGPGGVWFRDHRISSLLFADDVFLLASSGQDLQRTLGRFA
NECEAAGMRFNASKFEAMVLDRRKVVCHV*VG*VSLPQVEEFKYLGVLFTSEGSLEREIDRRIGAASVVMQ
SLHRPVVVKRELSQKAKLSIYRWIFVPILTYGHELWVRIQAAETSFLRRVAGRSLRDRVRSSVTRRERAQN
FFHIQRSQLRWLGHLVRMPLGLLPREIFWRDYVSRLARELIGVPPEELEEVAGAKEVWASLLSLLPLRPSY
G*AEEDGWIFIFCQMLRLV*
>OlRTE-a03
MRIIHNTLDIPDRSRPGLTTTATDAVNLQGVGGNLTTVGRRKRGGRRVRCQREKGKGRNIGLRIGTLNVGT
MTGKGRELADMMERRKVDVLCVQETRWKGSKARSIGGGYKLFYHGVDRKRNGVGVILKGEFVNSVLEVKRV
SDRMMSLKLEIEGVMVNVVSGYAPQVGCELEVKERFWSELDEVIESFPRGERVVIGADFNGHVGEGNRGDE
EVMGRFGVKERNLEGQMVVDFAKRMEMAVVNTYFQKREEHRVTYRSGGRSTQVDYILCRRGHLREVNDCKV
VVGESVARQHRMVVCKMTLEVRKKKIGKTEKKTKWWKLQNEETCEEFRQKLRQVLGGQDELPDDWETTAEI
IRETGRKVLGVSSGKRKDGKETWWWNEEVQECVQRKKRLAKRKWDVERTEEGRQEYKEAQRRVKREVAKAKQ
KAYDELYDRLDTKEGEKDLYRLARQRDRDGKDVQQIRAIKDRDGKVLTTQESVQKRWKEYFEELMNEENDR
ERREEDVVVVEQEVAEIGKDEVRKALKRMKSGKAVGPDDVPVEVWKCLGETAVEFLTRLFNRILESEKMPE
EWRRSVLVPIFKTKGDMQNCSNYRGIKLMSHTMKLWERVVEARLRKKVEICEQQYGFMPRKSTTDAIFALR
MLMEKYREGQKELHCVFVDLEKAYDRVPREELWYCMRSSGVAEKYVRVVQDMYERSMTVVRCAVGQTEEFK
VEVGLHQGSALSPFLFAMLMDRLTDEVRQESPWTMMFADDIVICSESREQVEEQLERWRFALERRGMKVSR
SKTEYMCLNERDQGRSVRLQGTEVKKVQEFKYLGSTVQCDGDCGKEVKRRVQAGWSGWRKVSGVLCDRRVP

ARLKGKVYKTVVRPTLLYGLETVAVRQRQEAEMEVAEMKMLRFSLGMTRLDRIRNEYIRGTAHVACVSDKV
REARLRWFGHVQRRDSGYIGRRMLEMELPGRRARGRPKRRYMDVLTEDMKLANVRVEDVHDRVRWKRMIRC
GDP*WEKPKEKEEE
>Rex3_Tet
NQWSRVSLARTRVTGAPSWSQAWGRGTTASAWWPGFRPWGPAGPSPKRLHVALLQWAHHLQEEPKGSGAKR
VGRRPKGGPWRSDPRLHKLALGTWNVTSLVGKEPELVREVEKFRLDIVGLTSTHSKGSGTTLLERGWTLFY
SGVADGERRRAGVAILVAPQLSACTLEFTPVNERVASLRLRVGGRILTVVCAYGPNGSSVYPAFLESLEGV
LESAPSGGSLVLLGDFNAHVGNDSETWRGVIGKNGPPDLNPSGVSLLDFCARHGMSITNTMFRHKGVHMCT
WHQDTLGRSSMIDFVVVSSDLRPHVLDTRVKRGEELSTDHHLVVSWLRWWGRMPARPGRPKRIVRVCWERL
AESPVRRSFNSHLRENFDHVREEVGDIESEWTMFRASIVEAADRCCGRKVVGACRGGNTRTRWWTPAVRDA
VKLKKESYRAFLACGTPEAAGRYRQAKRSAATAVAEAKTRAWEEFGEAMENDFRTASKRFWTTIRRLRRGK
QCTVNTIYSAGGVLLTSTRDVVDRWREYFEDLLNPTDAPSNEEAEPADLEVDSDISVAEVTEVVKKLLGGK
APGVDEIRPEYLKALDVVGLSWLTRLCSIAWSSGAVPRDWQTGVVVPLFKKGDRRMCSNYRGITLLSLPGK
VYSGVLERRVRRIVEPQIQEEQCGFRPGRGTVDQLYTLSRVLEGAWEFAQPIHMCFVDLEKAFDRVPRGVL
WGVLREYGVSGLLLQAVRSLYNRCQSLVRIAGSKSNSFPVRVGLRQGCPLSPILFITFMDRISRRSYGVEG
VRFGDSRIGSLLFADDVVLLASSTRDLQLPLDRFAAECEASGMRISTSKSEAMVLNRKKVECLLRVGEENL
PQVEEFKYLGVLFTSEGKMEREIDRRIGAASAVMRTLHRSVMVKRELSRKAKLSIYRSIFVPTLTYGHELW
VMTERTRSRVQAAEMSFLRRVAGLSLRDRVRSSVIREELRVEPLLLHIERSQMRWLGHLVRMPPGRLPGEV
FRTRPSGRRPPGRPRTRWRDYVSQLAWERLGIPPEELEEVAGEREVWASLLRLLPPRPDPG*
>AhRTE-a01
RRYCTKVRSFPGSNALYGSSTVSNKQESLHQCPDVMLNEQGFLHFRERTRVNKLVHKVKSKGRSDRKLRFV
TWNIGTLTGKSMEVVDTMTRRKINIMCLQEIKWVGVKVKELDTSGFKLLYTIKVKNRNGVGIIVDKQWKKD
VVDVKRVGDRIISIKLVVEGGTFHVISAYASQAGSDEQHKIRFWEDLESLIQDIPLEYKIFLGGDLNDHVE
REVTGMEVXHRGHGFGVINAEGKTILDFFSTFDILIANTCFKKRNEYLIIYKSGMTSSQIDFFLLRKVDRK
FCINCKIILGESLTTQHRMLVMDFRVEQKLKKRHHTKNPRTRWWRMKGEESRSFIRRVGEEKKWDGNRSTE
EMWREMAEVIKRTTKESFGESKGIGPRDKESWWWNASVQEKIKLKRECFKECSLCRNADNWKKYKPVKKLT
KVDISEVRTRAYEGLYQSLGTKEGEKGIYRIAKSRERRTRDLDQVKCIKDKDGEVLAQKEKINERWKSYFY
ELFNDGHKTLPSLGRLCTREEDQNFDYYRRIQNFEVKETLKKMKNGRAIGLDNILIDVWKGLGEKSINWLT
KLFNKVLRSKKMPNEWRKSTLIPIYKNKGDIQSCENYRGIKLMSDTIKLWERVIEQRLKKETHVTENQFGF
MPGRSTTEAIYLLRRMMERYRSNKRDLHMVFIDLEKAYDRVPREVLWKVLEKKTVRIAYSCAIKDMYDGAT
TSVKTQGGVTKKFLISIGLYQGSSLSPYLFTLVLEVLTKKYIKELVPWCMLFADDIVLMGELREDLNKKLN
LWRKTLKVYGLCISRSKTKYMECKFGLRRENPNIEVKIGENIIQKVKSFKYLGCIIQDNEEIEHDVNHRIQ
AGWSKWRSASSFICDKTVPLKLKGKFYRTAIGPAMLYGTECWTVKGEHEHKLSVSEMKMLRWMSGRMQLDK
IRNEDIRERVGVASIVKKMVESHLWWFGHVRRRPTKHLVRRVDEMEDGQGVKCRGRSKKTIHELVKRDLHV
NGLAVDMIHDITQ*
>GmRTE-a01
MDQLRNNVNARSLPGSNALYGSSTVSKEQGPLHRHPDVVKSKQGFPHFRERVWVKKLVHDRRIRFGTWNIG
TLTGKSMEIVDVMVRRKINFMCLQETKWTGEKAKELDNSGFKLWYTGKIRSRNGVGIIVDKEWKKDVVDVR
RVGDHIIVLKLVVGQDTFNVISGYAPQVGLAEHFKVKFWEDLEGVLQDIPQGEKVFLGGDLNGHVGSVARG
FEGVHGGFGLGEMNGEGKSILEFSEALDLSIANTWFKKREEHLITYKSGGTCSQIDFFLIRKSDRKYCLNC
KVIPGESLTTQHRVLVMDVRIRDRAKRRSPLVAPRIKWWHLKGEKQGIFQQKIWEGWCGQSQGSANDMWNK
MSQEIIKVAKETLGESRGFGPRGKESWWWNENVQSKVRVKKECFKEWSRCRNSETWDKYKIARNETKKAVS
EARTQAFDGLYQALGTRDGERSIYRLAKGRERKTRDLDQVKCVKDEEGKVLVHEKDIKERWKVYFHNLFND
GYGYDSSSLDTREEDRNYKYYRRIQKQEVKEALKRMSNGKAVGPDNIPIEVWKTLGDRGLEWLTELFNEIM
RSKRMPEEWRRSTLVPIYKNKGDIQNCANYRGIKLMSHTMKLWERVIERRLRKETQVTENQFGFMPGRSTM
EAIYLLRRVMEQYRMAQQDLHLIFIDLEKAYDRVPREILWKALEKKGVRVAYIRAIQDMYDRVSTSVRTQG
GESDDFPITIGLHQGSTLSPYLFTLILDVLTEQIQEIAPRCMLFADDIVLLGESREELNERLETWRRALET
HGFRLSRRKSEYMECKFNKRRRVSNSEVKIGDHTIPQVIRFKYLGSVIQDDGEIEGDVNHRIQAGWMKWRK
ASGVLCDAKVPIKLKGKFYRTAVRPAILYGTECWAVKSQHENKVGVAEMRMLRWMCGKTRQDKIRNEAIRE
RVGVAPIVEKMVENRLRWFGHVERRPVDSVVRRVDQMERRQTIRGRGRPKKTIREVIKKDLKINGLDRSMV
LDRTLWRKLIHVADPT*WDKALLLLLLLLLHL*
>MtRTE-a01
KERVVLGLDSQSKTLSNLYDMDQMQNSVNARSLPGSNALYGSSTVSNEQEPLHHHLDVVKIKQGFPHLRER
VRVKKLVHENRIRFGTWNIGTLTGNLSMEVVDTMTRRKINFMCLQETKRVGKKAKELDSSGFKLWYTGEVR
SRNGVGIFVDKEWKKDIVDVKRIGDRIIALKIVVEQDTFNVISAYAPQVGLEAHLKVKFWEELEGLIQDIP
LGEKIFLGGDLNGMWGVFREVSRVCMGGMVSGRLMRRVNPSWMFHRLLILLWLILVSGWKREEHLITFKSG
VSCSHVDFFLIRKSDRKICLDCKVIPGVSLTTQHRVMVMDERVKRRAKRRSQNGAPRIKWRHLKGEKLRIF

QHKILEGDFRLPRGSANDMWERMAHEIKKVAKETLGKSRGFGPSGKESWWWNDSVQSKVRIKRDCFKDWSR
CKNVETGDKYKIARKEAKKAVSEARTQAFEGLYQSLGTKEGEKSIYKLAKGRERKTRDLDQVKCIKDEEGK
ILVQERDIKGRWKKYFHNLFNEDMRSYQTNKLDIGEEDRIYNYYRRIQEHEVREALKRMSSGKAVGPDNIP
IQVWKSLGDRGIVWLTDPFNDIMRTKKMSDEWRRSTLIPIYKNKGDIQNCANYRGIKLTSHTMKLWERVIE
RRLRKETRVTDNQFGFMPGRSTMEAIYLLRRGMERYRTDKKDLHLIFIDLEKAYDRVPREILWQALEKKGV
RIAYIMAIKDMYEGASTSVRTHDETTEDFPTTIGLHQGSTLSPYLFTLVLDVLTEHIQELAPRCMLFADDV
VLVGESREEVNGRLESWRQALEAYGFRLSRSKTEYMECNFSGRRSSSTLEVKVGDHIIPQVTRFKYLGSFV
QNDREIEADVSHRIQVGWLKWRRASGVLCNKKVPLKLKGKFYRTAVRPALLYGTECWAVKSQHENKVSVAE
MRMLRWMSCKTRQDRIRNDNIRERVGVTPTVEKLVDNRLRWFGHVERRPVDAVVRRVDQMEESQVKRGRGR
PRITIRETIRKGFRGQ*
>AtRTE-a01
MLLSPFQHFLFQHWLKTKRRGTQGDVAQLDPVRKLAEPRRIRLGSWNVGSLTGKLRELVDAVVRRGVDILC
VQETKCRGQKAKEVEDTGFKLWYMGTAANRNGVGILINKSLKYGVVDVKRRGDRIILVKLVVGDLVLNVIS
VYAPQVGHNENAKREFWEGLEDMVRSVPIGEKLFIGGDLNGHVGTSNIGFEGAHGGFGYGIKNQEEDVLRF
ALAYDMIVANTLFRKRESHLVTFSSGQHSQIDFILSRREDRCARLDCKVIPGESVVPQHKLVVVDFRFRIR
VQRDKRAKVARMKWWKLKGEVAQAFKERVIREGPWEEGGDADNVWMKMATCIRKVASEECGVSRGWRSEDK
DTWWWNDDVQKAIKEKKDCFRRLYLDRSAVNIEKYKMAKKAAKRAVSEARGRAYEDLYQRLGTKEGERDIY
KMAKIRERGKTRDIGQVKCIKDGADQLLVKDEEIKHRWREYFDKLFNGEDESPTIELDDSFDETIMRFMRR
IQESEVKEALKGGKAMGPDCIPIEVWKGLGDIAIVWLTKLFNLIFRANKMPEEWRRSILVPIINRGDVQSC
TNYHGIKLMSHTMKLWERIIEHRLRRMTSVTKNQFGFMPGRSTMETIFLVRQLMERYREQKKDLHMVFIDL
KKAYNKIPRNVMWWALEKHKVPAKYITLIKDMYDNVVTSVRTSDVDTNDFPIKIGLHQGSALSPYLFALVM
DEVTRDIQGDIPWCMLFVDDLVLVDDSRAGVNNKLELWRQTLESKGFRLSRTKTEYMMCGFSTTRCEEEEV
SLDGQVVPQKDTFRYLGSMLQEDGGIDEDVNHRIKAGWMKWRQASGILCDKRVPQKLGKFYRTAVRPAMLY
GAECWPTKRRHVQQLGVAEMRMLRWMCGHTRKDRVRNDDIRDRVGVAPIEEKLVQHRLRWFGHIQRTPPKT
PVHNGRLKRAENVKRGRGRPNLTWEESVKRDLKVWSITKELAMDRGAWKLVIHVPEP*
>StRTE-a01
SVLRCRFYIRFSIFLVDLVSDGGFESCLRLRPRTREIRGKWGTGTHKLRVGPWNIGSLTGKSIELVKILKK
RKINIACVQETRWVGSKARDVDGFKLWYSGGSRDRNGVGTLVDGDLREQVVEVRRINDRLMLIKLVVGGGT
LSVISAYAPQVGLGEEAKKLFYEDLDEVVRGIPSTEKIVIGGDFNGHIGATSNGFNDVHGGFGFGERNGGG
TSLLDFAKAFELVIANSCFPKKENHLVTFRSSVAKTQIDYLLLRKGDRGLVKDCKVIPSENLTTQHKLLVM
DLVIKRDRRKKMVSDRPRIKWGGLTPDLSREMGEKSSGMGAWSGSEDADTMWNKAASCIREVASKVLGVSR
GKFGGHKGDWWWNGEVQGKVKAKKAAYVKLVECVDEEERRTLKKAYKTSKTEAKSAVTKAKTAAFERLYGE
LGDKGGEKKLYRLAKARERKARDLDQVKCIKDEEGKVLVDETSIKQRWRRYFHKLLNEKGGRDIVLGDLAH
SEGLRDFGYCRCFRIEEVIRAISRMSRGRATGPDEIPVDFWKSTDKAGLEWLTGLFNVIFKTAKMPDEWRW
STMVPLYKNKGDIQNCNNYRGIKLLSHTMKIWERVVEMRVRREVSISENQFGFMPGRSTTEAIHLMRRLVE
KYRERKRDLHMVFIDLEKAYDKVPRNVLWRCLEAKGIPMIYIRAIKDMYGGAKTRVRTVGGDSEHFPVEMG
LHQGSVLSPFLFALVMDELTRSIQERVPWCMLFADDIVLIDETRDRADARLEVWRQTLESKGFKLSRTKTE
YLGCKFSDALDEADGDVRLATQIIPKKESFKYLGFVIQGSGDIDDDVTHRIGVAWMKWRLASGVLCDKKIP
PRLKGKFYRVVVRPALLYGAECWPVKNSHVQKMHVAEMRMLRWMCGHTRSDKIRNEVIREKVGVASVVDKL
REARLRWFGHVRRRCAEAPVRRCEGLVVEGTRRGRGRPKKYWGEVIRQDLAQLRITEDMTLDRKAWRSRIK
VVG**G*
>OsRTE-a01
MVLNEQGPGRLPRDAPCRGLGTVSSEQGPGRHFLSGALHRRPGVVKNEQGSLHFPRRVRRARKLAEPTRFR
VGSWNVGSLTGKLREIAEVAIRRRVNILCVQETKWKGQKAREVEGTGSKLWYTGATSGRNGVGILIDKSLK
NGVVDVRRQGDRIILVRLVVGDLALNVISAYAPQVGLSESTKMQFWEDLDSMVSTVPISEKLFIGGDLNGH
VGASNVGFERVHGGFGYGNRNQEGEDVLSFALAYDLLLANTLFRKRESHLVTFQSGQHSSQIDFILARRED
RRACLDCKVIPGECVVPQHKLMVADFRFRVRVQRDKRAKIARTKWWKLRGEAAQTFKDMMLSEGPWEDGED
ADDMWLKMATCVRKVASEVFGVSRGGKQEAKDTWWWNDEVQMAIKEKKECFKRLHLDRSEANIEGYKLAKR
AAKRAVSVAKGRAYDNLYQRLSTKEGEKDIYRMARIRERKTRDINQIKCIKDGMDRLLVKDEEIKNRWREY
FDKLFNEENGSTLELDDSFDDANRRFVRRIHEAEIAEALKRMKGGKAMGPDGIPIEVWRCLGDRAIVWLTK
LFNLIFRSNKMPEEWRRSILVPIFKNKGDVQSCTNYRGIKLMSHTMKLWERVIEHRLRRVTSVTQNQFGFM
PGRSTMEAIFIIRQLMERHREQKKDLHMVFIDLEKAYDKVPRNVMWWALEKHKVPTKYITLIKDIYKDAMT
VVRTYGGDSSDFPNKIGLHQGSALSPYLFALVMDEVTRDIQSDIPWCMLFADDVVLVDESRAGVNRKLELW
SCGGIRLESKGFRLSRTKTEYMMCDFSGTRYEDGDVRLDGQVVAEKDTFRYLGSMLQKDGDIDEDVRHRIS
AGWLKWRQASGVLCDKRVPQKLKGKFYRTAIRPAMLYGAECWPTKRRHIQQLSVAEMRMLRWFCGHTRRDR
IQNEDIRDRVGVAPIEEKLTQHRLRWFGHVQRRPPEAPVRSGVLQRANNVKRGRGRPKLTWDESVIRDLKE
WNISKDLAMDRSAWRLAINVPEP*SLLSFLLSSNSFSVFFF

>Rex3_XmJ
LPIKSLEGVLESAPPGDSLVLLGDFNAHVGNDSETWRGVVGRNGPPDLNSNGVLLLDFCARHGLSISNTMF
RHKGVHMCTWHQDTLGRSSMIDFVVVSLDLRPDVLDTRVKRGAELSTDHYLMWWGRKPVRPGRPKCVVRVC
WERLAESPVRRSFNSHLRQNFGNTSRGEVGDMESEWTVFRASIVEAAGRNCGRKVVGACRGGNPRTRWWTS
SVRDAVRLKKESYQAFLACGTPEAADGYRQAKRHAARVVAEAKTRAWEEFGEAMEKDFRTASKRFWSTIRH
LGGGKQCGTNTVYSGDGVLLTSTRDVVGRWAEYFEDLLNPTNMPSIEEAEPGDSGLGSPISGDEVAEVVKK
LLGGKAWGVDEIRPEFLKALDVVGLCWLMRLYNIALTSGAVPLDWQTGVVVPLFKKGDRRVCSHYRGVTLL
SLPGKVYSGVLERRVRRIVEPRIQEEQCGFRPGRGTLDQLYTLSRVLEGAWEFAQPVYMCFVDLEKAFDRV
PRGALWGVLREYGVPGPLIRAVRSLYDRCQSLVRIAGSKSGSFPVRVGLCQGCPLSPILFITFMDRISRRS
QGVEGIRFGGLRISSLLFADDVVLLASSGHDLQLSLERFAAECEAAGMGISASKSEAMVLSRKRVECLLRV
KGGVLPQVEEFKYLGILFTNGGRREREIDRRIGAASAVKRALYRSVVVKRELSQKAKLSIYRSIYVPTLIY
GHELWVMTKRTRSRIQAAEMGFLRRVAGLSLRDRVRSSVIWEGLRVEPLLLHIERSQLRWLGHLVRMPSGR
LPGEVFRARPTGRRPQGRPRTRWRDYVSRLAWERLGSPRKELEEVAGEREVWASLLKLLPPGPDLG*
>ZmRTE-a01
VNLPSYIHNKDLSFYCWRYSLIRSNSAYVDWKTSLSRFQHSRSQARLKEEGCVRVRQTNVKNQPNLMEMKP
QRKPLGRNPLSDAPYRNPGMVLNEQGPGRHPSSDALSLDLEPVISEQGSGRRILSGALHRRPGVVKNEQGS
LHLPRRVRRVRKLVEPTRVRVGSWNVGSLTGKLREIVDVAVRRRVNILCVQETKWKGQKAKEVEGTGFKLW
YTGTATNKNGVGVLIDKSLKDGVVDVKRVGDRIILVKLVIGDLVLNVISAYAPQVGLNENSKREFWEGLED
MVSSVPVGEKLFIGGDLNGHVGTSSTSFEGVHGGFGFGTRNQEGEEILNFALAYDMFIANTFFKKRQSHLV
TFSSGQHTSQIDFVLLRKEDRHACLDCKVIPGECVVTQHKLVVADFRFKIRLQRNKHNKVTRTKWWKLKGD
VAQTFKKRVIEEGPWAGEEDANIMWRKMATCIRKIASEEFGLSQGNRREVKDTWWWNEDVQKAIKEKKDCY
KRLHHDKCAENIEKYRIAKKSAKRAVSRARGQAYDDLYQRLDTKQGEKDIYRMAKIRERKTRDVNQVKCIK
DEANQLLVKSEEIKNRWKEYFNKLFNGGNESATIELDEPFDDNNRGFVRRIQEYEVKEALKRMKVGKAMGP
DGIPIEVWRCLGDIAIVWLTKLFNTIFRANRMPDEWRRSTLVPIFKNKGDVQSCTNYRGIKLMSHTMKLWE
RVIEHRLRKMTSVTQNQFGFMPGRSTMEAIFLLRQLMERFREQKKDLHMVFIDLEKAYDKVPRSVMWWALE
KHKVATKYINLIKDMYTNVVTSVRTSDGDTDDFPINIGLHQGSALSPYLFALVIDEVTRDIQGVLPWCMLF
ADDVVLIEESRSGVSQKLELWRQTLEAKGFRLSRSKTEYMKCDFSAMGYEDGDVSLDGQVVPKKDTFRYLG
SMLQKEGDIDEDVSHRIKAGWLKWRQAAGVLCDHRVPRKLKGKFYRTAIRPAMLYGAECWPTKRRHVQQLS
VAEMRMLRWICGHTRRDRVRNDDIRERVGVAPIEEKLMQHRLRWFGHIQRRPEEAPVHIGIIRRPENVKRG
RGRPTLTWTEAVKRDLKEWNIDKELAADRKGWKCAIHVPEP*
>TaRTE-a01
PKRFSLGRNPLSDAPHRNPGVVENGQGPGRHPPGGAPYLDPDTVASERGSGRRILSGALHRRPDVVENEQG
SSHLTRRVRRVRKLAEPRRIRLGSWNVGSLTGKLRELVDAAVRRGVDILCVQETKWRGQKAKEVEDTGFKL
WYTGTAANRNGVGILINKSLKYGVVDVKRRGDRIILVKLVVEDLVLNVISAYAPQVGHNENTKREFWEGLE
DMVRSVPIGEKLFIGGDLNGHVGTSNTGFEGAHGGFGYGIRNQEGDDVLSFALAYNMIVANTLFRKRESHL
VTFSSGQHSSQIDFILSRREHRRACLDCKVIPGESVVPQHKLVVADFRFRIRVQRDKRAKVARTKWWKLKG
EVAQAFKERVIKEGPWEEGGDADNVWMKMATCIRKVASEEFGVSRGRRSEDKDTWWWNDDVQKAIKEKKDC
FRRLYLDRSAHNIEKYKMAKKAAKRAVGEARGRAYEDLYQRLGTKEGERDIYKMAKIRERKTRDIGQVKCI
KDGAGQLLVKDEEIKHRWREYFDKLFNGENESSTIELDDSFDETSMRFVRRIQESEVKEALKRMKGGKAMG
PDCIPIEVWKGLGDIAIVWLTRLFNLIFRANKMPEEWRRSILVPIFKNKGDVQSCTNYRGIKLMSHTMKLW
ERVIEHRLRRMTSVTKNQFGFMPGRSTMEAIFLVRQLMERYREQKKDLHMVFIDLEKAYDKIPRNVMWWAL
EKHKVPAKYITLIKDMYDNVVTSVRTSDVDTDDFPIKIGLHQGSALSPYLFALVMDEVTRDIQGDIPWCML
FADDVVLVDDTSAGVNRKLELWRQTLESKGFRLSRTKTEYMMCGFSTTRCEEEEVSLDGQVVPRKDTFRYL
GSMLQEDGGIDEDVNHRIKAGWMKWRQASGILCDKRVPQKLKGKFYRTAVRPAMLYGAECWPTKRRHVQQL
GVAEMRMLRWMCGHTRKDRVRNDDIRDRVGVAPIEEKLVQHRLRWFGHIQRRPPEAPVHSGWLKRAENVKR
GRGRPNLTWEESVKRDLKDWGITEELAMDRGAWKLAIHVPEP*
>MdRTE-a01
MKTGSVFLRLQFFLKFGGLGLDTSSKEKFWEDLGDLVQGIAQTEKLFIGGDLNGHVGRETGNYGGFHGGHG
FGERNEDGEAILDFAMAYDLFLANTFFKKREEHVITYKSGSSKTQIDFLLMRKGDRITCKDCKVIPGESVA
NQHRLLVMDVHIKRVRQKNKTWKCPRTRWWNLKEEKQAIFKEKVITQCVWDREGEASQMWDSMASCIRKVA
KEVLGESKGFAPHQKESWWWNEEVQTKVKAKKECCKALYKERTDENGERYRKAKQEAKKAVREAKLAAYDD
MYKRLDTKEGELDIYKLARAREKKTRDLNQVRCIKDEDGKVLATENAVKDRWRGYFHNLFNEGHERSASLG
ELSNSEECRNYSFYRRIRKEEVVVALKKMKHRKAIGPDDIPIEVWKVLGETGITWLTDLFNRILKTKKMPN
EWRTSTLVPIYKNKGDVQNCMNYRGIKLMSHTMKLWERVIEHRLRQETRVSDNQFGFMPGRSTMEAIYLLR
RLMERYRDGKKDLHMVFIDLEKAYDRVPRDILWRILEKKGVRVAYIQAIKDMYEGAKTAVRTHEGQTESFP
ITVGLHQGSSLSPYLFALVMDELTGHIQDDIPWCMLFADDIVLIDETQEGVNAKLNLWREVLESKGLRLSR
SKTEYMECNFSANGGQNELGVRIGDQEIPKSDRFRYLGSILQKNGELDGDLNHRIQAGWMKWKSASGVLCD

RRMPLKLKGKFYRTAIRPAMLYGTECWAVKHQHVHKMGVAEMRMLRWMCGHTRKDKIRNEDIRGKVGVAEI
EGKMRENRLRWFGHVQRRPTDAPIRRCDYGTEVQGRRGRGRPRKTLEETLRKDLEYLDLTKDMTQDRAQWR
SKIHIADPTQ*
>EsRTE-a01
MRTTKRISIPDRSRPGLPTTATGTGGQQGTSGNCATAGRTRRRRRGRRVRRQWERGKGRSVEVRVGTLNVG
TMTGKGRELADMMERRKVDILCVQETKWMGSKARSIGGGFKLFYHGVDGRRNGVGIILKEDYVKRVLEVRR
VSDRVMSVKLEIEGVMMNVISAYAPHMGCEMEYKEDFWSELDEVVESVSKDERVVIGADFNGHVGEGNKED
EEVMGRYGLNKRNVEGQMVVEFAKRMEMAVVNTYFKKKEEHRVTYKSGGKCTQVDYILSRRCNLKEISDCK
VVAGESVARQHRLVVCRMTLEVRLRKRVRAEPRTRWWKLKEEDCCVEFREEVRQALDGGKDGCDDWATTAE
VVREIARKVLGVTSGQRKEDKETWWWNEEVQESIRKKGLAKRKWDSQRDEESRQEYKEWRRKAKREVAKAK
KKAYDELYEKLDTKEGEKDLYRLARQRDRAGKDVLQVRVIKDANGNVLTSEESVLRRWMEYFEELMNEENE
RERRLEEVEIVNLEVGWISKDEVRTAMKRMKSGKAVGPDGIPVEAWKCLGEMAVGLLTRLFNKILESERMP
EEWRLSVLVPIFKNKGDVQSCGNYRGIKLLSHTMKLWERVVEARLRGEVTICEQQYGFMPGKSTTDAMFAL
RMLMKRYREGQKELHCVFVDLEKAYDRVPREELWHCMRKSGVVEKYVRIVQDMYEDSVTAVRCAVGMTDRF
KVKVGLHQGSTLSPFLFAMVMDRLTDEIRLESPWTMMFADDIMICRESREQAEASLERWRYALERRGMIVS
RSKTEYMCVNEREGGGMVRLQGVEVGKVDGFKYLGSTVQSNGECGREVKKRVQAGWSGWRRVAGVICDRRV
AARVKGKVYKTVVRPAMLYGLEAVALTKRQEAELEVAELKMLRFSLGVTRMDRIRNEYIRGTAQVGRFGDK
VREARLRWFGHVHRREAEYIGRRMLKMEPPGRRRRGRPKKRFMDAVRADMRVVGVSMEDVEDRAKWRRLIR
CGDP*
>HvRTE-a01
HIKDMYDNVVTSVRTSDGDTDDFPIKIGLHQGSALSPYLFALVMDEVTRDIQGDIPWCMLFADNVVLVDDS
RTGVNRKLELWRQTLKSKGFRLSRTKTEYMRCDFSTTKHEDGGGVSLDGQVAPQKDTFRYLGSMLQKDGDI
DEDVNHRIKAGWMKWRQASGILCDKRVLQKLKGKFYRTMVRPAMLYGAECWPTKRRHVQQLGVAEIRMLRW
MCGHTKKDQIRNDDIRDRVGVAPIKEKLVQHRLRWFGHIQRRPR
