# Supplementary material for: Generation of microsatellite repeat families by RTE retrotransposons in lepidopteran genomes
Source: BMC Evol Biol. 2010 May 17;10:144. doi: 10.1186/1471-2148-10-144 (PMC2887409; doi:10.1186/1471-2148-10-144)
Supplement: Additional file 3 — Partial 3' terminal region including 3'UTR amino acid sequences of non-LTR RTE retrotransposable elements identified in Helicoverpa armigera, Melitaea cinxia and Bicyclus anynana. [file 1471-2148-10-144-S3.PDF]

Additional File 3 (.pdf): Partial 3' terminal region including 3'UTR amino acid sequences of non-LTR RTE retrotransposable elements identified in *Helicoverpa armigera*, *Melitaea cinxia* and *Bicyclus anynana*.

```
LOCUS       HaRTE-t01     90 bp    DNA                        26-Sept-2008

DEFINITION  Helicoverpa armigera RTE-like non-LTR retrotransposon (HaRTE-
            t01), partial. Identified from H. armigera Ecadherin gene
            (AY714876.1) and H. armigera Ecadherin r2 allele (AY714875.1)
ACCESSION   .
VERSION     .
KEYWORDS    Microsatellite DNA families, Transposable elements, non-LTR
            RTE, Lepidoptera, microsatellite DNA genesis
SOURCE      Helicoverpa armigera (Cotton bollworm)
ORGANISM    Helicoverpa armigera
            Eukaryota; Metazoa; Arthropoda; Hexapoda; Insecta; Pterygota;
            Neoptera; Endopterygota; Lepidoptera; Glossata; Ditrysia;
            Noctuoidea; Noctuidae; Heliothinae; Helicoverpa.

COMMENT     .
FEATURES             Location/Qualifiers
     source          AY714875.1, AY714876.1
                     /organism="Helicoverpa armigera"
                     /mol_type="genomic DNA"
     5'TSD           AGGTATCs
     5'UTR           .
     CDS             .
                     /codon_start=1
                     /product="reverse transcriptase, partial"
                     /translation="NRSQWRALGEAYVQQWTAIGW*W*******"
     STOP            .
     3'UTR           .
     (TGA)_site      .
     3'TSD           AGGTATC

BASE COUNT      20 a       14 c       33 g       23 t
ORIGIN
        1 AATCGATCTC AGTGGCGTGC ACTTGGAGAG GCCTATGTCC AGCGGTGGAC TGCGATAGGC
       61 TGGTGATGGT GATGATGATG ATGATGATGA
//
```

```
LOCUS       McRTE-t01     70 bp    DNA                      26-Sept-2008

DEFINITION  Melitaea cinxia RTE-like non-LTR retrotransposon (McRTE-t01),
            partial. Identified from Melitaea cinxia isolate MCclone113
            genomic sequence partial sequences
ACCESSION   .
VERSION     .
KEYWORDS    Microsatellite DNA families, Transposable elements, non-LTR
            RTE, Lepidoptera, microsatellite DNA genesis
SOURCE      Melitaea cinxia gDNA
ORGANISM    Melitaea cinxia
            Eukaryota; Metazoa; Arthropoda; Hexapoda; Insecta; Pterygota;
            Neoptera; Endopterygota; Lepidoptera; Glossata; Ditrysia;
            Papilionoidea; Nymphalidae; Nymphalinae; Melitaea.

COMMENT     Identified from DQ389528.1; 6bp TSD = ATGAGC
FEATURES             Location/Qualifiers
     source          .
                     /organism="Melitaea cinxia"
                     /mol_type="genomic DNA"
     5'TSD           .
     5'UTR           .
     CDS             .
                     /codon_start=1
                     /product="reverse transcriptase, partial"
                     /translation="TSFLTRT*EAFVQQWTAIG*SD"
     STOP            .
     3'UTR           .
     (TGA)_site      .
     3'TSD           .

BASE COUNT      15 a       14 c       19 g       14 t
ORIGIN
        1 TCACGTCATT TTTGACGCGA ACTTGAGAGG CCTTTGTCCA GCAATGGACT GCGATAGGTT
       61 GAAGTGATGA
//
```

```
LOCUS       BaRTE-d01     33 bp    mRNA                    26-Sept-2008

DEFINITION  Bicyclus anynana RTE-like non-LTR retrotransposon element
            (BaRTE-d01), partial. Identified  from B. anynana
            microsatellite clones BA-ATG027, BA-ATG101, BA-ATG104, BA-
            ATG220, BA-ATG238, genomic sequence
ACCESSION   .
VERSION     .
KEYWORDS    Microsatellite DNA families, Transposable elements, non-LTR
            RTE, Lepidoptera, microsatellite DNA genesis
SOURCE      Bicyclus anynana
ORGANISM    Bicyclus anynana
            Eukaryota; Metazoa; Arthropoda; Hexapoda; Insecta; Pterygota;
            Neoptera; Endopterygota; Lepidoptera; Glossata; Ditrysia;
            Papilionoidea; Nymphalidae; Satyrinae; Satyrini; Mycalesina;
            Bicyclus.

COMMENT     Identified from DQ225276.1, DQ225278.1, DQ225279.1, DQ225290.1,
            DQ225296.1, DQ225298.1
FEATURES             Location/Qualifiers
     source          .
                     /organism="Bicyclus anynana"
                     /mol_type="genomic DNA"
     5'TSD           .
     5'UTR           .
     CDS             .
                     /codon_start=1
                     /product="reverse transcriptase, partial"
                     /translation="KLPARGSPCKRHFVWKSMQEVYVQQWTSIG***"
     STOP            .
     3'UTR           .
     (TGA)_site      .
     3'TSD           .
```

```
LOCUS       BaRTE-d02     22 bp    mRNA                    26-Sept-2008

DEFINITION  Bicyclus anynana RTE-like non-LTR retrotransposon element
            (BaRTE-d02), partial. Identified  from B. anynana
            microsatellite clones BA-ATG230, genomic sequence
ACCESSION   .
VERSION     .
KEYWORDS    Microsatellite DNA families, Transposable elements, non-LTR
            RTE, Lepidoptera, microsatellite DNA genesis
SOURCE      Bicyclus anynana
ORGANISM    Bicyclus anynana
            Eukaryota; Metazoa; Arthropoda; Hexapoda; Insecta; Pterygota;
            Neoptera; Endopterygota; Lepidoptera; Glossata; Ditrysia;
            Papilionoidea; Nymphalidae; Satyrinae; Satyrini; Mycalesina;
            Bicyclus.

COMMENT     Identified from DQ225294.1
FEATURES             Location/Qualifiers
     source          .
                     /organism="Bicyclus anynana"
                     /mol_type="genomic DNA"
     5'TSD           .
     5'UTR           .
     CDS             .
                     /codon_start=1
                     /product="reverse transcriptase, partial"
                     /translation="RAVWESLQNAYVLQWTSIG***"
     STOP            .
     3'UTR           .
     (TGA)_site      .
     3'TSD           .
```

```
LOCUS       BaRTE-d03     24 bp    mRNA                    26-Sept-2008

DEFINITION  Bicyclus anynana RTE-like non-LTR retrotransposon element
            (BaRTE-d03), partial. Identified  from B. anynana
            microsatellite clones BA-ATG208, BA-ATG216, BA-ATG217, BA-
            ATG223, BA-ATG226, BA-ATG228, BA-ATG237
ACCESSION   .
VERSION     .
KEYWORDS    Microsatellite DNA families, Transposable elements, non-LTR
            RTE, Lepidoptera, microsatellite DNA genesis
SOURCE      Bicyclus anynana
ORGANISM    Bicyclus anynana
            Eukaryota; Metazoa; Arthropoda; Hexapoda; Insecta; Pterygota;
            Neoptera; Endopterygota; Lepidoptera; Glossata; Ditrysia;
            Papilionoidea; Nymphalidae; Satyrinae; Satyrini; Mycalesina;
            Bicyclus.

COMMENT     Identified from DQ225284.1, DQ225288.1, DQ225289.1, DQ225291.1,
            DQ225292.1, DQ225293.1, DQ225295.1
FEATURES             Location/Qualifiers
     source          .
                     /organism="Bicyclus anynana"
                     /mol_type="genomic DNA"
     5'TSD           .
     5'UTR           .
     CDS             .
                     /codon_start=1
                     /product="reverse transcriptase, partial"
                     /translation="KLPNPSHSKRRLELNSESNMC***"
     STOP            .
     3'UTR           .
     (TGA)_site      .
     3'TSD           .
```

```
LOCUS       BaRTE-d04     59 bp    mRNA                    26-Sept-2008

DEFINITION  Bicyclus anynana RTE-like non-LTR retrotransposon element
            (BaRTE-d04), partial. Identified  from B. anynana
            microsatellite clone BA-CA14
ACCESSION   .
VERSION     .
KEYWORDS    Microsatellite DNA families, Transposable elements, non-LTR
            RTE, Lepidoptera, microsatellite DNA genesis
SOURCE      Bicyclus anynana
ORGANISM    Bicyclus anynana
            Eukaryota; Metazoa; Arthropoda; Hexapoda; Insecta; Pterygota;
            Neoptera; Endopterygota; Lepidoptera; Glossata; Ditrysia;
            Papilionoidea; Nymphalidae; Satyrinae; Satyrini; Mycalesina;
            Bicyclus.

COMMENT     Identified from AY785078.1
FEATURES             Location/Qualifiers
     source          .
                     /organism="Bicyclus anynana"
                     /mol_type="genomic DNA"
     5'TSD           .
     5'UTR           .
     CDS             .
                     /codon_start=1
                     /product="reverse transcriptase, partial"

/translation="RPRTGKRSVGRPPTSWTEDIKRVAGSRWMLAARLRCAWKFMQEVYVQQWTAIG*KYNNN"
     STOP            .
     3'UTR           .
     (TGA)_site      .
     3'TSD           .
```

```
LOCUS       BaRTE-d05     58 bp    mRNA                    26-Sept-2008

DEFINITION  Bicyclus anynana RTE-like non-LTR retrotransposon element
            (BaRTE-d05), partial. Identified  from B. anynana
            microsatellite clone BA-ATG212
ACCESSION   .
VERSION     .
KEYWORDS    Microsatellite DNA families, Transposable elements, non-LTR
            RTE, Lepidoptera, microsatellite DNA genesis
SOURCE      Bicyclus anynana
ORGANISM    Bicyclus anynana
            Eukaryota; Metazoa; Arthropoda; Hexapoda; Insecta; Pterygota;
            Neoptera; Endopterygota; Lepidoptera; Glossata; Ditrysia;
            Papilionoidea; Nymphalidae; Satyrinae; Satyrini; Mycalesina;
            Bicyclus.

COMMENT     Identified from DQ225285.1
FEATURES             Location/Qualifiers
     source          .
                     /organism="Bicyclus anynana"
                     /mol_type="genomic DNA"
     5'TSD           .
     5'UTR           .
     CDS             .
                     /codon_start=1
                     /product="reverse transcriptase, partial"

/translation="RPQTRKHSVGRPHPRWTDDIK*VGGIRWMQMAQDRDVWKSLQKVHVPQSQSQ*YDDDD"
     STOP            .
     3'UTR           .
     (TGA)_site      .
     3'TSD           .
```

```
LOCUS       BaRTE-d06     16 bp    mRNA                      26-Sept-2008

DEFINITION  Bicyclus anynana RTE-like non-LTR retrotransposon element
            (BaRTE-d06), partial. Identified  from B. anynana
            microsatellite clones BA-ATG1 and BA-ATG244
ACCESSION   .
VERSION     .
KEYWORDS    Microsatellite DNA families, Transposable elements, non-LTR
            RTE, Lepidoptera, microsatellite DNA genesis
SOURCE      Bicyclus anynana
ORGANISM    Bicyclus anynana
            Eukaryota; Metazoa; Arthropoda; Hexapoda; Insecta; Pterygota;
            Neoptera; Endopterygota; Lepidoptera; Glossata; Ditrysia;
            Papilionoidea; Nymphalidae; Satyrinae; Satyrini; Mycalesina;
            Bicyclus.

COMMENT     Identified from AY785062.1, DQ225299.1
FEATURES             Location/Qualifiers
     source          .
                     /organism="Bicyclus anynana"
                     /mol_type="genomic DNA"
     5'TSD           .
     5'UTR           .
     CDS             .
                     /codon_start=1
                     /product="reverse transcriptase, partial"
                     /translation="ASYVLQWTSIG*YDDD"
     STOP            .
     3'UTR           .
     (TGA)_site      .
     3'TSD           .
```
