# Supplementary material for: Generation of microsatellite repeat families by RTE retrotransposons in lepidopteran genomes
Source: BMC Evol Biol. 2010 May 17;10:144. doi: 10.1186/1471-2148-10-144 (PMC2887409; doi:10.1186/1471-2148-10-144)
Supplement: Additional file 4 — Examples of partial RTE elements within lepidopteran protein encoding DNA sequences deposited in GenBank. Target site duplication (TSD) sequences are identified and provided where possible, as were associated simple sequence repeat (SSR) units that flanked the 3' UTR of these RTEs. Protein sequence identity (% identity) and E-value to BmRTEs and partial lepidopteran RTEs (Additional File 3) were determined by stand-alone blastx search. Missing SSR units or unidentified TSD are indicated by '?'. Nucleotide position indicates the location where the RTE is inserted in the host genome, and the length (in bp) of the partial RTE identified are also provided. [file 1471-2148-10-144-S4.PDF]

Additional File 4 (.pdf): Examples of partial RTE elements within lepidopteran protein encoding DNA sequences deposited in GenBank.

Target site duplication (TSD) sequences are identified and provided where possible, as were associated simple sequence repeat (SSR) units that flanked the 3' UTR of these RTEs. Protein sequence identity (% identity) and E-value to BmRTEs and partial lepidopteran RTEs (Additional File 3) were determined by stand-alone blastx search. Missing SSR units or unidentified TSD are indicated by '?'. Nucleotide position indicates the location where the RTE is inserted in the host genome, and the length (in bp) of the partial RTE identified are also provided.

Accession Number	RTE	% Identity	E Value	Length (bp)	Nucleotide position	SSR units	TSD	Associated gene(s)	Host	Reference
DQ788839	HzRTE-1	78%*	0 .0*	1,754	891..2,655	(TGA)$_7$	TTAAGTTAGA	CYP9A12v3	*Helicoverpa zea*	[21]
EF113398	ezi-D11alpha-1	78%†	0.0†	1,437	863..2,299	?	?	desaturase	*Ostrinia nubilalis*	[22]
AY714875<br>AY714876	HaRTE-t01	95%	7e-12	90	173..262	(TGR)$_{10}$	AGGTATC	E-cadherin	*H. armigera*	This study
U46130	HzRTE-1-like	48%†	0.001†	224	908..1,1,31	(TGA)$_5$	TATGTAYYA	Preproattacin A	*Trichoplusia ni*	This study
AB262389	BmRTE-d02	88%	9e-84	521	59,634...60,154	(TGA)$_5$	ATCGCCATTTTG	BMWCP5, 4, 3, 2	*Bombyx mori*	This study
	BmRTE-d09	97%	3e-82	442	89,594..90,035	(TGA)$_6$	TTAAAGTTGTC	BMWCP5, 4, 3, 2	*B. mori*	This study
AY789465	BmRTE-d02	77%	6e-25	214	5,633..5,846	(GAT)$_6$	TAATA	PAP-1	*Manduca sexta*	This study
AB288052	BmRTE-d08	76%	1e-06	66	1,870..1,935	?	?	Ricini-SP2	*Samia cynthia*	This study
AY962308	BaRTE-d06	90%	0.001	81	5,679..5,759	(KAW)$_5$	?	FPPS-1	*Choristoneura fumiferana*	This study
M73793	BmRTE-d08	76%	0.014	58	552..609	(TGA)AA(YGA)$_4$	TTATGGCA	Arylphorin	*Galleria mellonella*	This study
CU655868	BmRTE-d01	71%	5e-40	312	70,544..70,855	?	?	?[a]	*Heliconius numata*	This study
AY172028	BmRTE-d01	63%	9e-13	135	1,502..1,636	(YGA)$_6$	?	Larval serum protein	*B. mandarina*	This study

Note: BMWCP5, 4, 3, 2 (*Bombyx mori* BMWCP5, BMWCP4, BMWCP3, BMWCP2 genes for cuticle); PAP-1 (prophenoloxidase-activating proteinase-1); FPPS-1 (farnesyl diphosphate synthase); ?[a]. unannotated *Heliconius numata* BAC clone AEHN-31F4. Amino acids sequence identity (% identity) and E-value to BmRTE-d01 and BmRTE-d08 are indicated by '*' and '†' respectively. Ambiguous nucleotides are: K (G/T), W (A/T), Y (C/T).
