# Supplementary material for: Generation of microsatellite repeat families by RTE retrotransposons in lepidopteran genomes
Source: BMC Evol Biol. 2010 May 17;10:144. doi: 10.1186/1471-2148-10-144 (PMC2887409; doi:10.1186/1471-2148-10-144)
Supplement: Additional file 6 — Table showing the top 16 lepidopteran species with the most abundant microsatellite DNA sequences deposited in GenBank (30 June, 2009). Using as input queries lepidopteran RTE amino acid sequences in Additional Files 3, 3, and 8 (for tblastn), and the DNA sequences of partial RTEs in Additional File 3 (for blastn), BLAST searches within GenBank against the entrez query search term "Lepidoptera Microsatellite" were carried out. Percentages in parentheses indicate proportion of microsatellite DNA sequences that contained RTEs. The overall proportion of lepidopteran microsatellite DNA sequences containing RTEs remained similar at 9.26% when Bombyx mori microsatellite DNA sequences were excluded (due to the majority of these microsatellite DNA sequences being reported for primer regions instead of the actual microsatellite simple sequence repeat unit regions). The 68 B. mori microsatellite DNA loci identified to be associated with RTE elements included 64 from 518 loci deposited in GenBank by Miao et al. [27]. Most of these loci are represented by separate forward and reverse sequence reads, so there are about twice as many sequences deposited as numbers of loci. Among the 16 Lepidoptera species with the most abundant microsatellite DNA sequences in GenBank, RTE content ranged from 0% in Comonympha hero to 28.75% in Helicoverpa armigera, 30.0% in Busseola fusca and 43.55% in Bicyclus anynana. [file 1471-2148-10-144-S6.PDF]

Additional File 6 (.pdf): Table showing the top 16 lepidopteran species with the most abundant microsatellite DNA sequences deposited in GenBank (30 June, 2009).

Using as input queries lepidopteran RTE amino acid sequences in Additional Files 4, 5, and 8 (for tblastn), and the DNA sequences of partial RTEs in Additional File 5 (for blastn), BLAST searches within GenBank against the entrez query search term "Lepidoptera Microsatellite" were carried out. Percentages in parentheses indicate proportion of microsatellite DNA sequences that contained RTEs. The overall proportion of lepidopteran microsatellite DNA sequences containing RTEs remained similar at 9.26% when *Bombyx mori* microsatellite DNA sequences were excluded (due to the majority of these microsatellite DNA sequences being reported for primer regions instead of the actual microsatellite simple sequence repeat unit regions). The 68 *B. mori* microsatellite DNA loci identified to be associated with RTE elements included 64 from 518 loci deposited in GenBank by Miao et al. [27]. Most of these loci are represented by separate forward and reverse sequence reads, so there are about twice as many sequences deposited as numbers of loci. Among the 16 Lepidoptera species with the most abundant microsatellite DNA sequences in GenBank, RTE content ranged from 0% in *Comonympha hero* to 28.75% in *Helicoverpa armigera*, 30.0% in *Busseola fusca* and 43.55% in *Bicyclus anynana*.

**Species**	**Microsatellite DNA sequences in GenBank**	**RTE found in microsatellite DNA sequences**	
Bombyx mori	564	68	(12.1%)
Cydia pomonella	193	16	(8.29%)
Plutella xylostella	145	6	(4.14%)
Arctia caja	103	10	(9.71%)
Parnassius apollo	83	1	(1.20%)
Drupadia theda	82	3	(3.66%)
Euphydryas aurinia	79	10	(12.66%)
Melitaea cinxia	72	20	(27.78%)
Busseola fusca	70	21	(30.00%)
Ostrinia nubilalis	66	2	(3.03%)
Bicyclus anynana	62	27	(43.55%)
Arhopala epimuta	60	8	(13.33%)
Coenonympha hero	45	0	(0.00%)
Parnassius mnemosyne	36	1	(2.78%)
Parnassius smintheus	35	1	(2.78%)
Helicoverpa armigera	28	8	(28.75%)
All other taxa	460	16	(3.48%)
**Total**	2,183	218	(9.98%)
