# Supplementary material for: Generation of microsatellite repeat families by RTE retrotransposons in lepidopteran genomes
Source: BMC Evol Biol. 2010 May 17;10:144. doi: 10.1186/1471-2148-10-144 (PMC2887409; doi:10.1186/1471-2148-10-144)
Supplement: Additional file 7 — Amino acid alignment of non-LTR retrotransposable element reverse transcriptase (RT) conserved domains by Kalign in EMBL-EBI used to infer phylogenetic relationships between different clades of non-LTR TEs. Alignment of the Reverse Transcriptase (RT) conserved domain from selected non-LTR retroelements of Malik et al. [23] and Novikova et al. [24], as well as the RT conserved domain of full-length RTE elements from Bombyx mori and Heliconius melpomene identified from this study. BmRTE-d24 and BmRTE-d25 are new full-length B. mori RTE elements that include the partial sequences previously identified by Zupunski et al. [22]. Sequence alignment used default settings of the Kalign program [51,52] in EMBL-EBI. Gaps inserted for alignment purposes are indicated by '-'. [file 1471-2148-10-144-S7.PDF]

Additional File 7 (.pdf): Amino acid alignment of non-LTR retrotransposable element reverse transcriptase (RT) conserved domains by Kalign in EMBL-EBI used to infer phylogenetic relationships between different clades of non-LTR TEs.

Alignment of the Reverse Transcriptase (RT) conserved domain from selected non-LTR retroelements of Malik et al. [23] and Novikova et al. [24], as well as the RT conserved domain of full-length RTE elements from *Bombyx mori* and *Heliconius melpomene* identified from this study. BmRTE-d24 and BmRTE-d25 are new full-length *B. mori* RTE elements that include the partial sequences previously identified by Zupunski et al. [22]. Sequence alignment used default settings of the Kalign program [51, 52] in EMBL-EBI. Gaps inserted for alignment purposes are indicated by '-'.

```
BmRTE-d01      RSIVVLFFKKG-D-KTLLK--NYRPISLLSHVYKLFSRVITNRLAQR---LD--DFQPP
BmRTE-d02      RGVVILFFKKG-D-KTLLK--NYRPIALLSHVYKLFSRVITKRLERR---FD--DFQPT
BmRTE-d03      RGVVVLFFKKG-D-KSLLK--NYRPITLLSHVYKLCSRVITNRLERR---LD--DFQPI
BmRTE-d04      RSRVVLFFKKG-D-KTLLK--NYRPISLLSHVYKVFSRVITNRLARK---LD--ECQPP
BmRTE-d05      ESLLIPLHKKG-S-TRTCS--NYRTVALISHASKVMLYVINGRLQSY---IQ--WQIPS
BmRTE-d06      HSIFIPLHKKG-S-TKKCN--NYRLISLVSHASKVMLHIINTRLQGY---LS--REIAP
BmRTE-d07      KSIILPLHKKG-S-TRKCD--NYRTLALVSHCSKVLLYILNTRLRYF---LD--WQIPQ
BmRTE-d08      KSVVVLFFKKG-D-NTRLK--NYRPISLLSHVYKLFSRVITNRLARR---FD--DFQPP
BmRTE-d09      -SIVILFFKKG-D-KTLLK--NYRPISLLNHIYKLFSRVITNRLARR---FD--EFQPP
BmRTE-d10      KGTVILFFKKG-D-RALLK--NYRPISLLSHIYKLFSRVLTNRLASR---LD--EFQPP
BmRTE-d11      HTVFVPLHKKG-S-TKSCS--NYRLIALIPHACKILLRIVNERLKSY---LS--KEIAP
BmRTE-d12      ------LHKKG-S-TKDCN--NYRTLALISHASKILLHIINSRIRHF---LD--WQIPQ
BmRTE-d13      STIVPIYKgKG-S-KYECS--NYRGIKLLSHTMKLYERVIDSRLRS-----E--CSLSK
BmRTE-d14      TAVICPLHKKG-D-VLDCQ--NYRGISLLNTAYKIFANVLFGKLKPF---VE--PRLGE
BmRTE-d15      SRITALYKnKG-D-RSDCN--SYRGISLLSAPGKVFARVLLNRLKNL---SE--KILPE
BmRTE-d16      HSAILPLHKKG-T-TRRCD--NYRTIALISHTSKVLLHVINSRLRHF---LD--WQIPQ
BmRTE-d17      -GLLVKVPKKG-D-LSSCD--NWKGITLLPCAAKVLARLLLNRMSKK---MT--GTLRE
BmRTE-d18      ESILIPLHKKG-S-TRKCE--NYRTISIISHASKILLHIINKRLESF---IS--RQIAK
BmRTE-d19      HTVFTPLHKKG-S-TKKCN--NYRLIALTSHSSKIMLHILNERLKTY---LS--KEIAP
BmRTE-d20      TSTIILIHKKG-R-TDDIS--NYRPISLMSNIYKLFSKIVLERLTRT---LD--ENQPK
BmRTE-d21      ESNMILLYKKG-D-PTDIG--NYRPISLLPTLYKLFSSIIEKRISKR---IE--EHQPT
BmRTE-d22      ESIIILLYKKG-N-PNDIS--NYRPISLLATVYKLFSSIINRRISET---LE--KRQPI
BmRTE-d23      ECHIILLHKKG-T-RDEIG--NYRPISLISNIYKVFAKVILERISLT---LN--ESQPA
HmRTE-e01      SFITPIYKGrG-S-VQDCG--SYRGIKLMSHTMKLFERMIDLRLRR-----E--CTVSE
Slacs          ATSLTVLRKPN-------G--KYRPIGAESVWAKLASHIAISRVMKT---AE--KKFSG
CZAR           ATNLTVLRKPN-------K--KFPPIGAECVWAKAISLMAVDAVMPA---LK--TCFKN
CRE-1          SAVTPIPKDEA-------GT-KIRPIVPESAWLKLASLVAMAEIPSS---FK--ETFKG
CRE-2          ACILHPFRKEA-------GSAKVRPITPESALMKLAAHIALDSVEKS---FR--STFKG
R2-Drosmel     -ARTVFIPKTV-T-AKRPQ--DFRPISVPSVLVRQLNAILATRLNSS---IN----WDP
R2-Drossim     -ARTVFIPKTV-T-AKRPQ--DFRPISVPSVLVRQLNAILATRLNSS---IN----WDP
R2-Drosyak     ESKTTLIPKKP-N-STEPG--DFRPITVQSVLVRQLNKILAARVAQH---IP----LDE
R2-Drosmer     -ARTIFIPKTV-R-ANRPQ--DFRPISVPSIVVRQLNAILASRLTAA---VS----WDP
R2-Earwig      ESKTTLIPKKP-N-STEPG--DFRPITVQSVLVRQLNKILAARVAQH---IP----LDE
R2-Bmori       -CRTVFVPKVE-R-PGGPG--EYRPISIASIPLRHFHSILARRLLAC---CP----PDA
R2-Isopod      KGRITLIPKKS-L-PELAG--DFRPICVLPVVVRLLHRILAKRLAIV---QH--TEF--
Dong           TGITYMIPKGD-F-SIEAS--QYRPITCLPTIYKILTTVITKKINSH---IEHNNIlaE
```

```
R4             RGRTILIPKKG-D-RGDPS--NYRPITCLNTCYKVLTSVMNSVILSH---LSrgEALPM
L1-Rat         EATITLIPKPhKD-TTKKE--NFRPISLMNINAKILNKILANRIQEH---IK--TIIHH
L1-Huamn       EASIILIPKPGRD-TTKKe--NFRPISLMNIDAKILNKILA-KIQQH---IK--KFIHR
L1-Mouse       EATITLIPKPqKD-PTKIE--NFRPISLMNIDAKILNKILANRIQEH---IK--AIIHP
L1-Dog         EASITLIPKPDKD-PTKKe--NYRPISLMNMDAKILNKILANRIQQY---IK--KIIHH
SwimmerMedaka  KAIISIIPKEGKD-RlDCA--NYRPVSVLNIDYKLFTSIISRRLETI---LP--MLIHK
SwimmerPupfis  EAIISIIPKENrD-KLECS--NYRPISVLNIDYKLFTSILSKRLEII---LP--DLINK
Tx1            RAVLSLLPKKG-D-LRLIK--NWRPVSLLSTDYKIVAKAISLRLKSV---LA--EVIHP
Cin4           EANIVLLPKRE-N-PDRID--LFRPISLINSCMKIITKIMATRLAPR---MN--EIVST
Ta11           -----------------D--L-RPISLCSVLYKIISKIMVRRLQPF---LP--DLVSP
DRE            GILITIYKnKG-D-PNNLD--NYRPITLLNVDYKIYSKIINNRILKL---LN--KIISP
Zepp           GRITLLYKGKgaD-RESLA--SYRPITLLNTDYKLAARAIASRIGPL---LN--QVVDA
BDDF            RSVFIPIPKKG-N-AKECS--NYRTIALISHASKVMLKVLQARLQQY---VN--RELPD
JAM1            -GVICPIYKKG-D-KLECE--NYRAITILNAAYKVLSQILFRRLLPI---AN--EFVGS
RTE-1           TSKTTLIFKKG-D-RENLE--NYRPICLLPVLYKVFTKCLLNRMRRS---LD--EAQP-
RTE-2           ---VKLIPKKA-K-ATKIK--DFRPISLLPILSKMFSSILTRRLTPT---LE--SYLDE
CR1-chicken     KGYVTPIYKKG-S-KEDPG--NYRPVSLTSVPGKIMEQILLDDMLDH-MrnE--RVIRD
Q               ----VPIYKKG-D-RTDAI--NYRGITSLCAIAKVFELVIYKNLLH---ACR--SYLSP
T1              -----PVHKKG-C-RSIVS--NYRGITQTCATAKTFELCIFPTILH---SCS--SAISP
CR1-Turtle      KANIVPIFKKG-K-KEDPG--NYRPVSLTSXPGKIMEQVLKESILRH-LEER--kvIRX
SR1             -AHITPIFKGG-R-RSEPS--SYRPVALLSIPSKIMESLIYDGILEY-LSss--KFFSP
Sam6            ESTVVPIFKKG-N-ASDPE--NYRPISLTHPLSRLFEKLVLQSIKK---TCA--NKLSK
Sam3            -SIIVPVHKKG-C-RTDAN--NYRPISLTHPLSRVFEKFIVEKLRK---ECS--SKISK
BmCR1-Bombyx    TALVHPIPKKG-D-RSDPS--NYRPIAITSLLSKIMESIINRQLMGY-LEEH--qlISD
Jocky-Drosmel   -ASIIMIHKTG-KtPTDVD--SYRPTSLLPSLGKIMERLILNRLLtc-KDVT--KAIPK
Jocky-Dfunebr   TASIIMILKPG-KqPLDVD--SYRPTSLLPSLGKMLERLILNRILTS-eeVT--RAIPK
TART            HAQVKMILKPG-KsANEPR--SYRPISLLSGLSKIFERLLLKRLFKV-dlFK--KAIPL
G               RAEVITIPKPG-KPEaNLA--SYRPISLLAILSKILERVFLRRVLPV-LDEa--gLIPD
Doc             KSTIVMIPKPG-KDKTqPS--SYRPISLLTCLSKLFEKMLLLRISPH-LRIN--NtlPT
F               --KIIMIPKPG-KNHTVaS--SYRPISLLSCISKLFEKCLLIRLNQHqttYH--NIIPA
YAKPs1          -AKILMVHKPG-KPlEDPK--SYRPISLLPVASKVYESLLLLRLLPI-IKEK--KliPD
BS              -AAILMIHKPG-KPEeSPE--SYRPISLLSSLSKLWERLIANRLNDI-MTER--riLPD
Helena-Dyak     -AVIMMIHKPG-KPEaDPE--SYRPISLLPSLSKLWERLLVKRINDI-VRQG--NIlpD
Helena-Dmau     -AAILMIHKPG-KPeDDPE--SYRPISLLPSLSKLWERLIANRINDI-IRQG--NIlpD
AMY             EADVIGIHKPG-KPkNDPT--SYRPISLLMSLGKLYERLLYKRLRDF-VSSk--gILID
JuanA           -AKVVPILKPD-KnPAEAS--SYRPISLLSSISKLFEKVILNRMMAH-INEN--SIfaN
JuanC           -AKVIPILKPD-KnPAEAS--SYRPISLLSSISKLFERIILNRMMTH-INEN--SIfaD
NCR1Cth         HAKVIPIPKPG-KPTNeVS--SYRPISLVSSISKILERILLNRINDH-LEDN--niIPN
BmRTE-d24       KSTFIKIPKKQ-N-AKKCG--EYRMISLMSHVLKVFLNIIQNRIRPK---CD--EQLGD
BmRTE-d25       KSAIVPLHKKG-S-TAKCE--NYRTLSLMSHASKILLRIINSRLSAF---ID--HQIPR

BmRTE-d01      RQAGFRRGF-GTVDHIHTVRQ--IIQKTE-----EYNL--P-L---CLAFVDYEKAFDSI
BmRTE-d02      RQAGFRKGY-STIDHIHTLRQ--IVQKTE-----EYNR--P-L---CLAFVDYEKAFDSV
BmRTE-d03      RQAGFRKGF-STIDHIHTLRQ--IIQKTE-----EYNR--P-L---CLAFVDYEKAFDSV
BmRTE-d04      RQAGFRKGY-NTVDHIHTLRQ--IIEKST-----EYNL--P-L---CVAFVDYEKAFDSI
BmRTE-d05      EQAGFVKGR-GTREQIVNVRQ--IIEKSR-----EFNM--P-I---LLCFIDYTKAFDCV
BmRTE-d06      HQAGFVKGR-GTREQLLVMRQ--IVEKAR-----EFNI--S-L---YVCFVDFRKAFDTV
BmRTE-d07      KQAGFVKGK-GTREQILNIRQ--LIERSY-----EFGT--P-M---IMCFIDYSKAFDCV
BmRTE-d08      KQAGFRKGY-STVDHIHTLRQ--IVQKTE-----EYNL--P-L---CLAFVDYEKAFDSI
BmRTE-d09      -QAGFRSGY-GTIDHIHTVRQ--IIEKTT-----EYNR--P-L---CLAFVDYEKAFDSi
BmRTE-d10      KQAGFRKGY-STVDHIHTLRQ--VIQKIE-----EYNR--P-L---CLAFVDYEKAFDSV
BmRTE-d11      HQAGFVKGK-GTREQILTVRQ--IIEKSR-----EFNK--P-T---YLCFVDFSKAFDSV
BmRTE-d12      -QAGFVKGR-GTREQILNIRQ--LIERCH-----EFDT--P-I---ILCFVDYSKAFDCV
BmRTE-d13      SHYGFVQGL-STTDPMFALNT--IAEEYR-----EKLR--P-L---YVAFLDMEKAFDRV
BmRTE-d14      TQCGFRPGR-STIDQIFSLRQ--ILEKTL-----EFNA--D-T---YHLFIDFKAAYDNI
BmRTE-d15      SQFGFRPDR-GTCEAIFSVRQ--LQEKSR-----EQGR--Q-L---YLCFVDLEKAFDSV
BmRTE-d16      HQAGFVKGK-GTREQILNVRL--IIETCY-----EYNI--P-A---VLCFVDYQKAFDCV
BmRTE-d17      -QAGFLPGR-SCTDHTNTLRI--LIEQSV-----EWQT--E-M---ILTFVDFEKAFDTV
BmRTE-d18      EQAGFVKGR-GTREQILNIRQ--LIEKAR-----EFNV--P-M---ALCFIDYAKAFDCI
BmRTE-d19      HQAGFVRGK-GTREQIFIVRQ--IIEKAR-----EFNR--P-T---YICFVDFSKAFDSV
BmRTE-d20      TQAGFRSGF-STLDHIHTIKQ--IIQKCN-----EYNI--N-Y---YLSFIDYNKAFDSL
BmRTE-d21      EQAGFRRGY-STIDHIHAIEQ--IVEKYN-----EYQK--P-L---YVVFIDYRKAFDTI
BmRTE-d22      EQAGFRKGY-STVDHIHTLEL--IIEKYQ-----EKQR--P-L---YIAFIDYQKAFDTI
```

```
BmRTE-d23      EQAGFRKGF-STIDHIHTIKQ--LIQKYN-----EYNK--Q-I---YLAFIDYSKAFDSL
HmRTE-e01      SQYGFQPGS-GTMDAIFALRT--LMEAYR-----EKRR--A-L---HVAFLDLQKAFDCV
Slacs          AQFGVGGHI-E--------EA--IAKIRK-----DFAT--K-G---SLAMLDGRNAYNAI
CZAR           AQYGVGNNI-E--------LA--IQKIRR-----DFHL--K-G---SVAMLDGRNAYNAI
CRE-1          SQYGVWGDV-A--------KA--VAKIRR-----DSEE--H-E---YLVALDGVNAYNTM
CRE-2          AQYGVWGDS-T--------EA--VKRIRE-----AYAEASS-D---TLVALDATNAYNRM
R2-Drosmel     -QRGFLPTD-GCADNATIVDL--VLRHSH-----KHFR--S-C---YIANLDVSKAFDSL
R2-Drossim     -QRGFLPTD-GCAYNATMVDL--VLRHSH-----KHFR--S-C---YIANLDVSKAFDSL
R2-Drosyak     EQRGFRPVD-GVAHNIFELDM--ILRCHR-----SEFR--D-L---RLASLDIAKAFDSI
R2-Drosmer     -QRGFLPTD-GCADNATIVDL--VLRDHH-----KRYA--S-C---YIATLDVSKAFDSV
R2-Earwig      EQRGFRPVD-GVAHNIFELDM--ILRCHR-----SEFR--D-L---RLASLDIAKAFDSI
R2-Bmori       -QRGFICAD-GTLENSAVLDA--VLGDSR-----KKLR--E-C---HVAVLDFAKAFDTV
R2-Isopod      KQAGFQSGR-STSENIFLLRT--ILESLP-----AGKE--S-M---YIALLDFRKAFDSV
Dong           TQKGCRRGHmGCKEQLIIDST--IMKHAT-----TKNR--N-L---HCTYIDYKKAFDSI
R4             RQRAMRKRE-WGCTHAMVLDRamVMDAMA-----QKKH--S-L---SVAWLDYRKAYDSV
L1-Rat         EQVGFIPGM-QGWFNIRKTIN--VIHYIN-----KLKe--Q-NH--MIISLDAEKAFDKI
L1-Huamn       EQVGFIPGM-QDWFNMHKSIN--VIQHIN-----RTKd--K-NH--MIVSIDAEKAFDKI
L1-Mouse       EQVGFIPGM-QGWFNIRKSIN--VIHYIN-----KLKd--K-NH--MIISLDAEKAFDKI
L1-Dog         EQVGFIPGT-QGWFNTRKTIN--VIHHIS-----KRKt--K-NH--MILSLDAEKAFDKI
SwimmerMedaka  KQTGFIKQR-QTQDSIRKVLH--IIHQVV-----QQKQ--E-T---LVISLDAEKAFDSV
SwimmerPupfis  EQTGFIRQR-QTQDNIRKTLH--IMKYVS-----QHKL--E-T---LILSLDAEKAFDSV
Tx1            RQSYTVPGR-TIFDNVFLIRD--LLHFAR-----RTGL--S-L---AFLSLDQEKAFDRV
Cin4           EQNAFIQKR-SIHDNFLYVQK--VIKKLH-----KSKQ--A-A---LFVKLDISKAFDSL
Ta11           -QSAFVAER-LIFDNILIAHE--VVHGLR-----THKs--v-sKGFIAIKSNMSKAFDRV
DRE            GQTGFVPRR-LLHDNIITLNS--TIEIIKReintkEDM--E-P---IITFYDFEKAFDSI
Zepp           GQTGFLPKR-WAGDNVLAHLE--EISYLE-----ATHQ--P-G---VQVFLDFEKAFDRL
BDDF            QAGFRKGR-GTRDQIANICW--IMEKAR-----EFQK--N-I---YFCFIDYAKAFDCV
JAM1            QTGFIDGR-STMYWIFSVRQ--ILQKCR-----EYQV--P-T---HHLFIDFKAAYDSI
RTE-1           QAGFRRSF-STIDHIHSLQR--LLEVGR-----EYQI--P-L---TLVFIDFKKAFDSV
RTE-2           QNGFRKGR-CCADNIQSLTM--LIEKCN-----EFQL--P-L---LLLFIDYQTAFDKI
CR1-chicken     QHGFTRGR-SCLTNLVAFYD--GVTALV-----DEGK--A-T---DVIYLDLTKAFDMV
Q               QHGFVPKK-STTTNLVEFVT--YCTSQI-----DAGA--Q-V---DAIYTDLKAAFDSL
T1              QHGFMPGR-STSTNLMSFVT--NIFRSF-----EAGT--Q-L---DAIYTDFHAAFDSL
CR1-Turtle      QHGFTKGK-SCLTNLIAFYE--EVTGSV-----DVGK--A-V---DVIYLDFSKAFDTV
SR1             QHGFRKGH-SCMTNLLTAVD--RWTTIL-----DRKG--K-V---DVIYLDFSKAFDRV
Sam6            QFGFLNNR-SCTLAHLNSST--FCHSIL-----SHpR--KFL---DIVLFDFRKAFDTV
Sam3            QFGFMNSR-SCTLALLNACS--KILDSL-----TiRS--KYV---DAIYLDFKKAFDSV
BmCR1-Bombyx    QYGFRKGR-SAGDLLALLTH--RWAQAV-----ESRG--E-A---LGVSLDIAKAFDRV
Jocky-Drosmel   QFGFRLQH-GTPEQLHRVVN--FALEAM-----ENKE--Y-A---VGAFLDIQQAFDRV
Jocky-Dfunebr   QFGFRLQH-GTPEQLHRVVN--FALEAL-----EKKE--Y-A---GSCFLDIQQAFDRV
TART            QFGFRKEH-GSEQQIARVTQ--FILEAF-----ERKE--Y-C---SAVFLDISEAFDRV
G               QFGFRRSH-GTPEQCHRLVE--QILEAF-----ERKQ--Y-C---CAVMLDVKQAFDKV
Doc             QFGFREKH-GTIEQVNRITS--EIRTAF-----EHRE--Y-C---TAIFLDVAQAFDRV
F               QFGFRESH-GTIEQVNRITT--EIRTAF-----EYRE--Y-C---TAVFLDVSQAFDKV
YAKPs1          QFGFRQKH-GTIDQVHRLVS--KIHNTF-----EDKE--Y-C---AAAFLDISQAFDRV
BS              QFGFRQGH-STVEQVHRLTK--HILQAF-----DDKE--Y-C---NAVFIDMQQAFDRV
Helena-Dyak     QFGFRKGH-GTVEQVHRLVK--HILQAL-----DDCE--Y-S---NAVFIDMQQAFDKV
Helena-Dmau     QFGFRKGH-GTIEQVHRLVK--HILQAF-----DDCE--Y-S---NAVFIDMQQAFDKV
AMY             QFGFRTNH-SCVQQVHRLTE--HILVGL-----NRPKPLY-t---gALFFDVAKAFDKV
JuanA           QFGFRHGH-STTHQLLRVTN--LTRANK-----SEGY--S-T---GLALLDIEKAFDSV
JuanC           QFGFRLGH-STTHQLLRVSN--LIRSNK-----SEGY--S-T---APALLDIEKAFDSV
NCR1Cth         QCGFRTGR-STSHQLIKVIK--TAKENI-----NNKK--S-T---GMIFLDVEKAFDRV
BmRTE-d24       QFGFRSGV-STREALFALNV--LVQKCR-----DMQT--D-V---FLCFIDYEKAFDRV
BmRTE-d25       QTGFVAGK-GTREQILNVRV--LIEKFR-----EFNK--P-L---VLCFIDYAKAFDCV

BmRTE-d01      RiwavleslqrcqaDWRYIdaLRCLy-dtatmtvqvq-k----d---qtr-piqlrrGVR
BmRTE-d02      RtwavlrslqrcriDHRYIevLKCLy-nnatmsvrvq-e----h---ctk-eipvkrGVR
BmRTE-d03      RTWAVLRSLqrcridyryvevlkyly-nnatmsvrvq-e----h---ctk-eitlkrGVR
BmRTE-d04      RtWSVLeflqrcqidyryievLKSLy-eaatmtvqiq-d----c---qsi-piklqrGVR
BmRTE-d05      EWDCLWRILremgvpqhlvsliasly-rdgvsmvrvn-d----v---isg-pfkpekGVR
BmRTE-d06      HWWKLWLVLtemgvpqhlvhtirrly-edgtaavrvd-s----i---dse-rfstqaGVR
BmRTE-d07      KWDHLWKILaelgvpqhlvlllhsly-innqgiirve-e----t---isa-pfkfrkGVR
BmRTE-d08      KTWAMLQSLqrcqidyryievlrcly-enatmsvrvq-d----r---ase-pillqrGVR
```

```
BmRTE-d09     -twsvldslrrcqVDHRYVqvLKCLy-dcatm-msiq-n----q---qsn-piqlrrGVR
BmRTE-d10     KTWAVLESLqrclvdyryvevlksly-kaakmtvqiq-n----q---qtn-pielhrGVR
BmRTE-d11     HWPKLWETVlamgtpkhlvhllrrly-eegtasvrid-d----i---lsr-hfhpnaGVR
BmRTE-d12     -WNCLWRVLqelgvpmhlkaflqsly-ygsqgtvrvd-y----t---msn-rfnfrrGVR
BmRTE-d13     SRDTIWWSLrkknvpehyvnviidmy-rdarsmvrtv-v----g---qtk-piavaeGLH
BmRTE-d14     TRDFLYQAMheigvppklisltrmtl-vasqsivkiq-t----d---lsd-pfrthdGLR
BmRTE-d15     SREALWLVLrklgctekfvallrllh-ddmqccvavd-g----e---qtg-ffpvtcGVK
BmRTE-d16     HWKHLWYVLkdmgvpmhliqlmraly-lsgrgsvrig-p----a---qsr-efrfekGVR
BmRTE-d17     -WSKMWTCLkqrgipnkiigimqaly-rgstcrvvhd-q----v---lga-piemtaGVK
BmRTE-d18     EWKKMFDVMqemgipdhlvslvqtlh-mdgitrvrmd-n----e---fsh-pfkperGVR
BmRTE-d19     HWPVLWKTLldlgtpkhlvhllrrly-engtasvrad-d----v---lsg-nfhpsaGVR
BmRTE-d20     THQKIWEALalqgvhnkyirllkniy-enmkarvrt--e----k---lge-hfhikkGVR
BmRTE-d21     EHSSIWTALisqrvehkyieiikyly-nnctsrvkl--e----t---tgp-pipirrGVR
BmRTE-d22     EHTSIWEALnqqgveseyiqvikniy-knsvskvkl--e----t---igp-dfninrGVR
BmRTE-d23     EHQYIWRSLeqqgvqsnyieilksiy-ksskasikl--e----s---tge-sfpikkGVR
HmRTE-e01     SRQCIWWALrskgipeayieiirgmy-hdsasmvrta-v----g---dtr-pfpitvGVH
Slacs         ARRAILEAVygdstwsplwrlvslllgttgevgfyen-g----k---lch-twestrGVR
CZAR          ARTAILSAVygntawsplwrvtrlllgteglvgfyek-g----q---lvh-swkstrGVR
CRE-1         SRAHILQAVyaeqrlkpiwgvvkvalggpgflgvyrd-g----c---lkg-nlwstkGIR
CRE-2         ARRHILEAAyaqpelrfafgvvnlslgaagelalyen-g----a---kih-alksteGVR
R2-Drosmel    -HASIYDTLraygapkgfvdyvqnty-egggtslngd-g----w---sse-efvparGVK
R2-Drossim    -HASIYDTLraygapkgfvdyaqnty-egggtslngd-g----w---sse-eivparGVK
R2-Drosyak    EHNTIEDTMevrgfpkpminyimacy-rrsktrftfn-g----w---isd-tvkptcGVK
R2-Drosmer    -HDAVFNTVtaygapksfvdyvrrwy-sdggtyfngg-d----w---rse-efvparGVK
R2-Earwig     EHNTIEDTMevrgfpkpminyimacy-rrsktrftfn-g----w---isd-tvkptcGVK
R2-Bmori      -HEALVELLrlrgmpeqfcgyiahly-dtasttlavn-n----e---mss-pvkvgrGVR
R2-Isopod     KHTVLCGLLrdlglperltgyvesiy-rsvhltlg------------dd-wfvqgrGVL
Dong          THSWLIQVLeiykinpiiisflrnim-thwqttlklk-nppnfv---ttr-qiaikkGIY
R4            RHEYIrwainsvniprsvqltLKRLm-sdwetrfest-q--c-rpklrsd-kmkvlnGIF
L1-Rat        EHPFMIKVLerigiqqpylnivkaiy-skpvanikln-g----e---kle-aiplksGTR
L1-Huamn      EQPFMLKTLnklgidgtyfkiiraiy-dkptaniiln-g----q---kle-afplktGTR
L1-Mouse      EHPFMIKVLersgiqqpylnmikaiy-skpvanikvn-g----e---kle-aiplksGTR
L1-Dog        EHPFLIKTLqsvgiegtfldilkaiy-ekptaniiln-g----e---alg-afplrsGTR
SwimmerMedaka KWTFLYKVLgkfgfcksiietisgly-nkptarikin-g----d---fte-titlerGTR
SwimmerPupfis EWAFLYKVLskfgfhpnivdtfaaly-skptakikvn-g----d---ltn-sftlqrGSR
Tx1           RHQYLIGTLqaysfgpqfvgylktmy-asaeclvkin-w----s---lta-plafgrGVR
Cin4          EWAYLLDVLkalgftqkwrdwiatil-gsssskiiin-g----q---qtk-eikhmrGVR
Ta11          -WNYvraLLDALgfhqkwvgwimfmi-ssvsysvlin-d----k---afg-nivpsrGLR
DRE           GHNAILRTLahlklplkmvltimnll-nesetsvyin-n----s---lsk-sftskrGTK
Zepp          GRAWIERCMaavgfgpgvqrwvhilh-sgttsrvafn-g----w---htd-afpvaaGVF
BDDF           HNKLWKILkemgipdhltcllrnly-agqeatvrtg-h----g---ttd-lfqigkGVC
JAM1           RIELWKIMdensfpgkltrliratm-dgvqncvki--s----g---hss-sfesrrGLR
RTE-1          HQAIWKSLdeqgadgayidllkecy-kncttnftp--f----h---rpv-avpvtkGVR
RTE-2          HSAVVSSLekagadpamrkmiqemm-dggqaeitv--h----d---kkl-kvnlctGVR
CR1-chicken    HHILISKLegcgfdgwtthwirnwl-kgrrqrvvtn-g----s---msr-wrpvmsGVP
Q              HAILLAKLdklgipsplvqwlksyl-ihrtyivkid-k----h---msk-eivsssGVP
T1             HSLLLAKLsklgfgdgiiswlssyl-snrscrvktg-s----y---lse-effctsGVP
CR1-Turtle     HSILISKLkkygldewtikwienwl-xhraqrvvin-g----s---mss-wqpvtsGVP
SR1            HICLIKKLrrlgikpplidwlssyl-enrhfkvrvn-f----t---lsq-amecpsGVP
Sam6           HNLLILKLknfgiddnlcdwfqsfi-snrtstvkvs-n----s---mstckysissGVL
Sam3           HNLLLCKLelfgldvkmcnwfrsfl-snrtssikvc-d----h---vsknklevlsGVP
BmCR1-Bombyx   HKALLSKLpsyglperlcrwigsfl-aerrvkvvvd-g----s---csd-prtvnaGVP
Jocky-Drosmel  HPGLLYKakrl-fppqlylvvksfl-eertfhvsvd-g----y---kss-ikpiaAGVP
Jocky-Dfunebr  HPGLLYKaksl-lspqlfqliksfw-egrkfsvtad-g----c---rss-vkfieAGVP
TART           HEGLLLKLaki-lpynlyiilesyl-tnrtfevkdqag----e---tsr-tgqigaGVP
G              HPGLHYKIkth-lpgshfaflksft-egrefqvccg-t----a---tst-prpiraGVP
Doc            LDGLLFKIikl-lpqnthkllksyl-ynrvfaircd-t----s---tsr-dcaieaGVP
F              LDGLMFKIkis-lpesthkllksyl-ydrkfavrcn-t----a---tst-vhtieaGVP
YAKPs1         HEGLLYKIkns-fpsnmflilksyl-rdryffvqsg-e----a---lsk-lcpiaaGVP
BS             HDGLISKVkkl-fpapyygvlksyl-edrrfmvrvr-n----s---ysi-prvmraGVP
Helena-Dyak    HVGLLCKIktm-lpapyfcilksyl-egrefkitvr-n----n---yst-vypmraGVP
Helena-Dmau    HVGLLCKIktl-lpepyfcilksyl-eerqfkitvr-n----s---yss-iypiraGVP
AMY            HNGLIFKLfnmgvpdslvliirdfl-snrsfryrve-g----t---rss-prpltaGVP
```

```
JuanA          HEGLIVKLknfnfptyivriiqsyl-snrtlqvnyq-n----s---rse-rlpvraGVP
JuanC          HKGLIAKLkrfnfpiyivkiiqnyl-tdrtlqvcyq-n----s---ksd-qlpvraGVP
NCR1Cth        HNGLLYKMlklrfplpliktvrsfl-sertfsvfik-g----q---fse-ikeikyGVP
BmRTE-d24      HHQLFSLLcdigldgkdvriirnly-ekqvatirve-n----e---etd-qveicrAVR
BmRTE-d25      WSSMFDVLidmgvpshlillirnly-ldgscfvkld-n----r---rsr-sfhtehGVR

BmRTE-d01     RGDIISPKLFTNALEDV--FK--TL---D--WNGr-----------GI------------
BmRTE-d02     RGDVISPKLFITALEDS--FK--LL---E--WQGl-----------GI------------
BmRTE-d03     RGDVISPKLFTAALEDS--FK--LL---E--WQGf-----------GI------------
BmRTE-d04     RGDVISPKLFTAALEDV--FK--TL---D--WkTC-----------GI------------
BmRTE-d05     EGCILSPILFNVYGEYV--MR--KA-leE--WEG------------GI------------
BmRTE-d06     HGCILSPLLFNIYTEYI--MR--IV-ldD--WDK------------GI------------
BmRTE-d07     KGCILSPVLFNIYGEYI--MR--RT-ceG--WDG------------GV------------
BmRTE-d08     KGDVISPKLFTAALEDV--FK--VL---D--WkGL-----------GI------------
BmRTE-d09     -GDVISPKLFTNALEDM--FK--TL---D--WKKm-----------GI------------
BmRTE-d10     KGDVISPKLFTAALEDV--FK--TL---D--WSKl-----------GI------------
BmRTE-d11     HGCIISPLLFNIYTELI--MR--IA-leN--WSD------------GM------------
BmRTE-d12     -GCILSPILFNIYGEYI--MR--KT-leN--WDG------------GI------------
BmRTE-d13     SGSVLSPFLFGMVIDSL--TE---------------------------------------
BmRTE-d14     TGDALSCLLFNVALDKC--IR--DS---------------AIE---TT-------G----
BmRTE-d15     SGCVLAPTLFALXFAVV--VR--EV-LQT--ISQ------------GVRTRfrtdgslfn
BmRTE-d16     HGCIVSPILFNIYGEYI--MR--KT-leE--WDG------------GV------------
BmRTE-d17     -GCLLSPLLFIMLLDDI--MR--EV-VTT---PR------------GI------------
BmRTE-d18     EGCILSPQLFNLIGEHI--MR--LV-leN--WEG------------GI------------
BmRTE-d19     HGCIVSPLLFNAYTEII--MR--IT-leN--WTD------------GV------------
BmRTE-d20     TGDPLSPKLFSATLEHV--FR--QL---E--WDDy-----------GI------------
BmRTE-d21     EGDPLSPKIFIAVLEMV--FS--KL---N--WERK-----------GL------------
BmRTE-d22     EGDPLSPKLFIAVLESI--IS--QL---D--WKNy-----------GL------------
BmRTE-d23     EGDPLSPTLFNAVLEHI--FR--QL---N--WDHl-----------GL------------
HmRTE-e01     SGSALSPFLFNVVLDTV--SA---------------------------------------
Slacs         AGMVLGPLLFSIGTLAT--LR--RL-----------------------------------
CZAR          AGMVLGPVLFSIGTIAT--LR--QL-----------------------------------
CRE-1         SGMVLGPLLYATGMAAA--IG--PV-----------------------------------
CRE-2         AGMVLSPLLFANAMSGI--IR--PL-----------------------------------
R2-Drosmel    -GDPLSPILFNLVMDRL--LR--TL------PSEI-----------GA------------
R2-Drossim    -GDPLSPILFNLVMDRL--LR--NL------PSEI-----------GA------------
R2-Drosyak    EGDPLSPILFNLVMDRM--IR--KL------PKEV-----------GV------------
R2-Drosmer    -GDPLSPVLFNLIIERL--LR--SL------PKDI-----------GV------------
R2-Earwig     EGDPLSPILFNLVMDRM--IR--KL------PKEV-----------GV------------
R2-Bmori      -GDPLSPILFNVVMDLI--LA--SL------PERV-----------GY------------
R2-Isopod     KGDPISPFLFNLMIDYI--LS--GT------QAGV-----------GV------------
Dong          TGDSLSPLWFCLALNPL--SH--QL------HNDR-G---------GI------------
R4            RGDSLSPTLFVLCIAPI--SY--AL------NKGV-GQC-qss-s-gw-------s----
L1-Rat        EGCPLSPYLFNIVLEVL--AR--pi------rKQK-----EIK---GI------------
L1-Huamn      EGCPLSPLLFNIVLEVL--AR--AI------RQEK-----EIK---gi------------
L1-Mouse      EGCPLSPYLFNIVLEVL--AR--AI------RQQK-----EIK---gi------------
L1-Dog        EGCPLSPLLFNIVVEVL--AS--AI------RQQK-----Dfr---gI------------
SwimmerMedaka KGCNMSALLFALYIEPL--GQ--WI------RQRA-----DIk---gv------------
SwimmerPupfis EGCGASPLLFALFLEPL--DQ--WI------RQRS-----DIs---gv------------
Tx1           RGCPLSGQLYSLAIEPF--LC--LL------RKR-------LT---gl------------
Cin4          EGDPLSPFLFILAMDPL--QR--MI---E--RAAh---------e-gl------------
Ta11          -GDPLSPFLFVLCSEGL--TH--LM---N--RAER---------Q-Gl------------
DRE           GGDPISPTIFALVVECM--AT--TI------INDR-----------CI-------N----
Zepp          GGSPLSPLLFVLARAPM--AA--HA---------------Rml-a-gq----------la
BDDF           GCILSPCLFNFYAEYI--MR--NAgleE--AQA------------GI------------
JAM1           GDGLSCM------SCC--HA--ES---------------LYL---TV-------E----
RTE-1          GDPISPNLFSACLEHV--FR--KL---S--WIElkgeaedydtipGM------------
RTE-2          GDSASPALFSAALQAI--LT--DC---DNEfagV-----------GI------------
CR1-chicken    GSXVXPVLFNIFINDI--DD---------------------------------------
Q              GSNIGPLLFILFINDV--TL---------------------------------------
T1             GCVLSPLLFSLFINDV--CN---------------------------------------
CR1-Turtle     GSVLGPVLFNIFINDL--ED---------------------------------------
SR1            GSILGPLLFLIYINDL--PQ---------------------------------------
```

```
Sam6           GTVTGPFLFLIYINDL--LE---------------------------------------
Sam3           GSVCGPFLFLIYINDL--LG---------------------------------------
BmCR1-Bombyx   GCVLSPTLFILHINDLLHTS---------------------------------------
Jocky-Drosmel  GSVLGPTLYSVFASDM--PT--HT---------------PVT-----------------
Jocky-Dfunebr  GSVLGPTLYSIFTADM--PN--QN---------------AVT-----------------
TART           GSNLGPLLYSIFSSDM--PL--PY---------------IYR-----------------
G              GSVLGPILYTLYTADL--PITPSR-----------------------------------
Doc            GSVLGPILYTLYTADF--PI--DY-----------------------------------
F              GSVLGPTLYLIYTADI--PT--NS-----------------------------------
YAKPs1         GSVLGPILYLIHTADL--PI--SN-----------------------------------
BS             GSVLGPLLYSVFTADL--PC--PN---------------AYH-----------------
Helena-Dyak    GSVLGPLLYSLYTADI--PC--PN---------------FDH-----------------
Helena-Dmau    GSVLGPLLYSLYTADI--PC--PS---------------FEH-----------------
AMY            GSVLSPLLFSLFVNDI--PR---------------------------------------
JuanA          GSILGPILYNIFTSDL--PE---------------------------------------
JuanC          GSILGPILYNIFTSDL--PD---------------------------------------
NCR1Cth        GAVLSPTLYNIFTYDI--VR---------------------------------------
BmRTE-d24      GCVLSPLLFNIYSEAV--MS--KA-LEn--lEV------------GI------------
BmRTE-d25      GCILSPKLFNIYGEYI--MR--RA-LEG--Wng------------GI------------

BmRTE-d01     R-------NINGEYIS-HLR--FADDIVI--M--------------AES-LQD--LQEMV
BmRTE-d02     R-------NINGEYIT-HLR--FADDIVV--M--------------AES-LED--LGRML
BmRTE-d03     R-------NINGEYIT-HLR--FADDIVV--M--------------AES-LED--LGQML
BmRTE-d04     R-------NVNGEYMS-HLR--FADDIVL--M--------------SES-LED--LSRML
BmRTE-d05     E-------SVGGIKIS-NLR--YADDTTL--F--------------ASS-EKE--LADLF
BmRTE-d06     H-------SVGGRKIS-NLR--YADDTTL--L--------------AST-RDE--IEVLL
BmRTE-d07     K-------TIGGVKLS-NLR--YADDTTL--L--------------AAN-ESE--MAALM
BmRTE-d08     K-------NINGEYIT-HLR--FADDIVI--M--------------AET-MED--LSTML
BmRTE-d09     --------NINGQYIS-HLR--FADDIVL--M--------------AES-LQD--LQQML
BmRTE-d10     K-------NVNGEYLS-HLR--FADDIVM--M--------------AES-LED--LSCML
BmRTE-d11     H-------TIGGRKIS-NLR--YADDITL--V--------------ASG-VSQ--MEELL
BmRTE-d12     --------TIGGVKVT-NLR--YADDTTL--L--------------ATT-EAE--MTELL
BmRTE-d13     S-------VAQSSASW-TFI--YADDVAI--C--------------TES-RTK--LREAL
BmRTE-d14     T-------NIYYKSAQ-VLG--YADDIDV--I--------------GRS-ALA--VESAY
BmRTE-d15     SarlkartkvsyaLIT-EIM--YADDLCF--L--------------AES-PAG--LQQLM
BmRTE-d16     H-------TVGGVKIS-NLR--YADDTTL--V--------------ASS-EEE--MEELL
BmRTE-d17     --------EWSENIlE-DLD--YADDIVL--M--------------TPT-LDQ--MQAKL
BmRTE-d18     E-------RVGAHRIS-NLR--FADDTTI--I--------------GTS-ERE--LHELL
BmRTE-d19     H-------AIGGYRIA-NLR--YADDTTL--F--------------ATD-AQC--LGELL
BmRTE-d20     T-------NINGVLLN-HLR--FADDLIL--I--------------SEN-PET--LQKMI
BmRTE-d21     E-------NINGNfIN-HLR--FADDIIL--L--------------SES-AKE--MESMI
BmRTE-d22     E-------YIKGDCLS-HLR--FADDLVL--L--------------SET-GGN--LERMI
BmRTE-d23     E-------NINGARLN-HLR--FADDLVL--L--------------EEN-PAA--IELMM
HmRTE-e01     S-------HIQDQPPW-LMM--YADDIAL--I--------------AEN-RLT--LERKV
Slacs         A-------QQTFPEAQ-FTA--YLDDVTV--A--------------APP--EE--LKNVC
CZAR          A-------ESSFSNAS-FTA--YLDDVTV--A--------------APP--GM--LGKVC
CRE-1         S-------RQRIPGVP-VTA--YIDDITL--A--------------ASG--AE--GARAA
CRE-2         A-------MEMHPRVK-VVA--YLDDVTL--I--------------GPH--AA--VQDFL
R2-Drosmel    --------KVGNAITN-AAA--FADDLVL--F--------------AET-RMG--LQVLL
R2-Drossim    --------KVGNAITN-AAA--FADDLVL--F--------------AET-RMG--LQVLL
R2-Drosyak    E-------NVGSKHYN-GLT--FADDLLL--F--------------ATT-PEG--LQSSI
R2-Drosmer    --------HVGNAKVN-ACA--FADDLML--F--------------AST-PKG--LQELL
R2-Earwig     E-------NVGSKHYN-GLT--FADDLLL--F--------------ATT-PEG--LQSSI
R2-Bmori      --------RLEMELVS-ALA--YADDLVL--L--------------AGS-KVG--MQESI
R2-Isopod     K-------GVGERLVS-SLA--YADDLAL--L--------------ASS-RRG--LNANL
Dong          T-PIKQQ-dntetiIS-HLI--YMDDIKL--Y--------------AKN-DKE--MKKLI
R4            R-------aGYGFEIG-HQF--YMDDLKL--Y--------------ART-PAM--LDSQI
L1-Rat        E-------QIGKEEVK-ISL--FADDMIV-YL--------------SDP-KSS--TREQL
L1-Huamn      E-------qLGKEEVK-LSL--FADDMIV-YL--------------ENP-IVS--AQNLL
L1-Mouse      E-------qIGKEEVK-ISL--LADDMIV-YI--------------SDP-KNS--TRELL
L1-Dog        E-------QFGKEEVK-LSL--FADEMIL-YI--------------ENP-KVS--TPRLL
SwimmerMedaka K-------KVSGKEQK-LSL--FADDLLL-TI--------------SQP-TKT--LPIIM
SwimmerPupfis E-------TMTAGEQK-LAL--FADDVLI-FL--------------TQP-NQT--LPRLM
```

```
Tx1           R-------vLKEPDMR-VVLSAYADDVIL--V--------------AQD-LVD--LERAQ
Cin4          EgQV----LPNGAKFR-CSL--YADDAGV--F--------------VRA-DKL--DLKVL
Ta11          -sgirf--sENGPAIH-HLL--FADDSLF--M--------------CKA-VKE--EVTVI
DRE           G-------gVTKETIK-ILQ--FADDTAT--I--------------AYN-FMD--HFLMN
Zepp          GQPIRL--PSGEPAPV-MHQ--HADDTSV--H--------------ART-PGM--LRSCW
BDDF           -------KIARRNIN-NLR--YADDTTL--M--------------AES-EEE--LKSLL
JAM1           -------ARFSRDPY-NLF--FADDMML--L--------------GEN-LKQ--WSDLF
RTE-1          -------RVNGRNLT-NLR--FADDIVL--I--------------ANH-PNT--ASKML
RTE-2          -------NVEGRHIR-RLE--FADDVVL--I--------------CST-PGE--VQERL
CR1-chicken    ----------GIECT-LSK--FADDTKL-----------SGAVDTEeg-rdA--IQRDL
Q              ----------ALPPDSISL--FADDAKI-----------FAPINNTGd-ctf--LQDCI
T1             ----------VLPPDGHLL--YADDIKI-----------FlpvsSSSD-CMS--LQHYL
CR1-Turtle     ----------GVDCT-LSK--FADDTKL-----------GGVVDTleg-rDR--IQKDL
SR1            ----------QVSSD-LLL--FADDVKL-----------WREIRNHnd-ilV--LQEDL
Sam6           ----------QFPAD-VHVTAFADDVKI----------------SSEN-IES--IKKSI
Sam3           ----------MLPPD-VQISAFADDIKI----------------YGDN-SNS--IQKSI
BmCR1-Bombyx   ----------GIHC-------YADDSTVdalyfgrsnISRDHVDecrn-klvsEIESSL
Jocky-Drosmel  -------EVDEEDVL-IAT--YADDTAV--L--------TKSKSilaa-tSG--LQEYL
Jocky-Dfunebr  -------GLAEGEVL-IAT--YADDIAV--L--------TKStciveA-TDA--LQEYL
TART           -------PSPTERIM-LST--YADDTIV--L--------SsdtlaTAA-TRN--NENYL
G              ------------SLT-VAT--YADDTAF--L--------ASASdpqea-STI--ILSQL
Doc            ------------NLT-TST--FADDTAI--L--------SRSKcPIKA-TAL--LSRhl
F              ------------RLT-VST--FADDTAI--L--------SRSRSPiqa-taQ--LALYL
YAKPs1         ------------LAT-TGT--FADDTAV--L--------VSHtdhkvA-SAM--LQTCL
BS             -------MADPRKAL-LAt--YADDIAL--L--------yssncCNEA-ARG--LQEYL
Helena-Dyak    -------MEAPYKAL-MAT--YADDIAV--V--------yssgdSREA-AKG--LQEYI
Helena-Dmau    -------MAAPNRTL-IAT--YADDIAV--V--------ynsrdIREA-ANG--LQEYI
AMY            ----------SPPTH-LAL--FADDTTV--Y--------YSSRNksli-aKK--LQSAA
JuanA          ---------LPQGCQ-KSL--FADDTGL--S--------AKGRSlrvi-cSR--LQKSL
JuanC          ---------LPPGCQ-KSL--FADDTSI--S--------AKARSlrvi-tRR--LQKSL
NCR1Cth        ---------ETTN-N-IAL--FADDTAL--Y--------hsAENSADI-VTQ--LLhtg
BmRTE-d24      -------GINGRVVN-NLR--YADDTIL--I--------------AAS-EAD--LQAIV
BmRTE-d25      -------SVGGETLT-NLK--YADDTTM--L--------------ASD-EEE--MAILL

BmRTE-d01     RSLNAASQRV-----GLGMNL-DKT---------K--VMF---NG-----------NVI-
BmRTE-d02     RDLSRVSQQV-----GLKMNM-DKT---------K--IMY---NV-----------YVA-
BmRTE-d03     RDLNRVSQRV-----GLKMNM-DKT---------K--IMF---NV-----------HIK-
BmRTE-d04     RDLNAASRCV-----GLRMNL-DKT---------K--VMF---ND-----------KIV-
BmRTE-d05     ERVEYESSLV-----GLSVNK-SKT---------K--VMI---VD-----------RTS-
BmRTE-d06     HRLETTALDF-----GLAINR-DKT---------K--MMI---VD-----------RAN-
BmRTE-d07     KKLEKISLEL-----GLAINR-SKT---------K--VMV---ID-----------RMN-
BmRTE-d08     KDLSRASIRV-----GLNMNK-EKT---------K--IML---NA-----------HVA-
BmRTE-d09     -GLADSSRRI-----GLRMNM-DKT---------K--VMF---NC-----------YIS-
BmRTE-d10     KELNAASRRV-----GLGMNL-DKT---------K--VMF---ND-----------HII-
BmRTE-d11     HRVERVSLDF-----GLKINR-NKT---------S--VMI---VD-----------RAN-
BmRTE-d12     -RMEHIGLEM-----GLALNR-SKT---------K--IMV---VD-----------RTK-
BmRTE-d13     SLWKQQLQAG-----GLILSV-AKT---------H--YMS---FN-----------dPD-
BmRTE-d14     TALEASSLEA-----GLQVNA-DKT---------K--FLR---VS-----------RDl-
BmRTE-d15     SVFHLACRKF-----GLKISV-NKT---------E--VMS---LD-----------SHG-
BmRTE-d16     HRLVIVSEEI-----GLKINQ-SKT---------K--IMI---VD-----------KYG-
BmRTE-d17     -DLRMSAEKR-----GLRINT-NKT-------------VD---MR-----------VMS-
BmRTE-d18     ErvevvsKDLKDKNYGLCLNR-SKT---------K--LMF---VD-----------RPR-
BmRTE-d19     HRMERVSLEF-----GLRINR-NKT---------K--VMI---VD-----------RAM-
BmRTE-d20     TQLVRESEKV-----GLSLNT-SKT---------K--LMT---NY-----------KKV-
BmRTE-d21     ESLKTMSCEV-----GLEMNL-DKT---------K--IMS---NS-----------IKH-
BmRTE-d22     ESLHEASRQV-----GLEINL-KKT---------N--IMT---NS-----------CRR-
BmRTE-d23     ESLANISKEA-----GLEING-SKT---------K--LMT---NS-----------REI-
HmRTE-e01     SLWKGTLENG-----GLKLNV-SKT---------E--YMA---CG-----------SR--
Slacs         AATAEAMEAL-----GIVNNA-DKT---------E--VLE---LT-----------GDT-
CZAR          AATSRAMRAL-----GIETNE-DKT---------E--VL---------------------
CRE-1         SAYADALETV-----GVVTNA-RKS---------M--VVG---PE-----------GTR-
CRE-2         AEAGPQLSRV-----GFDINP-AKSHHLAKLEVPE--ALS---VS-----------GRT-
R2-Drosmel    -KTLDFLSIV-----GLKLNA-DKC-----F------TVG---IK-----------GQP-
```

```
R2-Drossim    -RTLDFLSLV-----GLKLNA-DKC-----F------TVG---IK-----------GQP-
R2-Drosyak    EIVHLFLLEC-----GLLINK-QKS-----F------VLT---VK-----------AYP-
R2-Drosmer    -TTVKFLSSV-----GLTLNA-DKC-----F------TIS---IK-----------GQP-
R2-Earwig     EIVHLFLLEC-----GLLINK-QKS-----F------VLT---VK-----------AYP-
R2-Bmori      -AVDCVGRQM-----GLRLNC-RKS-----A------VLS---MI-----------PDG-
R2-Isopod     KSVLARARSV-----NLALGI-NKC--AT-IGK-R--WLG---RE-----------KKM-
Dong          TTTTIFSNDI-----SMQFGL-DKC--KT-V---H--IIK---GK-----------VQP-
R4            RVVSEVSEAM-----GLHLNL-SKC-----A---A--PHg---ag-----------gaq-
L1-Rat        ELINNFSKVA-----GYKINS-NKS---------VAFLYT---KE-----------KQA-
L1-Huamn      EPISNFSKVS-----GYKINV-QKS---------QAFLYT---NN-----------RQT-
L1-Mouse      ELINSFGEVV-----GYKINS-NKS---------MAFLYT---KN-----------KQA-
L1-Dog        ELMQHCGSVA-----GYIINA-QKS---------VAFLYT---NN-----------ETE-
SwimmerMedaka KSLKDFGTLS-----GYKINV-NKT---------Q--VLT---LN-----------YSP-
SwimmerPupfis EVLEEYGSLS-----GYKINV-NKT---------Q--ILR---LN-----------FNP-
Tx1           RCQEVYAAAS-----SARINW-SKS---------SG-LLE---GS-----------LKV-
Cin4          ERILEAFEWC---S-GLKINF-EKT---------E--IFP---IR-----------YPE-
Tal1          -SIFKVYGDV---T-GQRINY-DKS---------S--ITLGALVD-----------EDC-
DRE           GWIKKFCQAT-----SAKINQ-TKC---------S--CIT---FK-----------WNT-
Zepp          GPSVGLHCAA---T-GARLQR-SKS---------Q--ALG---LA-----------ASA-
BDDF           KVKVESEKV-----GLKPNI-QKM---------K--IMA---SG-----------PIT-
JAM1           RLKREATRV-----GLMVNV-SKT---------K--YML---VG-----------GTE-
RTE-1          ELVQKCSEV-----GLEINT-GKT---------K--VLR---NR-----------FAD-
RTE-2          ILDRISSNY-----GLKINQ-SKT---------V--LLK---NK-----------FCR-
CR1-chicken    RLERWARVN-----LMRFNT-AKC---------R--VLH---LG-----------WRN-
Q              IFCSWCKRN-----GLTICI-EKC---------Y--CVS---Fs-----------RCR-
T1             AFVHWCSSN-----LLRLCP-DKC---------S--VIS---FS-----------HSl-
CR1-Turtle     KLEDWAKRN-----LMRFNK-DKC---------R--VLH---LG-----------WKN-
SR1            RLQSWADDN-----GLTFNT-SKC---------K--VVH---LR------------HV-
Sam6           IIEQWCDIW-----KLKLAE-NKT---------Q--VLH---IG-----------KLN-
Sam3           IVTDWCRKW-----SLNLAE-NKS---------V--VVH---YG-----------KNN-
BmCR1-Bombyx   KISDWGGRN-----LVQFNP-SKT---------Q--VCA---FT-----------AKK-
Jocky-Drosmel  AFQQWAENW-----NVRINA-EKC---------A--NVT---FA--NR-------TGS-
Jocky-Dfunebr  AFQEWAVKW-----NVSINA-GKC---------A--NVT---FT--NA-------IRD-
TART           SFSDWADKW-----GISVNA-AKT---------G--HVI---FTLKND-------LPT-
G              ALDPWLKRW-----TIAVNA-DKS---------S--QTT---FS--LR-------RGD-
Doc            sVERWLADW-----RISINV-QKC---------K--QVT---FT--LN-------KQT-
F              DIKKWLSDW-----RIKVNE-QKC---------K--HVT---FT--LN-------RQD-
YAKPs1         DIALWLKKW-----RIKANE-TKS---------V--NVT---FT--KR-------KDT-
BS             TLAAWCKRW-----NLKVNP-QKT---------I--npC---FTLKTL-------SPV-
Helena-Dyak    ALAAWCKRW-----NLKINA-TKT---------T--NLC---FTLKTl-------iKK-
Helena-Dmau    ALAAWCKRW-----NLKINQ-LKT---------T--NPC---FTLKTl-------iPN-
AMY            ALGQWFRKW-----RIDINP-AKS---------T--AVL---Fq-----------rgss
JuanA          IFSSYLQKW-----EISPNA-SKT---------Q--LII---FP--HKPKALYLkpss-
JuanC          IFNSYLKEW-----KITPNVlSKT---------Q--LII---FP--HkpradflKPKS-
NCR1Cth        KVQQYMDKW-----KINLNK-QKS---------Q--AIF---IT--NR----YSKQLP-
BmRTE-d24      KVNECSEEA-----GLSINI-SKT---------K--FMV---VS-----------RNP-
BmRTE-d25      RVEEESAKL-----GLII------------------IMI---ID-----------RAQ-

BmRTE-d01     R-----------P------RPID----VGG----T-------P--LEVVQEYIYLGQTL
BmRTE-d02     R-----------P------TIVT----VGS----S-------T--LEVVDEYIYLG---
BmRTE-d03     R-----------P------TAVT----VGN----S-------T--LEVVDDYIYLGQVV
BmRTE-d04     R-----------P------GQVT----ISN----A-------V--IEEV-DFVDLGQAI
BmRTE-d05     E-----------Q------LSRT----GEL----S-------D--LEFVSEFIYLGSLL
BmRTE-d06     H----------IN------QPEV----QHI----A-------G--CEVVNSYVYLGSTI
BmRTE-d07     K-----------K------LEHT----GSL------------H--LETTERFIYLGSMI
BmRTE-d08     K-----------P------TPVK----IGG----S-------T--LEVVDEYIYLGHTV
BmRTE-d09     ------------P------GPIV----VKD----C-------P--LEAVDEYLYLGQTL
BmRTE-d10     K-----------P------GPVI----VES----A-------V--LEVVSEYTYLGQII
BmRTE-d11     H----------NN------SPEV----TKI----A-------N--CDVVQSYIYLGALI
BmRTE-d12     ------------K------LELS----GTL------------N--LELVDNFIYLGSNI
BmRTE-d13     S-----------P-GDN--SPIS----IDG----Q-------L--VNMCDQYKYLGTMM
BmRTE-d14     T-----------REDT---AHKN----IGQ----H-------T--FGSVNEFVYLG---
BmRTE-d15     S-----------H------EALT----IKL----G-------EDVLKQVDKFRYLG---
```

```
BmRTE-d16     H-----------T------LNEN----NIL----G-------Q--YDIVKTFVYLGSII
BmRTE-d17     ------------K------NTTP----LKL----Q-------DCVLKSAQKFTYLGSSI
BmRTE-d18     E-----------S------R---------------------------------------
BmRTE-d19     H----------NN------SPDV----TQI----A-------G--CDVVQSYIYLGALI
BmRTE-d20     T-----------P--------IK---PYNT----A-------K--LEYVNEYTYLGQII
BmRTE-d21     E-----------P--------IY----LDE----K-------P--LEYVDSYIYLGKQI
BmRTE-d22     E-----------T--------IS----LEH----K-------P--ILYVEQYIYLGKQI
BmRTE-d23     E-----------D--------VM----VDG----I-------K--IEYVKEYNYLGQII
HmRTE-e01     S--------------DS--STIL----IGP----E-------P--AVKSEKFRYLGSVM
Slacs         A----------------------------------------------------------
CZAR          A----------------------------------------------------------
CRE-1         S-------------VG-----------IGG-VD------------LPVVAEARILGAHF
CRE-2         A-------------IP-----------IAQ-------------------GVVRILG---
R2-Drosmel    ------------K--QK--CTVl----eaq-sf-yvgSSEIPS--LKRTDEWKYLGINF
R2-Drossim    ------------K--QK--CTVl----eaq-sf-yvgSREIPS--LKRTDEWKYLGINF
R2-Drosyak    E-----------K-LKK--Tavi----vte-kY-MLDRHILPA--IDREKlfhYLGVPF
R2-Drosmer    ------------K--QK--VTVV----EQR-TF-cigrarv-q--LKRSEEWKYLGIHF
R2-Earwig     E-----------K-LKK--Tavi----vte-kY-MLDRHILPA--IDREKlfhYLGVPF
R2-Bmori      ------------H-RKK--Hhyl----ter-tF-NIGGKPLRQ--vscVERWRYLGVDF
R2-Isopod     K-----------I-LDR--EPFL----LEg------vaipv----yrwNNIYKYLGI--
Dong          T--------------GD--YTID----DTT-QY---------G--DGTkclYKYLGF--
R4            R-----------e------avEG----AEG-SR-K-GEIPI----LGLRSTYKYLGV--
L1-Rat        E-----------E------KEIR----ETT-PF------------IIDPNNIKYLGVTL
L1-Huamn      E-----------E------SQIM----NEL-PF------------TIASKRIKYLGIQL
L1-Mouse      E-----------E------KEIR----ETT-PF------------SIVTNNIKYLGVTL
L1-Dog        E-----------E------REIK----ESI-PF------------TIAPKSIRYLGINL
SwimmerMedaka K-----------P------QNIK----DEY-KW------------EWQADSIKYLGI--
SwimmerPupfis E-----------S------TRIK----NMY-KW------------IWDSEHIKYLGVVL
Tx1           R-----------D------FLPP----AFR-DI------------SWESKIIKYLGVYL
Cin4          E-----------S-LWSN-LMEV----FPG---------------KYSNFPGKYLGLPL
Ta11          ------------K-vW---IQAE----LGI---------------TNEGGASTYLGLP-
DRE           G------------------RTLY----------------------TVIKSNERYLGFDF
Zepp          G-------------ISP--GPIQ----SRG--------vTF----AASSDGVKHLGIPL
BDDF           -----------S------REID----GE-------------T--VETVSDFMFLGSKI
JAM1           -----------CDRARlgssVT----IDG----D-------T--FEVVDEFVYLGSLL
RTE-1          -----------P------SEVY----FGspSPTT-------Q--LDDVDEYIYLG---
RTE-2          -----------S------QDVL----FNG----S-------P--IIPVPGCRYLGRW-
CR1-chicken    ------------------PRHLYR--LEG-AV------------LESSSAEKDLGVLM
Q              ------------------SPVTGTYFMDG-TA------------VNRQNHAKDLGVLL
T1             ------------------SPISFNYTLSN-SS------------LSRVLSIRDLGIIL
CR1-Turtle     ------------------PMHSYR--LGT-DE------------LGSSSAEKDLGVTV
SR1            ------------------ADHSYN--LGN-SP------------LEVSQVEKDLGVLV
Sam6           ------------------PKTDYL--VNG-HK------------ISVCSKARDLGIWV
Sam3           ------------------PKFVYT--ANG-II------------IAKKKSVKDLGIFV
BmCR1-Bombyx   ------------------APFVaSPLFGN-TH------------LCAKSNMGILGVDI
Jocky-Drosmel  -----------C------PGVS----LNG-RL------------IRHHQAYKYLGITL
Jocky-Dfunebr  -----------C------PGVT----ING-SL------------LSHTHEYKYLGVIL
TART           -----------S-----LRtmk----IKG-QV------------IKIESKQSYLGVIL
G              -----------C------PPVT----LNG-ET------------IPTSSSPKYLGL-L
Doc            -----------C------PPLV----LNN-IC------------IPQADEVTYLGVHL
F              -----------C------PPLL----LNS-IP------------LPKADEVTYLGVHL
YAKPs1         -----------C------PPVQ----LNR-IQ------------VPQAEHVKYLGLYL
BS             -----------T------APIE----LEG-VI------------LDQPSQAKYLGITL
Helena-Dyak    -----------T------PPLQ----LEG-VT------------LDQPLQATYLGITL
Helena-Dmau    -----------T------PAIR----LEG-VT------------LNQPLQATYLGITL
AMY            rissrIRRRNLT------PPIT----LFR-QP------------IPWARKVKYLGVTL
JuanA          -----------r------hvVT----MRG-VP------------INWSDEVKYLGLML
JuanC          -----------H------HIIK----MNE-VN------------LKWEDQVKYLGL--
NCR1Cth        -----------R------NNIK----FLN-EN--------------------------
BmRTE-d24      -----------D------LSSS----VSV----A-------GKQLERVRQYKYLGAWV
BmRTE-d25      -----------L------FPRS----DAL----S-------G--YEKVAEFIYLGSQI
```
