# Supplementary material for: Generation of microsatellite repeat families by RTE retrotransposons in lepidopteran genomes
Source: BMC Evol Biol. 2010 May 17;10:144. doi: 10.1186/1471-2148-10-144 (PMC2887409; doi:10.1186/1471-2148-10-144)
Supplement: Additional file 8 — Amino acid sequence alignment of the Reverse Transcriptase (RT) conserved domain from plant and animal non-LTR RTE clade elements used in RTE clade phylogenetic analysis. The Neurospora Group II intron (GenBank:S07649) RT conserved domain was used as an outgroup. Amino acid sequence alignment was performed using the Kalign program [51,52] in EMBL-EBI using default parameters. The snake (Vipera ammodytes) Bov-B LINE, fluke (Schistosoma) SR2, plants (Aegilops, Hordeum), and Bombyx mori BmRTE-d24 and -d25 were from [22] with slight modifications. BCCD from cow, JAM1 from Aedes, RTE-1 and RTE-2 from Caenorhabditis elegans were previously used in the phylogenetic analysis by Malik et al. [23]. All remaining RTE/Rex3 elements were as provided in Additional File 2. [file 1471-2148-10-144-S8.PDF]

Additional File 8 (.pdf): Amino acid sequence alignment of the Reverse Transcriptase (RT) conserved domain from plant and animal non-LTR RTE clade elements used in RTE clade phylogenetic analysis.

The *Neurospora* Group II intron (GenBank:S07649) RT conserved domain was used as an outgroup. Amino acid sequence alignment was performed using the Kalign program [51, 52] in EMBL-EBI using default parameters. The snake (*Vipera ammodytes*) Bov-B LINE, fluke (*Schistosoma*) SR2, plants (*Aegilops*, *Hordeum*), and *Bombyx mori* BmRTE-d24 and -d25 were from [22] with slight modifications. BCCD from cow, JAM1 from *Aedes*, RTE-1 and RTE-2 from *Caenorhabditis elegans* were previously used in the phylogenetic analysis by Malik et al. [23]. All remaining RTE/*Rex3* elements were as provided in Additional File 2.

```
Pristionchus-a01  RSEIVTLY-KGnGDIRECGNYRGIKLISHTMKIYEKCIDRRLREIMT--LNPSQYGFVSG
Cbrenneri-a01     DSITTLIP-K-KGDLRDISNYRPISVLPSTFKLLTRVILGRIQTTLEEQQPPEQAGFRRG
Anolis-a01        TSVTVPIW-KGiGDVANCSLYRPIRLLCHTMKIFKHILDNRLREIIM--MTENPCGFIKE
Aplysia-a01       -AIVVPIwKK-KGSKKDCNTYRGIPLLSHVGKMWAKILERRTRAKTEHLLSDAQFGFRKG
Branchiostoma-a02 KSIILSLY-KgKGEALDRGNYRGLKLTDHVMKLLERVLDSAIRKMVN--IDDLQFAFVPG
Danio-a01         TGVVVPIF-K-KGDRRVCSNYRGITLLNLPGKVYARVLERRIRPMVEPRIQEEQCGFRPG
Gasterosteus-a01  -GVVVPLF-K-KGDQRVCANYRGITLLSLPGKVYSKVLERRVRPIVEPRIEEEQCGFRPG
Mchenga-a01       TSITVPIW-KgKGDVAECSNYRPIRLLSHTMKVFERIIDARMREIVN--LTPNQCGFIRG
Nematostella-a01  -SYIINLY-KgKGDALDRGSYRGLKLTEHCLKVIERVLEKIIRSLVE--IDEMQFGFVHG
Oryzias-a06-Rex3  TGVVVPLF-K-KGGRRVCSNYRGITLLSLPGKVYARVLERRVRPIVKPQIQEQQCSFQPG
Oryzias-a03       RSVLVPIF-KtKGDMQNCSNYRGIKLMSHTMKLWERVVEARLRKKVE--ICEQQYGFMPR
Tetraodon-Rex3    TGVVVPLF-K-KGDRRMCSNYRGITLLSLPGKVYSGVLERRVRRIVEPQIQEEQCGFRPG
Arachis-a01       KSTLIPIY-KnKGDIQSCENYRGIKLMSDTIKLWERVIEQRLKKETH--VTENQFGFMPG
Glycine-a01       RSTLVPIY-KnKGDIQNCANYRGIKLMSHTMKLWERVIERRLRKETQ--VTENQFGFMPG
Medicag015        RSTLIPIY-KnKGDIQNCANYRGIKLTSHTMKLWERVIERRLRKETR--VTDNQFGFMPG
AtRTE-a01         RSILVPII--NRGDVQSCTNYHGIKLMSHTMKLWERIIEHRLRRMTS--VTKNQFGFMPG
HvRTE-a01         ------------------------------------------------------------
Solanum-a01       -STMVPLY-KnKGDIQNCNNYRGIKLLSHTMKIWERVVEMRVRREVS--ISENQFGFMPG
Xiphophorus-Rex3  TGVVVPLF-K-KGDRRVCSHYRGVTLLSLPGKVYSGVLERRVRRIVEPRIQEEQCGFRPG
Zea-a01           RSTLVPIF-KnKGDVQSCTNYRGIKLMSHTMKLWERVIEHRLRKMTS--VTQNQFGFMPG
Triticum-a01      RSILVPIF-KnKGDVQSCTNYRGIKLMSHTMKLWERVIEHRLRRMTS--VTKNQFGFMPG
Malus-a01         TSTLVPIY-KnKGDVQNCMNYRGIKLMSHTMKLWERVIEHRLRQETR--VSDNQFGFMPG
Eptatretus-a01    -SVLVPIF-KnKGDVQSCGNYRGIKLLSHTMKLWERVVEARLRGEVT--ICEQQYGFMPG
Bov-B_Vam         ----IPIP-K-RGSAKECSNYRTIALISHASKVMLKILQARLQQYVDRELPEVQAGFRRG
BmRTE-d01         RSIVVLFF-K-KGDKTLLKNYRPISLLSHVYKLFSRVITNRLAQRLDDFQPPEQAGFRRG
BmRTE-d04         RSRVVLFF-K-KGDKTLLKNYRPISLLSHVYKVFSRVITNRLARKLDECQPPEQAGFRKG
BmRTE-d05         ESLLIPLH-K-KGSTRTCSNYRTVALISHASKVMLYVINGRLQSYIQWQIPSEQAGFVKG
BmRTE-d06         HSIFIPLH-K-KGSTKKCNNYRLISLVSHASKVMLHIINTRLQGYLSREIAPEQAGFVKG
BmRTE-d08         KSVVVLFF-K-KGDNTRLKNYRPISLLSHVYKLFSRVITNRLARRFDDFQPPEQAGFRKG
BmRTE-d13         -STIVPIY-KgKGSKYECSNYRGIKLLSHTMKLYERVIDSRLRSECS--LSKNHYGFVQG
BmRTE-d14         TAVICPLH-K-KGDVLDCQNYRGISLLNTAYKIFANVLFGKLKPFVEPRLGEYQCGFRPG
BmRTE-d16         HSAILPLH-K-KGTTRRCDNYRTIALISHTSKVLLHVINSRLRHFLDWQIPQEQAGFVKG
```

```
BmRTE-d17           -GLLVKVP-K-KGDLSSCDNWKGITLLPCAAKVLARLLLNRMSKKMTGTLREEQAGFLPG
BmRTE-d20           TSTIILIH-K-KGRTDDISNYRPISLMSNIYKLFSKIVLERLTRTLDENQPKEQAGFRSG
BmRTE-d21           ESNMILLY-K-KGDPTDIGNYRPISLLPTLYKLFSSIIEKRISKRIEEHQPTEQAGFRRG
HmRTE-d01           -SFITPIY-KGrGSVQDCGSYRGIKLMSHTMKLFERMIDLRLRRECT--VSESQYGFQPG
BDDF                RSVFIPIP-K-KGNAKECSNYRTIALISHASKVMLKVLQARLQQYVNRELPDVQAGFRKG
JAM1                -GVICPIY-K-KGDKLECENYRAITILNAAYKVLSQILFRRLLPIANEFVGSYQTGFIDG
RTE-1               TSKTTLIF-K-KGDRENLENYRPICLLPVLYKVFTKCLLNRMRRSLDEAQP-EQAGFRRS
RTE-2               ---VKLIP-K-KAKATKIKDFRPISLLPILSKMFSSILTRRLTPTLESYLDESQNGFRKG
BmRTE-z01           KSTFIKIP-K-KQNAKKCGEYRMISLMSHVLKVFLNIIQNRIRPKCDEQLGDSQFGFRSG
BmRTE-z02           KSAIVPLH-K-KGSTAKCENYRTLSLMSHASKILLRIINSRLSAFIDHQIPREQTGFVAG
SR2                 ESIVVPIF-K-KGSRCSCNNYRGISLLSIASKLLASXILRRLFKTRERLTREEQAGFRSG
BmRTE-d12           ------LH-K-KGSTKDCNNYRTLALISHASKILLHIINSRIRHFLDWQIPQEQAGFVKG
Neurospora          RRIQIPKP--------GKKeTRPLTIASPRDKVVQKAIQLVMEPVFEKIFLDCSHGFRPH

Pristionchus-a01    SSTAEASFILSQLVDRHIEYRQGICAAFLDLEKAYDRTPRRQIWRSLrekgvpeiyvrli
Cbrenneri-a01       YGTNEHIFNVRMLIQKAREYKINLYIVFVDYQKAYDSVEWNSVFNTLeahgvdqtyvntl
Anolis-a01          CG---AIHATRLLIEKHPEKTRNIHTPFLDVEKAFDWIPPDLIWMLLrsqrvleeyiqwk
Aplysia-a01         KGCTDAIFALRQLCERALEYDKDLHLVFVDREKAFDRVNRNKLWKILeqydikgqlldni
Branchiostoma-a02   RGTTDAIFIVRQLQEKFIAANKPLYFAFVDLEKAFDRVPRRVLWWALrslgveewavrvi
Danio-a01           RGTLDQLYTLTRVLEGSWEYAQPVHMCFVDLKKAFDCVPRGILWRVLgeyGVRgnllrav
Gasterosteus-a01    RGTTDQLFTLSRIIEGAWEYAQPVYMCFVDLEKAYDRVPREILWEVLreygvrgsllgai
Mchenga-a01         RGTTDAIHAARLLLEKHREKSKTIHMAFLDLEKAFDRVPHDLIWHSLrshgvpeayvnwt
Nematostella-a01    KGTNDAIFILRQLQEKHLEKGKHLYLGFVDLEKAFDCVPREVLWWAMrrlsvpewlvstv
Oryzias-a06-Rex3    RGTVDQLYIISRVLQGLWEFAYTVHMCFVDLEKALDSVPLGILWRLLwengvrgallsav
Oryzias-a03         KSTTDAIFALRMLMEKYREGQKELHCVFVDLEKAYDRVPREELWYCMrssgvaekyvrvv
Tetraodon-Rex3      RGTVDQLYTLSRVLEGAWEFAQPIHMCFVDLEKAFDRVPRGVLWGVLreygvsglllqav
Arachis-a01         RSTTEAIYLLRRMMERYRSNKRDLHMVFIDLEKAYDRVPREVLWKVLekktvriayscai
Glycine-a01         RSTMEAIYLLRRVMEQYRMAQQDLHLIFIDLEKAYDRVPREILWKALekkgvrvayirai
Medicag015          RSTMEAIYLLRRGMERYRTDKKDLHLIFIDLEKAYDRVPREILWQALekkgvriayimai
AtRTE-a01           RSTMETIFLVRQLMERYREQKKDLHMVFIDLKKAYNKIPRNVMWWALEKHKVPAKYITLI
HvRTE-a01           ----------------------------------------------------------HI
Solanum-a01         RSTTEAIHLMRRLVEKYRERKRDLHMVFIDLEKAYDKVPRNVLWRCleakgipmiyirai
Xiphophorus-Rex3    RGTLDQLYTLSRVLEGAWEFAQPVYMCFVDLEKAFDRVPRGALWGVLreyGVPgplirav
Zea-a01             RSTMEAIFLLRQLMERFREQKKDLHMVFIDLEKAYDKVPRSVMWWALekhkvatkyinli
Triticum-a01        RSTMEAIFLVRQLMERYREQKKDLHMVFIDLEKAYDKIPRNVMWWALekhkvpakyitli
Malus-a01           RSTMEAIYLLRRLMERYRDGKKDLHMVFIDLEKAYDRVPRDILWRILekkgvrvayiqai
Eptatretus-a01      KSTTDAMFALRMLMKRYREGQKELHCVFVDLEKAYDRVPREELWHCMrksgvvekyvriv
Bov-B_Vam           RGTRDQIANIRWLMEKAREFQKNIYFCFIDYAKAFDCVDHNKLWQVLkemgvpdhlicll
BmRTE-d01           FGTVDHIHTVRQIIQKTEEYNLPLCLAFVDYEKAFDSIeiwavleslqrcqaDWRYIdaL
BmRTE-d04           YNTVDHIHTLRQIIEKSTEYNLPLCVAFVDYEKAFDSIEtWSVLeflqrcqidyryievL
BmRTE-d05           RGTREQIVNVRQIIEKSREFNMPILLCFIDYTKAFDCVRWDCLWRILremgvpqhlvsli
BmRTE-d06           RGTREQLLVMRQIVEKAREFNISLYVCFVDFRKAFDTVKWWKLWLVLtemgvpqhlvhti
BmRTE-d08           YSTVDHIHTLRQIVQKTEEYNLPLCLAFVDYEKAFDSIETWAMLQSLqrcqidyryievl
BmRTE-d13           LSTTDPMFALNTIAEEYREKLRPLYVAFLDMEKAFDRVPRDTIWWSLrkknvpehyvnvi
BmRTE-d14           RSTIDQIFSLRQILEKTLEFNADTYHLFIDFKAAYDNISRDFLYQAMheigvppklislt
BmRTE-d16           KGTREQILNVRLIIETCYEYNIPAVLCFVDYQKAFDCVSWKHLWYVLkdmgvpmhliqlm
BmRTE-d17           RSCTDHTNTLRILIEQSVEWQTEMILTFVDFEKAFDTVQWSKMWTCLkqrgipnkiigim
BmRTE-d20           FSTLDHIHTIKQIIQKCNEYNINYYLSFIDYNKAFDSLKHQKIWEALalqgvhnkyirll
BmRTE-d21           YSTIDHIHAIEQIVEKYNEYQKPLYVVFIDYRKAFDTIRHSSIWTALisqrvehkyieii
HmRTE-d01           SGTMDAIFALRTLMEAYREKRRALHVAFLDLQKAFDCVPRQCIWWALrskgipeayieii
BDDF                RGTRDQIANICWIMEKAREFQKNIYFCFIDYAKAFDCVDHNKLWKILkemgipdhltcll
JAM1                RSTMYWIFSVRQILQKCREYQVPTHHLFIDFKAAYDSIDRIELWKIMdensfpgkltrli
RTE-1               FSTIDHIHSLQRLLEVGREYQIPLTLVFIDFKKAFDSVEHQAIWKSLdeqgadgayidll
RTE-2               RCCADNIQSLTMLIEKCNEFQLPLLLLFIDYQTAFDKIGHSAVVSSLekagadpamrkmi
BmRTE-z01           VSTREALFALNVLVQKCRDMQTDVFLCFIDYEKAFDRVKHHQLFSLLcdigldgkdvrii
BmRTE-z02           KGTREQILNVRVLIEKFREFNKPLVLCFIDYAKAFDCVRWSSMFDVLidmgvpshlilli
SR2                 RGCIDHIFTLRQMLEHRHTYRRPTIVVFLDIRAAFDSLDRTVLWDCLlkkgvpekfinil
BmRTE-d12           RGTREQILNIRQLIERCHEFDTPIILCFVDYSKAFDCVGWNCLWRVLqelgvpmhlkafl
```

```
Neurospora         RGTKTAIQYVDAKFQSSH-F-----IIEADFSKAFDSIAHSKLMEFLKETITCEKTLKLI

Pristionchus-a01   kemyegasaqvrtpfgpt-dditi-kvGVHQGSALSPLLFITVLDSVMGD-T--------
Cbrenneri-a01      eaiykeatskikihe-et-tavsi-krGVRQGCVLSPLLFNAVLEEVFRN-L---N-W--
Anolis-a01         nmlyvnltsvircaggis-dpfpv-qaGVLQGLALSPYLFILCMDAITGD-I--------
Aplysia-a01        raiyansrsavrttsgts-dwfpv-tsGVRQGCNLSPLLFVIYMDQILKE-A-NPD----
Branchiostoma-a02  qamyanarsrvrvngqys-eefgv-gvGVHQGSVLSPLLFILVLEALSRE-F--------
Danio-a01          sslyeqsrslvciagnkairlfsv-hvglwQGCPLSPILFIIFMDRISRC-S-LGL----
Gasterosteus-a01   qslyaqsescvrvlgsks-kafpv-gvGLRQGCALSPILFVVFMDRISRR-S-RGE----
Mchenga-a01        kllyrqvtsvvrspvgts-ppfni-nvGVHQGSALSPLLFILCMDTATAD-L--------
Nematostella-a01   kamysnassrvrvdnsys-dnfnv-qvGVHQGSVLSPLLFIIVLEALSCD-L--------
Oryzias-a06-Rex3   rslydwskslvrkagsks-dlfpv-hvGLRQGCPLPPVLFIVFMDRICRR-S-QGP----
Oryzias-a03        qdmyersmtvvrcavgqt-eefkv-evGLHQGSALSPFLFAMLMDRLTDE-V--------
Tetraodon-Rex3     rslynrcqslvriagsks-nsfpv-rvGLRQGCPLSPILFITFMDRISRR-S-YGV----
Arachis-a01        kdmydgattsvktqggvt-kkfli-siGLYQGSSLSPYLFTLVLEVLTKKYI--------
Glycine-a01        qdmydrvstsvrtqgges-ddfpi-tiGLHQGSTLSPYLFTLILDVLTEQ-I--------
Medicag015         kdmyegastsvrthdett-edfpt-tiGLHQGSTLSPYLFTLVLDVLTEH-I--------
AtRTE-a01          KDMYDNVVTSVRTSDVDT-NDFPI-KIGLHQGSALSPYLFALVMDEVTRD-I--------
HvRTE-a01          KDMYDNVVTSVRTSDGDT-DDFPI-KIGLHQGSALSPYLFALVMDEVTRD-I--------
Solanum-a01        kdmyggaktrvrtvggds-ehfpv-eMGLHQGSVLSPFLFALVMDELTRS-I--------
Xiphophorus-Rex3   rslydrcqslvriagsks-gsfpv-rvglcQGCPLSPILFITFMDRISRR-S-QGV----
Zea-a01            kdmytnvvtsvrtsdgdt-ddfpi-niGLHQGSALSPYLFALVIDEVTRD-I--------
Triticum-a01       kdmydnvvtsvrtsdvdt-ddfpi-kiGLHQGSALSPYLFALVMDEVTRD-I--------
Malus-a01          kdmyegaktavrthegqt-esfpi-tvGLHQGSSLSPYLFALVMDELTGH-I--------
Eptatretus-a01     qdmyedsvtavrcavgmt-drfkv-kvGLHQGSTLSPFLFAMVMDRLTDE-I--------
Bov-B_Vam          rnlyagqeatvrtghgtt-dwfki-gkGVRQGCILLPCLFNLYAEHIMRK-AgldE-S--
BmRTE-d01          RCLydtatmtvqvqkdqt-rpiql-rrGVRQGDIISPKLFTNALEDVFKT-L---D-W--
BmRTE-d04          KSLyeaatmtvqiqdcqs-ipikl-qrGVRQGDVISPKLFTAALEDVFKT-L---D-W--
BmRTE-d05          aslyrdgvsmvrvndvis-gpfkp-ekGVRQGCILSPILFNVYGEYVMRK-A-leE-W--
BmRTE-d06          rrlyedgtaavrvdsids-erfst-qaGVRQGCILSPLLFNIYTEYIMRI-V-ldD-W--
BmRTE-d08          rclyenatmsvrvqdras-epill-qrGVRQGDVISPKLFTAALEDVFKV-L---D-W--
BmRTE-d13          idmyrdarsmvrtvvgqt-kpiav-aeGLHQGSVLSPFLFGMVIDSLTEV-A--------
BmRTE-d14          rmtlvasqsivkiqtdls-dpfrt-hdGLRQGDALSCLLFNVALDKCIRD-S-AIE----
BmRTE-d16          ralylsgrgsvrigpaqs-refrf-ekGVRQGCIVSPILFNIYGEYIMRK-T-leE-W--
BmRTE-d17          qalyrgstcrvvhdqvlg-apiem-taGVKQGCLLSPLLFIMLLDDIMRE-V-VTT-P--
BmRTE-d20          kniyenmkarvrtek-lg-ehfhi-kkGVRQGDPLSPKLFSATLEHVFRQ-L---E-W--
BmRTE-d21          kylynnctsrvklet-tg-ppipi-rrGVRQGDPLSPKIFIAVLEMVFSK-L---N-W--
HmRTE-d01          rgmyhdsasmvrtavgdt-rpfpi-tvGVHQGSALSPFLFNVVLDTVSAH-I--------
BDDF               rnlyagqeatvrtghgtt-dlfqi-gkGVCQGCILSPCLFNFYAEYIMRN-AgleE-A--
JAM1               ratmdgvqncvki-sghs-ssfes-rrGLRQGDGLSCM------SCCHAE-S-LYL----
RTE-1              kecykncttnftpfh-rp-vavpv-tkGVRQGDPISPNLFSACLEHVFRK-L---S-WIE
RTE-2              qemmdggqaeitvhd-kk-lkvnl-ctGVRQGDSASPALFSAALQAILTD-C---D-N-E
BmRTE-z01          rnlyekqvatirveneet-dqvei-crAVRQGCVLSPLLFNIYSEAVMSK-A-LEn-l--
BmRTE-z02          rnlyldgscfvkldnrrs-rsfht-ehGVRQGCILSPKLFNIYGEYIMRR-A-LEG-W--
SR2                kalytntsgrvraynhls-plfhs-ssGVRQGCPISPFLFNFAIDDILET-A-LMDVS--
BmRTE-d12          qslyygsqgtvrvdytms-nrfnf-rrGVRQGCILSPILFNIYGEYIMRK-T-leN-W--
Neurospora         RSGLKAGYIEFG-------ELHNNlDIGTPQGSILSPLLCNIFLHRLDLF-M-------E

Pristionchus-a01   ---M----------------E---------------------------------------
Cbrenneri-a01      --------EEnseyGLKVN-G---------------------------------------
Anolis-a01         ---E----------------K---------------------------------------
Aplysia-a01        --------------------P---------------------------------------
Branchiostoma-a02  ---R----------------T---------------------------------------
Danio-a01          -------------EGVRFG-E---------------------------------------
Gasterosteus-a01   -------------EGLQFG-G---------------------------------------
Mchenga-a01        ---Q----------------S---------------------------------------
Nematostella-a01   ---R----------------R---------------------------------------
Oryzias-a06-Rex3   -------------GGVWFR-D---------------------------------------
```

```
Oryzias-a03         ---R----------------Q---------------------------------------
Tetraodon-Rex3      -------------EGVRFG-D---------------------------------------
Arachis-a01         ---K----------------E---------------------------------------
Glycine-a01         ---Q----------------E---------------------------------------
Medicag015          ---Q----------------E---------------------------------------
AtRTE-a01           ---Q----------------G---------------------------------------
HvRTE-a01           ---Q----------------G---------------------------------------
Solanum-a01         ---Q----------------E---------------------------------------
Xiphophorus-Rex3    -------------EGIRFG-G---------------------------------------
Zea-a01             ---Q----------------G---------------------------------------
Triticum-a01        ---Q----------------G---------------------------------------
Malus-a01           ---Q----------------D---------------------------------------
Eptatretus-a01      ---R----------------L---------------------------------------
Bov-B_Vam           ------K------VGIKIA-G---------------------------------------
BmRTE-d01           --------NG---rGININ-G---------------------------------------
BmRTE-d04           --------kT---CGINVN-G---------------------------------------
BmRTE-d05           ------E------GGISVG-G---------------------------------------
BmRTE-d06           ------D------KGISVG-G---------------------------------------
BmRTE-d08           --------kG---LGININ-G---------------------------------------
BmRTE-d13           ---Q----------------S---------------------------------------
BmRTE-d14           ------T------TGNIYY-----------------------------------------
BmRTE-d16           ------D------GGVTVG-G---------------------------------------
BmRTE-d17           -------------RGIEWS-E---------------------------------------
BmRTE-d20           --------DD---yGININ-G---------------------------------------
BmRTE-d21           --------ER---KGLNIN-G---------------------------------------
HmRTE-d01           ---Q----------------D---------------------------------------
BDDF                ------Q------AGIKIA-R---------------------------------------
JAM1                ------T------VEARFS-----------------------------------------
RTE-1               lkgeaedydt-i-pGMRVN-G---------------------------------------
RTE-2               f-------ag---VGINVE-G---------------------------------------
BmRTE-z01           ------E------VGIGIN-G---------------------------------------
BmRTE-z02           ------n------gGISVG-G---------------------------------------
SR2                 ------N------GgvdmLPG---------------------------------------
BmRTE-d12           ------D------GGITIG-G---------------------------------------
Neurospora          SI-K---------AEFNIG-vkkkrskeymalmnkcrymrskgqdisnpelyhairnkml

Pristionchus-a01    -----------KAPNCLAYADDLCLIDT-DV-GSLERKVQEVQ---RRL-QAGGLTLNTG
Cbrenneri-a01       -----------ERITNLRYADDIALIAT-NK-NTMQKMMDELV---ERS-REVGLRMNKK
Anolis-a01          -----------PQHW-MLLMRDAMLATE-SW-VELQTQVQVWK---NHL-QYFGLWLNIS
Aplysia-a01         -----------EALNELMFADDLAMI-N-NK-TQLQEHINQLN---ASC-EKHDMKISIS
Branchiostoma-a02   -----------GVPWELLYADDLVIIAD-TL-EECIARLKAWk---sgM-ERKGLRVNMG
Danio-a01           -----------HRISSLLFADDVVLLAS-SD-MDLHHALGRFA---AEC-DAAGMRISTS
Gasterosteus-a01    -----------LRISSLLFADDVVLMAS-SV-CDLQLSLERFA---VEC-EAVGMRISTS
Mchenga-a01         -----------PHPWTLLYADDVFLADE-SR-IELQNQTQQWK---TRL-ADFGLRLNTN
Nematostella-a01    -----------GCPWELLYADDLVIASD-SL-ENLQQKLMLWK---TGM-eskGLRVNMK
Oryzias-a06-Rex3    -----------HRISSLLFADDVFLLAS-SG-QDLQRTLGRFA---NEC-EAAGMRFNAS
Oryzias-a03         -----------ESPWTMMFADDIVICSE-SR-EQVEEQLERWR---FAL-ERRGMKVSRS
Tetraodon-Rex3      -----------SRIGSLLFADDVVLLAS-ST-RDLQLPLDRFA---AEC-EASGMRISTS
Arachis-a01         -----------LVPWCMLFADDIVLMGE-LR-EDLNKKLNLWR---KTL-KVYGLCISRS
Glycine-a01         -----------IAPRCMLFADDIVLLGE-SR-EELNERLETWR---RAL-ETHGFRLSRR
Medicag015          -----------LAPRCMLFADDVVLVGE-SR-EEVNGRLESWR---QAL-EAYGFRLSRS
AtRTE-a01           -----------DIPWCMLFVDDLVLVDD-SR-AGVNNKLELWR---QTL-ESKGFRLSRT
HvRTE-a01           -----------DIPWCMLFADNVVLVDD-S-RTGVNRKLELWR---QTL-KSKGFRLSRT
Solanum-a01         -----------RVPWCMLFADDIVLIDE-TR-DRADARLEVWR---QTL-ESKGFKLSRT
Xiphophorus-Rex3    -----------LRISSLLFADDVVLLAS-SG-HDLQLSLERFA---AEC-EAAGMGISAS
Zea-a01             -----------VLPWCMLFADDVVLIEE-SR-SGVSQKLELWR---QTL-EAKGFRLSRS
Triticum-a01        -----------DIPWCMLFADDVVLVDD-TS-AGVNRKLELWR---QTL-ESKGFRLSRT
Malus-a01           -----------DIPWCMLFADDIVLIDE-TQ-EGVNAKLNLWR---EVL-ESKGLRLSRS
```

```
Eptatretus-a01    -----------ESPWTMMFADDIMICRE-SR-EQAEASLERWR---YAL-ERRGMIVSRS
Bov-B_Vam         -----------RNINNLRYADDTTLMAE-SE-EELKSLLLRVK---KES-AKLGLKLNIK
BmRTE-d01         -----------EYISHLRFADDIVIMAE-SL-QDLQEMVHSLN---AAS-QRVGLGMNLD
BmRTE-d04         -----------EYMSHLRFADDIVLMSE-SL-EDLSRMLNDLN---AAS-RCVGLRMNLD
BmRTE-d05         -----------IKISNLRYADDTTLFAS-SE-KELADLFRRVE---YES-SLVGLSVNKS
BmRTE-d06         -----------RKISNLRYADDTTLLAS-TR-DEIEVLLRRLE---TTA-LDFGLAINRD
BmRTE-d08         -----------EYITHLRFADDIVIMAE-TM-EDLSTMLKDLS---RAS-IRVGLNMNKE
BmRTE-d13         -----------SASWTFIYADDVAICTE-SR-TKLREALLLWK---QQL-QAGGLILSVA
BmRTE-d14         -----------KSAQVLGYADDIDVIGR-SA-LAVESAYLALE---ASS-LEAGLQVNAD
BmRTE-d16         -----------VKISNLRYADDTTLVAS-SE-EEMEELLRRLV---IVS-EEIGLKINQS
BmRTE-d17         -----------NIlEDLDYADDIVLMTP-TL-DQMQAKLEDLR---MSA-EKRGLRINTN
BmRTE-d20         -----------VLLNHLRFADDLILISE-NP-ETLQKMIEQLV---RES-EKVGLSLNTS
BmRTE-d21         -----------NfINHLRFADDIILLSE-SA-KEMESMIHSLK---TMS-CEVGLEMNLD
HmRTE-d01         -----------QPPWLMMYADDIALIAE-NR-LTLERKVNLWK---GTL-ENGGLKLNVS
BDDF              -----------RNINNLRYADDTTLMAE-SE-EELKSLLMKVK---VES-EKVGLKPNIQ
JAM1              -----------RDPYNLFFADDMMLLGE-NL-KQWSDLFTRLK---REA-TRVGLMVNVS
RTE-1             -----------RNLTNLRFADDIVLIAN-HP-NTASKMLQELV---QKC-SEVGLEINTG
RTE-2             -----------RHIRRLEFADDVVLICS-TP-GEVQERLEILD---RIS-SNYGLKINQS
BmRTE-z01         -----------RVVNNLRYADDTILIAA-SE-ADLQAIVNKVN---ECS-EEAGLSINIS
BmRTE-z02         -----------ETLTNLKYADDTTMLAS-DE-EEMAILLNRVE---EES-AKLGLII---
SR2               -----------ERLLDLEYADDIVLLCD-NA-QGMQSALNQLA---ISV-RRYGMCFAPS
BmRTE-d12         -----------VKVTNLRYADDTTLLAT-TE-AEMTELLNRME---HIG-LEMGLALNRS
Neurospora        ttpsvTKDDSYVRVNYVRYADDFIIGVE-GShKTAVAILEKVQ---SFVtNQLGLRLNPD

Pristionchus-a01  KTEFMMI-----GGG---QG------M-----MRD-MKG----E--EIKKVEVFRYLGSL
Cbrenneri-a01     KTVALTn-----------IS-SGPN---E-DLSIQ-LDG----Q--KVEMVKSFTYLG--
Anolis-a01        KSEYMEYGPTV-------ED------G-----SIN-IND----T--DLNKVTSFKYL---
Aplysia-a01       KTEVMTI-----------SR-RPGK-----V-DIN-ING----S--QLKQSREFKYLGSI
Branchiostoma-a02 KTKIM-------------------------------------------------------
Danio-a01         KSEAMVL-----------HR-KKVV-----C-HLQ-VGG----K--SLPQV-EFKYLWVL
Gasterosteus-a01  KSEAMVL-----------SR-KPMD-----C-LLQ-VGN----V--SLPQVKEFKYLGVL
Mchenga-a01       KTEYLEA-----GPQ---TD------G-----TIS-VDG----E--DLAKVPHFKYLGSM
Nematostella-a01  KTKVMVSGPNLETLK-D-------------------------------------------
Oryzias-a06-Rex3  KFEAMVL-----------DR-RKVV-----C---H-VVG----V--SLPQVEEFKYLGVL
Oryzias-a03       KTEYM-C---LNERD---QG------R-----SVR-LQG----T--EVKKVQEFKYLGST
Tetraodon-Rex3    KSEAMVL-----------NR-KKVE-----C-LLR-VGE----E--NLPQVEEFKYLGVL
Arachis-a01       KTKYMECKfglRREN---PN------I-----EVK-IGE----N--IIQKVKSFKYLGCI
Glycine-a01       KSEYMECkfnKRRRV---SN------S-----EVK-IGD----H--TIPQVIRFKYLGSV
Medicag015        KTEYMECnfsGRRSS---ST------L-----EVK-VGD----H--IIPQVTRFKYLGSF
AtRTE-a01         KTEYMMCGFSTTRCE---EE------------EVS-LDG----Q--VVPQKDTFRYLGSM
HvRTE-a01         KTEYMRCDFSTTKHE---DG------------GGVSLDG----Q--VAPQKDTFRYLGSM
Solanum-a01       KTEYLGCKFSdalDE---AD------G-----DVR-LAT----Q--IIPKKESFKYLGFV
Xiphophorus-Rex3  KSEAMVL-----------SR-KRVE-----C-LLR-VKG----G--VLPQVEEFKYLGIL
Zea-a01           KTEYMKcdFSAMGYE---DG------------DVS-LDG----Q--VVPKKDTFRYLGSM
Triticum-a01      KTEYMMcgFSTTRCE---EE------------EVS-LDG----Q--VVPRKDTFRYLGSM
Malus-a01         KTEYMECNfsaNGGQ---NE------L-----GVR-IGD----Q--EIPKSDRFRYLGSI
Eptatretus-a01    KTEYM-C---VNERE---GG------G-----MVR-LQG----V--EVGKVDGFKYLGST
Bov-B_Vam         KTKIMAS-----------NP----L-----N-SWQ-IDG----E--EMEVVTDFIFLGSK
BmRTE-d01         KTKVMFN-----------GN-VIPR-------PID-VGG----T--PLEVVQEYIYLGQT
BmRTE-d04         KTKVMFN-----------DK-IVPG-------QVT-ISN----A--VIEEVD-FVDLGQA
BmRTE-d05         KTKVMIV-----------DR----T-----S-QLS-RTG----ELSDLEFVSEFIYLGSL
BmRTE-d06         KTKMMIV-----------DR----A-----NINQP-EVQ----HIAGCEVVNSYVYLGST
BmRTE-d08         KTKIMLN-----------AH-VAPT-------PVK-IGG----S--TLEVVDEYIYLGHT
BmRTE-d13         KTHYMSF-----NdPDPGDN------S-----PIS-IDG----Q--LVNMCDQYKYLGTM
BmRTE-d14         KTKFLRV-----------SR-DlREDT---A-HKN-IGQ----H--TFGSVNEFVYLG--
BmRTE-d16         KTKIMIV-----------DK----Y-----G-TLN-ENN----ILGQYDIVKTFVYLGSI
BmRTE-d17         KTVDMRV-----------MS-KNTT-----P--LK-LQD----C--VLKSAQKFTYLGSS
BmRTE-d20         KTKLMTN-----------YK-KVP---------IKPYNT----A--KLEYVNEYTYLGQI
```

```
BmRTE-d21         KTKIMSN-----------SI-KHP---------IY-LDE----K--PLEYVDSYIYLGKQ
HmRTE-d01         KTEYMAC-----GSR---DS------S-----TIL-IGP----E--PAVKSEKFRYLGSV
BDDF              KMKIMAS-----------GP----I-----T-SRE-IDG----E--TVETVSDFMFLGSK
JAM1              KTKYMLV-----------GG-TECDRARlgs-sVT-IDG----D--TFEVVDEFVYLGSL
RTE-1             KTKVLRN-----------RF-ADPS-------EVY-FGspSPTT--QLDDVDEYIYLG--
RTE-2             KTVLLKN-----------KF-CRSQ-------DVL-FNG----S--PIIPVPGCRYLGRW
BmRTE-z01         KTKFMVV-----------SRNPDLS-----S-SVS-VAG----K--QLERVRQYKYLGAW
BmRTE-z02         ---IMII-----------DR----A-----Q-LFP-RSD----ALSGYEKVAEFIYLGSQ
SR2               KCKVLLQ-----------DW-QDSH-----P-VLT-LDG----E--QIEVVEKFVYLGSY
BmRTE-d12         KTKIMVV-----------DR----T-----K-KLE-LSG----TL-NLELVDNFIYLGSN
Neurospora        KTGITKY-----------------------S-----VDP--------------VKFLGY-

Pristionchus-a01  I
Cbrenneri-a01     -
Anolis-a01        -
Aplysia-a01       F
Branchiostoma-a02 -
Danio-a01         F
Gasterosteus-a01  F
Mchenga-a01       I
Nematostella-a01  -
Oryzias-a06-Rex3  F
Oryzias-a03       V
Tetraodon-Rex3    F
Arachis-a01       I
Glycine-a01       I
Medicag015        V
AtRTE-a01         L
HvRTE-a01         L
Solanum-a01       I
Xiphophorus-Rex3  F
Zea-a01           L
Triticum-a01      L
Malus-a01         L
Eptatretus-a01    V
Bov-B_Vam         I
BmRTE-d01         L
BmRTE-d04         I
BmRTE-d05         L
BmRTE-d06         I
BmRTE-d08         V
BmRTE-d13         M
BmRTE-d14         -
BmRTE-d16         I
BmRTE-d17         I
BmRTE-d20         I
BmRTE-d21         I
HmRTE-d01         M
BDDF              I
JAM1              L
RTE-1             -
RTE-2             -
BmRTE-z01         V
BmRTE-z02         I
SR2               I
BmRTE-d12         I
Neurospora        -
```
